# Development of new TAK-285 derivatives as potent EGFR/HER2 inhibitors possessing antiproliferative effects against 22RV1 and PC3 prostate carcinoma cell lines

**DOI:** 10.1080/14756366.2023.2202358

**Published:** 2023-04-25

**Authors:** Seohyun Son, Ahmed Elkamhawy, Anam Rana Gul, Ahmed A. Al‐Karmalawy, Radwan Alnajjar, Ahmed Abdeen, Samah F. Ibrahim, Saud O. Alshammari, Qamar A. Alshammari, Won Jun Choi, Tae Jung Park, Kyeong Lee

**Affiliations:** a College of Pharmacy, BK21 FOUR Team and Integrated Research Institute for Drug Development, Dongguk University-Seoul, Goyang, Republic of Korea; b Department of Pharmaceutical Organic Chemistry, Faculty of Pharmacy, Mansoura University, Mansoura, Egypt; c Department of Chemistry, Research Institute of Chem-Bio Diagnostic Technology, Chung-Ang University, Seoul, Republic of Korea; d Pharmaceutical Chemistry Department, Faculty of Pharmacy, Ahram Canadian University, Giza, Egypt; e Department of Chemistry, Faculty of Science, University of Benghazi, Benghazi, Libya; f Faculty of Pharmacy, Libyan International Medical University, Benghazi, Libya; g Department of Chemistry, University of Cape Town, Rondebosch, South Africa; h Department of Forensic Medicine and Toxicology, Faculty of Veterinary Medicine, Benha University, Toukh, Egypt; i Department of Clinical Sciences, College of Medicine, Princess Nourah bint Abdulrahman University, Riyadh, Saudi Arabia; j Department of Plant Chemistry and Natural Products, Faculty of Pharmacy, Northern Border University, Arar, Saudi Arabia; k Department of Pharmacology and Toxicology, Faculty of Pharmacy, Northern Border University, Arar, Saudi Arabia

**Keywords:** EGFR/HER2, chemical synthesis, apoptosis, kinase panel, prostate carcinoma

## Abstract

Epidermal growth factor receptor (EGFR) and human epidermal growth factor receptor 2 (HER2) protein tyrosine kinases co-expressed in various cancers such as ovarian, breast, colon, and prostate subtypes. Herein, new TAK-285 derivatives (**9a**–**h**) were synthesised, characterised, and biologically evaluated as dual EGFR/HER2 inhibitors. Compound **9f** exhibited IC_50_ values of 2.3 nM over EGFR and 234 nM over HER2, which is 38-fold of staurosporine and 10-fold of TAK-285 over EGFR. Compound **9f** also showed high selectivity profile when tested over a small kinase panel. Compounds **9a**–**h** showed IC_50_ values in the range of 1.0–7.3 nM and 0.8–2.8 nM against PC3 and 22RV1 prostate carcinoma cell lines, respectively. Cell cycle analysis, apoptotic induction, molecular docking, dynamics, and MM-GBSA studies confirmed the plausible mechanism(s) of compound **9f** as a potent EGFR/HER2 dual inhibitor with an effective antiproliferative action against prostate carcinoma.

## Introduction

**Figure 1.**
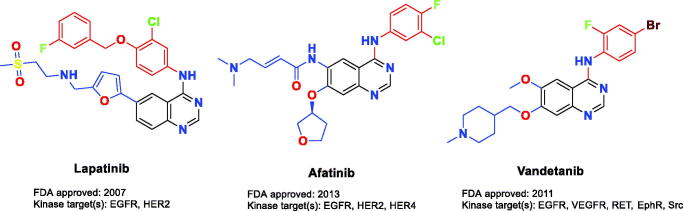
FDA-approved multikinase kinase inhibitors.

Epidermal growth factor receptor (EGFR) and human epidermal growth factor receptor 2 (HER2) protein tyrosine kinases co-expressed in various cancers such as ovarian, breast, colon, and prostate subtypes^1–4^. In 20–25% of human breast cancers, HER2 gene amplification and receptor overexpression are observed^5^. EGFR/HER2 small molecule inhibitors could prevent tyrosine kinase phosphorylation, which in turn suppresses the upregulated intracellular signalling in solid tumours, consequently, the dysfunction of tumour regulation occurs. Many ATP-competitive EGFR/HER2 RTK (receptor tyrosine kinase) dual small molecule inhibitors bearing diverse chemical scaffolds are widely tested in human clinical studies for cancer therapy. The FDA-approved small molecule lapatinib (Figure 1) is prescribed to treat patients with HER2 overexpression metastatic breast cancer. It possesses a 4-anilinoquinazoline scaffold which is a promising chemical moiety for EGFR/HER2 dual inhibition^6^. In the literature, the interaction of lapatinib with the catalytic domain of EGFR/HER2 kinases has been well studied. Generally, the hinge region is hydrogen bound to the quinazoline ring, which is located at the ATP binding site. In order to establish additional hydrophobic interactions, the aniline moiety at the C4 position on the quinazoline scaffold is directed to bind with a neighbouring back pocket^7^^,^^8^. In the previous research, it has demonstrated that the size and functionality of this hydrophobic pocket have a decisive effect on the kinase inhibitor selectivity, while the substituents on C5 and C6 positions could enhance the physical properties to attain favourable pharmacokinetics of the quinazoline-based scaffold. Furthermore, several dual inhibitory candidates were designed to bind to Cys805 in HER2 and Cys773 in EGFR^9–13^. Even though lapatinib treatment was found beneficial, many patients did not show a positive response to it or acquired resistance for diverse undiscovered causes^14–17^. For this reason, new therapeutics using novel small molecule inhibitors are needed for EGFR/HER2 suppression.

Since the 1960s, 2-nitroimidazole derivatives have been used as chemotherapies and hypoxia-activated radio-sensitisation therapy^18^. In the presence of hypoxia, the 2-nitroimidazole is reduced by nitroreductase to generate reactive radicals, and it could exhaust tumour-specific antioxidants such as glutathione (GSH), which would make tumours more susceptible to radiotherapy^19^. Moreover, the reactive radicals build up in cells and induce lethal consequences because of their irrevocable binding to the nucleic acid and protein^20^.

**Figure 2.**
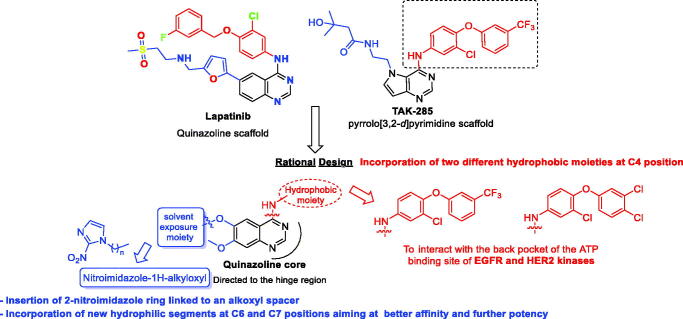
Rational design of the new TAK-285 derivatives **9a**–**h**.

Up to date, many potential antitumor small molecule inhibitors bearing 2-nitroimidazole moiety have been discovered^21–23^. Recently, our group reported a series of lapatinib derivatives possessing 6-(nitroimidazole-1*H*-alkyloxyl) moiety with potent dual EGFR/HER2 kinase inhibitory activities^24^. Consequently, we focus in this research on development of this hybrid scaffold (Figure 2) to selectively inhibit EGFR/HER2 tyrosine kinases as well as to assess the antiproliferative activity of this new series against prostate carcinoma cell lines. Herein, two different aniline moieties (3-chloro-4-(3-(trifluoromethyl)phenoxy)aniline and 3-chloro-4-(3,4-dichlorophenoxy)aniline) were incorporated at C4 position in a fashion similar to the potent dual inhibitor TAK-285 (Figure 2)^25^. A variety of polar/solubilising nitroimidazole moieties linked to alkoxy linkers with different lengths were added to C6 and C7 positions of the quinazoline scaffold. The inhibition profile over EGFR and HER2 kinases of all the synthesised compounds (**9a**–**h**) was assessed. The IC_50_ values of the highly active candidate were evaluated over both kinases. In addition, to verify the selectivity of the most active candidate, a small kinase panel was then employed. Cell-based antiproliferative evaluation was carried out over two prostate carcinoma cell lines. Moreover, comprehensive simulation analyses were accomplished to recognise the binding affinities and direction of the final small molecules.

## Results and discussion

### Chemical synthesis

**Scheme 1.**
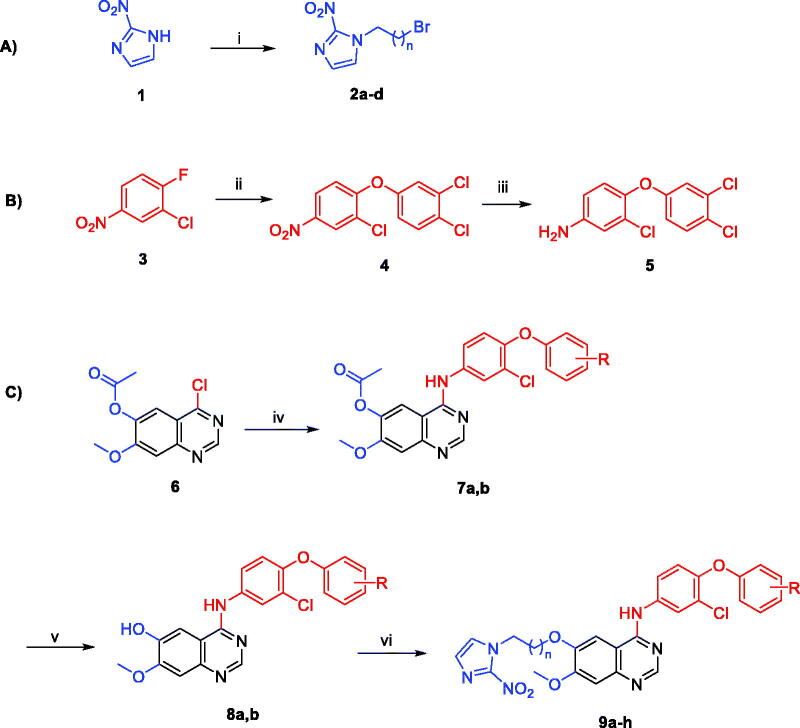
Reagents and conditions: (i) 1,n-dibromoalkane, K_2_CO_3_, DMF, 60 °C, 4 h; (ii) 3,4-dichlorophenol, K_2_CO_3_, acetonitrile, 85 °C, 3 h; (iii) 10% Pt/C, H_2_ gas, methanol, rt, 18 h; (iv) aniline reagent, isopropyl alcohol, reflux, 4 h; (v) aqueous ammonia solution (28%), methanol, rt, 4 h; (vi) 1-(n-bromoalkyl)-2-nitro-1*H*-imidazole derivative, K_2_CO_3_, DMF, 80 °C, 4 h.

Scheme 1(A) demonstrates the chemical synthesis of compounds **2a**–**d** via reacting 2-nitroimidazole (**1**) with different 1,n-dibromoalkanes in DMF solvent and K_2_CO_3_ at 60 °C. In Scheme 1(B), 2-chloro-1-fluoro-4-nitrobenzene (**3**) reacted with 3,4-dichlorophenol in the presence of K_2_CO_3_ and acetonitrile solvent for 3 h at 85 °C to afford 1,2-dichloro-4-(2-chloro-4-nitrophenoxy)benzene (**4**). The nitro group of intermediate **4** was then reduced via 10% platinum on carbon and H_2_ gas at room temperature to afford 3-chloro-4-(3,4-dichlorophenoxy)aniline (**5**). The second aniline reagent (3-chloro-4-(3-(trifluoromethyl)phenoxy)aniline) was purchased. In Scheme 1(C), 4-chloro-7-methoxyquinazolin-6-yl acetate (**6**) reacted separately with the two aniline reagents to form intermediates **7a** and **7b**. Intermediates **8a** and **8b** were then produced by hydrolysis of the acetate group of compounds **7a** and **7b** utilising 28% aqueous ammonia in methanol solvent. The free phenolic group in compounds **8a** and **8b** was allowed to react with the imidazoles **2a**–**d** in DMF solvent and K_2_CO_3_ at 80 °C to generate the desired TAK-285 derivatives (**9a**–**h**) (Table 1).

**Table 1.** Chemical structures and isolated yields of compounds **9a**–**h**.

Cpd	Structure	Yield (%)
**9a**	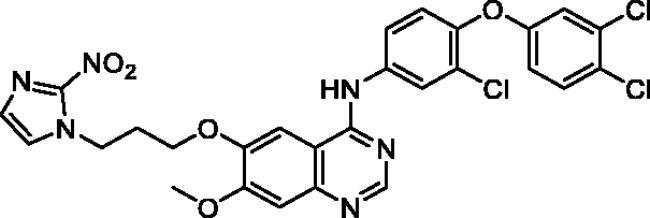	75.2
**9b**	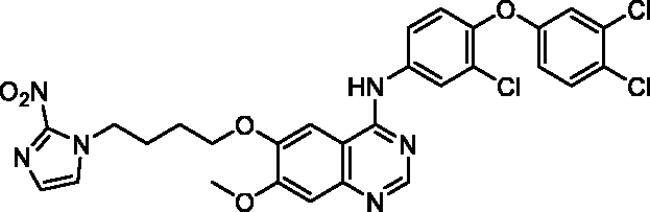	53.4
**9c**	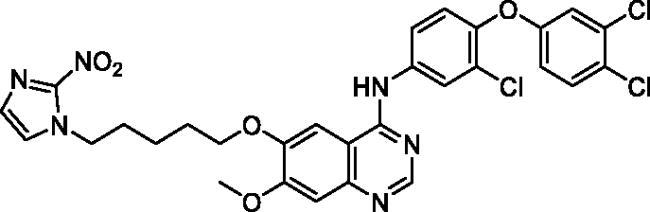	69.3
**9d**	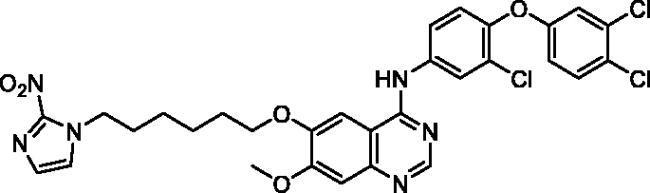	56.0
**9e**	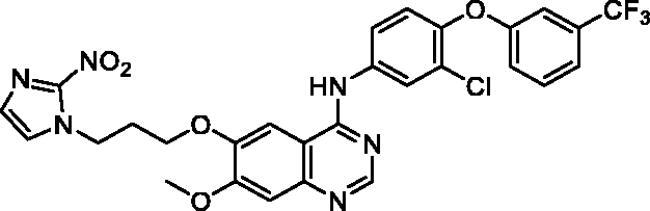	71.8
**9f**	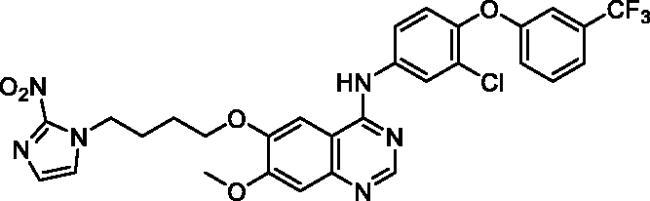	38.3
**9g**	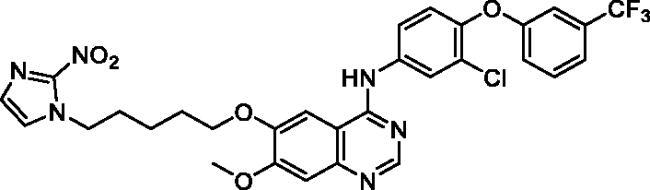	48.7
**9h**	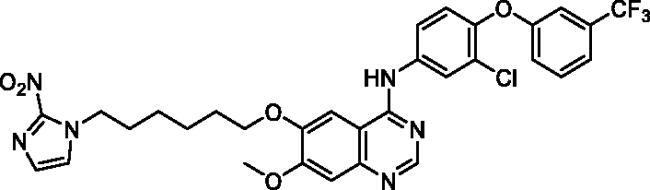	62.0

### Structure elucidation of the newly synthesised TAK-285 derivatives 9a–h

To elucidate the chemical structure of compounds **9a**–**h**, various spectroscopic approaches were used including ^1^H NMR, ^13^C NMR, and HRMS. Also, their purity was acquired via the HPLC system where they showed purity higher than 95%. Since all final compounds have a methoxy group, a singlet peak at the 3.95–3.92 ppm range was observed in the ^1^H NMR spectra. In addition, the specific peak of C2 position proton of quinazoline scaffold was found at 8.54–8.51 ppm (see supporting information). Furthermore, the extended alkyl linkers of all final compounds (*n* = 2–5) were confirmed where their protons and carbons were found in relatively low chemical shifts. In the ^1^H NMR spectrum, compound **9a** showed the C2 of quinazoline at 8.52 ppm, and three protons of the methoxy group at 3.92 ppm as a singlet peak. Additionally, the O-alkylation reaction between the OH group and the imidazole linker (step vi) was proved by the disappearance of the OH peak. Like compound **9a**, the target compounds **9b**, **9c**, and **9d** have also shown a similar pattern, and their extending protons and carbons were found in the range of 2.04–1.34 ppm in ^1^H NMR spectra and 80.00–20.00 ppm in ^13^C NMR spectra, respectively. The 3-chloro-4-(3-(trifluoromethyl)phenoxy)aniline derivative **9e** was identified by one proton of benzene ring at 8.22 ppm as a doublet peak with a *J* coupling constant of 4.0 Hz. In addition, there is a singlet peak at 3.95 ppm attributable to the three protons of the methoxy group in the ^1^H NMR spectrum, while its carbon was acquired at 56.35 ppm in the ^13^C NMR spectrum. These findings provided evidence proving the successful synthesis of the desired TAK-285 derivatives **9a**–**h**.

## Biological evaluation

### Kinase assay of TAK-285 derivatives 9a–h over EGFR and HER2

All the newly synthesised TAK-285 derivatives (**9a**–**h**) were assessed over EGFR and HER2 kinases at Reaction Biology Co. (Malvern, PA) via “HotSpot^SM^” assay at 10 µM concentration in the presence of 10 µM of ATP. The results were obtained as the % remaining kinase activity of test samples in comparison to the DMSO vehicle. The rates of % kinase inhibition of compounds **9a**–**h** over both kinases were computed and described in Table 2. In brief, the results revealed promising inhibitory activities of all TAK-285 derivatives **9a**–**h** over EGFR kinase. Incorporation of 3-chloro-4-(3,4-dichlorophenoxy)anilino group in compounds **9a**–**d** demonstrated an inhibitory range of 88.90–94.25%, which is lower than the inhibitory range expressed by derivatives with the hydrophobic moiety of TAK-285 (3-chloro-4-(3-(trifluoromethyl)phenoxy)aniline, **9e**–**h**) that demonstrated an outstanding range of % inhibition over EGFR (96.75–99.33%). In the case of HER2, it was observed that most tested compounds showed lower inhibition rates. Nevertheless, a comparable SAR model was revealed over HER2 kinase. While derivatives **9a**–**d** exhibited a modest inhibitory range of 69.83–80.26%, TAK-285 derivatives **9e**–**h** revealed a higher inhibitory activity range (81.74–97.95%). Among all, compound **9f** exhibited the best dual inhibition values over EGFR and HER2 with 99.33 and 97.95% inhibition over both kinases, respectively. Accordingly, compound **9f** was selected for further investigations.

**Table 2.** % Inhibition results of EGFR and HER2 kinases by compounds **9a**–**h** at 10 µM.

Cpd	% kinase inhibition	
	EGFR	HER2
**9a**	89.03	69.83
**9b**	94.25	80.26
**9c**	89.98	78.59
**9d**	88.90	72.13
**9e**	98.94	81.74
**9f**	99.33	97.95
**9g**	99.19	96.18
**9h**	96.75	96.96

### Dose-dependent assessment of TAK-285 derivative 9f over EGFR and HER2

The primary results over the molecular level of both tyrosine receptors (EGFR and HER2) encouraged us to do further assessment of compound **9f**. A dose-dependent evaluation was carried out to assess its IC_50_ values over both kinases (Table 3). The IC_50_ values of compound **9f** were found to be 2.3 nM over EGFR and 234 nM over HER2. Compared to staurosporine and TAK-285, compound **9f** showed 38- and 10-fold of potency over EGFR, respectively.

**Table 3.** IC_50_ values (µM) of compound **9f**, staurosporine, and TAK-285 over EGFR and HER2 tyrosine kinases.

Cpd	IC_50_ (nM)	
	EGFR	HER2
**9f**	2.3	234
**Staurosporine**	88.1	35.5
**TAK-285** [25]	23	17

### Kinase selectivity assessment of TAK-285 derivative 9f

To assess the selectivity and the kinase inhibition profile of the most active dual inhibitor of this new series (**9f**), an *in vitro* screening assay was performed over a small panel of cancer-related kinases including fibroblast growth factor receptor 1 (FGFR1), vascular endothelial growth factor receptor 2 (VEGFR2), cyclin-dependent kinase 2 (CDK2), c-mesenchymal-epithelial transition factor (c-MET), and p38α mitogen-activated protein kinase (MAPK14) in a single-dose concentration of 10 µM. As shown in Table 4, modest inhibitory activities were detected over the tested enzymes. These findings clearly showed a good selectivity of compound **9f** with its nanomolar potency against both kinases (EGFR and HER2) compared to the other tested kinase targets.

**Table 4.** % Inhibition of TAK-285 derivative **9f** over a small kinase panel at 10 µM.

Cpd	% kinase inhibition (relative to DMSO control)				
	CDK2	c-MET	FGFR1	VEGFR2	MAPK14
**9f**	0.80	24.62	5.54	–4.46	24.31

### *In vitro* cytotoxic activity against PC3 and 22RV1 cells

Some inhibitors of the ErbB (EGFR/HER2/ErbB3/ErbB4) family, notably the dual EGFR/HER2 inhibitor lapatinib, failed in phase II clinical trials despite overexpression of this family in castration-resistant prostate cancer (CRPC)^26^^,^^27^. Accordingly, we aimed to investigate the cytotoxic potential of this new series against prostate carcinoma cell lines. The *in vitro* cytotoxicity of the newly synthesised TAK-285 derivatives (**9a**–**h**) was measured using MTT assay against two human prostate carcinoma cell lines (PC3 and 22RV1). Table 5 indicates that compounds **9a**–**h** showed IC_50_ values in the range of 1.0–7.3 nM and 0.8–2.8 nM against PC3 and 22RV1, respectively. Compound **9f** was the most potent derivative over PC3 cell line with an IC_50_ value of 1.0 nM, while compound **9e** showed the best IC_50_ value over 22 RV1 cell line (0.8 nM). The potent nanomolar cell-based activity of this series of compounds over the tested prostate carcinoma cell line could be attributed to other possible targets in addition to EGFR and HER2 receptors. The presence of the 2-nitroimidazole moiety in the chemical structure of compounds **9a**–**h** could develop possible covalent bonds between the nitroimidazole moiety and some cellular proteins^28^.

**Table 5.** The cytotoxic effect and IC_50_ value assessment of TAK-285 derivatives **9a**–**h** against PC3 and 22RV1 prostate cancer cell lines by MTT assay.

Cpd	IC_50_ value (nM)	
	PC3	22RV1
**9a**	6.1	2.3
**9b**	3.1	2.0
**9c**	3.3	1.9
**9d**	2.1	2.8
**9e**	7.3	0.8
**9f**	1.0	2.7
**9g**	4.6	1.3
**9h**	5.6	1.1

Three independent experiments were performed in triplicate. Standard deviation (±SD) of the mean was also obtained for all experiments along with the IC_50_ values.

### Cell cycle analysis

**Figure 3.**
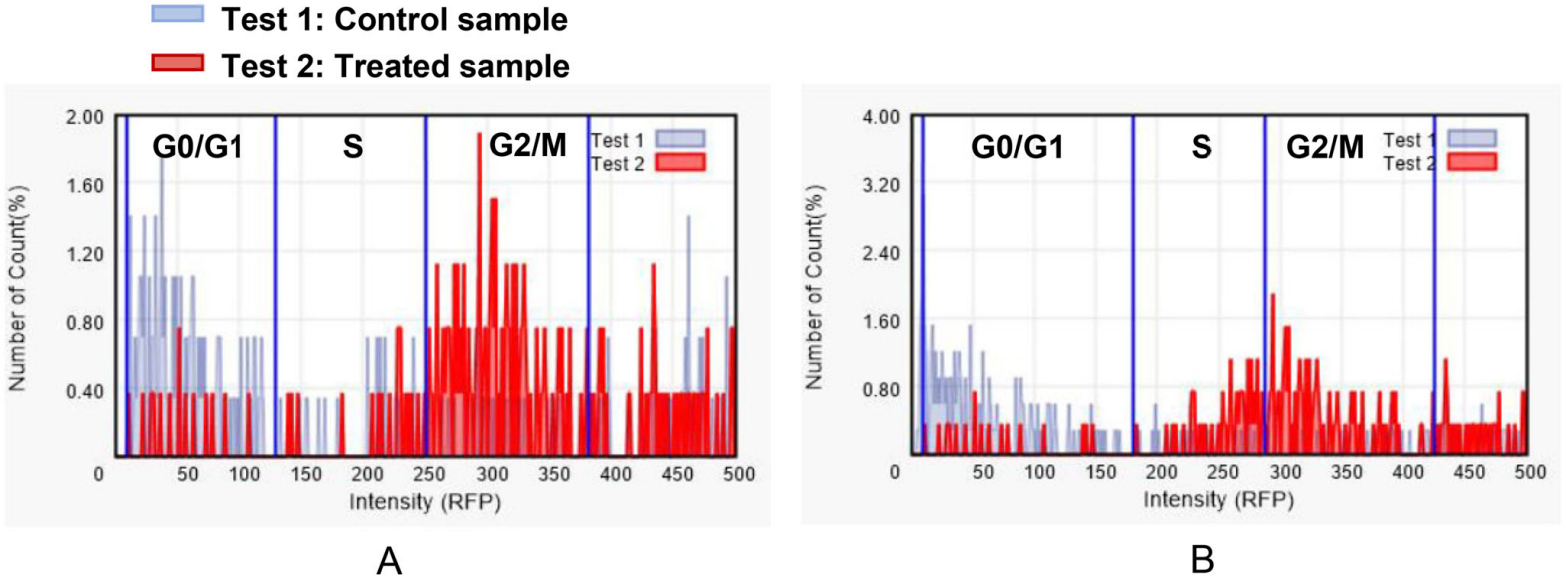
The effect of compound **9f** (test 2) on the phases of the cell cycle compared with control (test 1); 22RV1 cells (A) and PC3 (B).

Cell cycle progression is responsible for normal cell growth and proliferation. DNA damage can result in apoptosis, which causes cell death, or DNA repair. At specific checkpoints that serve as control mechanisms to guarantee correct cell division, the state of the cells is evaluated. Checkpoints in the cell cycle include the G1 (restriction), S (metaphase), and G2/M^29^. Anticancer medications’ function is to halt cell division at these checkpoints. Treatment with potent cytotoxic (as an anticancer) agents can determine at which phase apoptosis occurs in the cell cycle. As a result, the most potent derivative, **9f**, was chosen for testing its outcomes on the cell cycle profile and apoptosis. PC3 and 22RV1 cells were treated with compound **9f** at its IC_50_. The comparison data in Table 6 and Figure 3 indicate that compound **9f** (test 2) arrested the cell cycle of 22RV1 and PC3 cells at the G2/M phase by 62.74% and 49.43%, respectively (Figure 3). Also, the cell population in G1 and S phases decreases after treatment (test 2) compared to a negative control (test 1). The comparison data showed the control sample has arrested the cell cycle at G0/G1 phases while **9f** treated sample has arrested it at G2/M phases as indicated by higher number of counts (%) in these phases of both cell cycle studies.

**Table 6.** The effect of compound **9f** on the different phases of cell cycle of PC3 and 22RV1 cell lines.

	22RV1				PC3			
	9f		Control		9f		Control	
	Conc. (cells/mL)	Percent	Conc. (cells/mL)	Percent	Conc. (cells/mL)	Percent	Conc. (cells/mL)	Percent
G0/G1 phase	2.11 × 10E3	6.08%	1.12 × 10E5	60.24%	2.50 × 10E3	7.22%	3.65 × 10E4	65.72%
S phase	3.16 × 10E3	9.13%	3.27 × 10E4	17.65%	9.73 × 10E4	28.14%	6.84 × 10E3	12.29%
G2/M phase	2.17 × 10E4	62.74%	2.83 × 10E4	15.24%	1.71 × 10E4	49.43%	7.36 × 10E3	13.24%

### Apoptosis analysis

**Figure 4.**
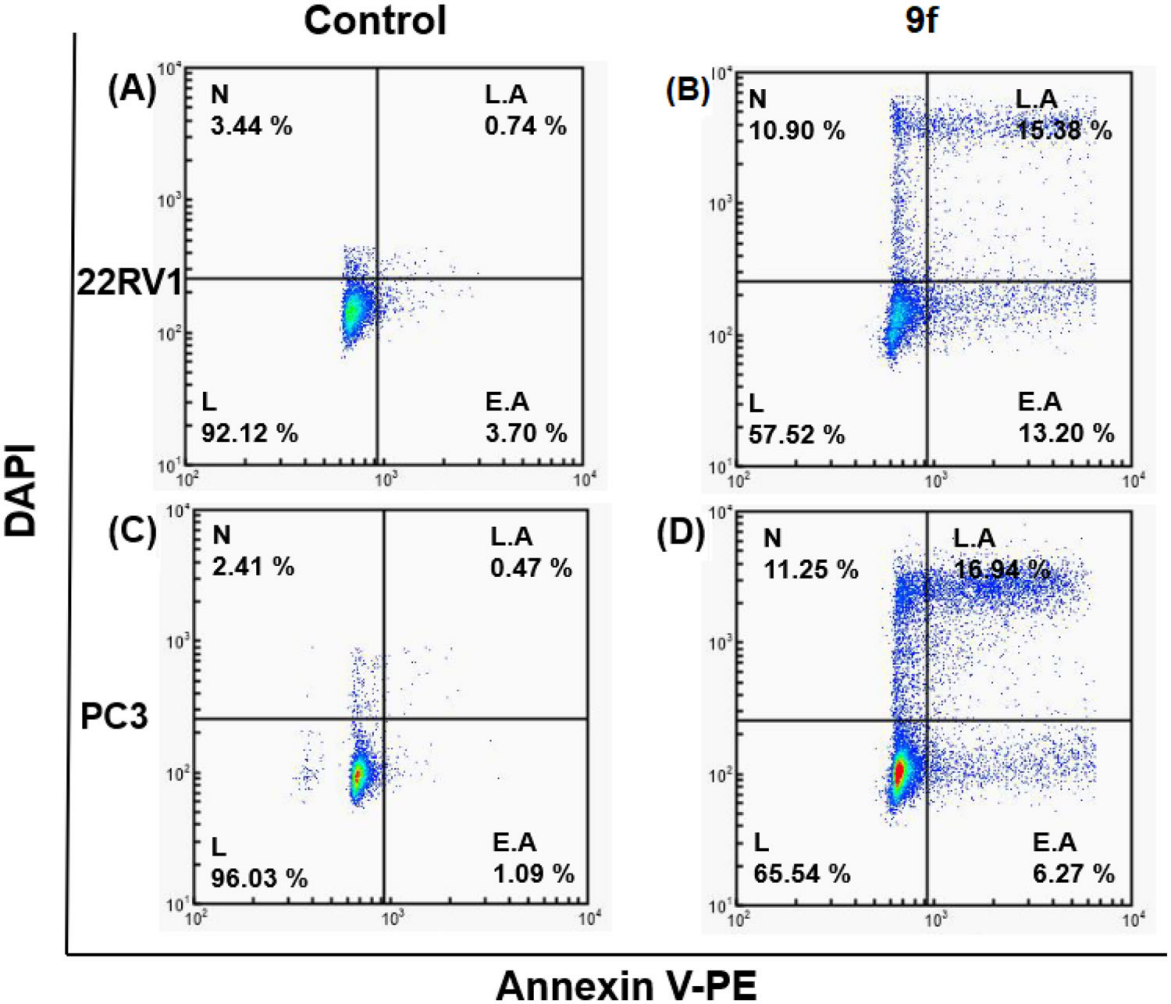
Dot plots showing apoptosis analysis of 22RV1 cells (A, B) and PC3 (C, D) induced by compound **9f** along its control.

**Figure 5.**
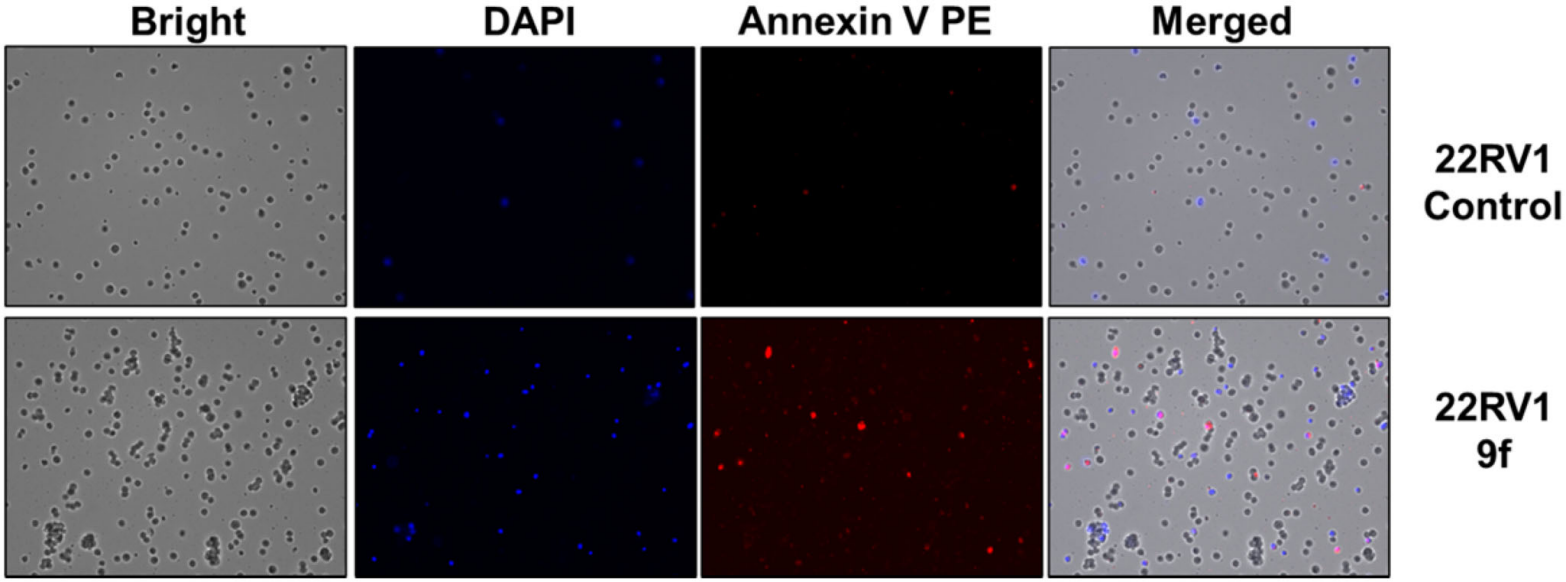
Cellular fluorescence images of 22RV1 cell line treated with compound **9f** for 24 h. Bright-field images, fluorescence images (DAPI: 4′,6-diamidino-2-phenylindole, Annexin V PE), and merged images were assigned to the 22RV1 prostate cancer cells with control (without any compound treatment), compound **9f**-treated, showing apoptotic cells.

**Figure 6.**
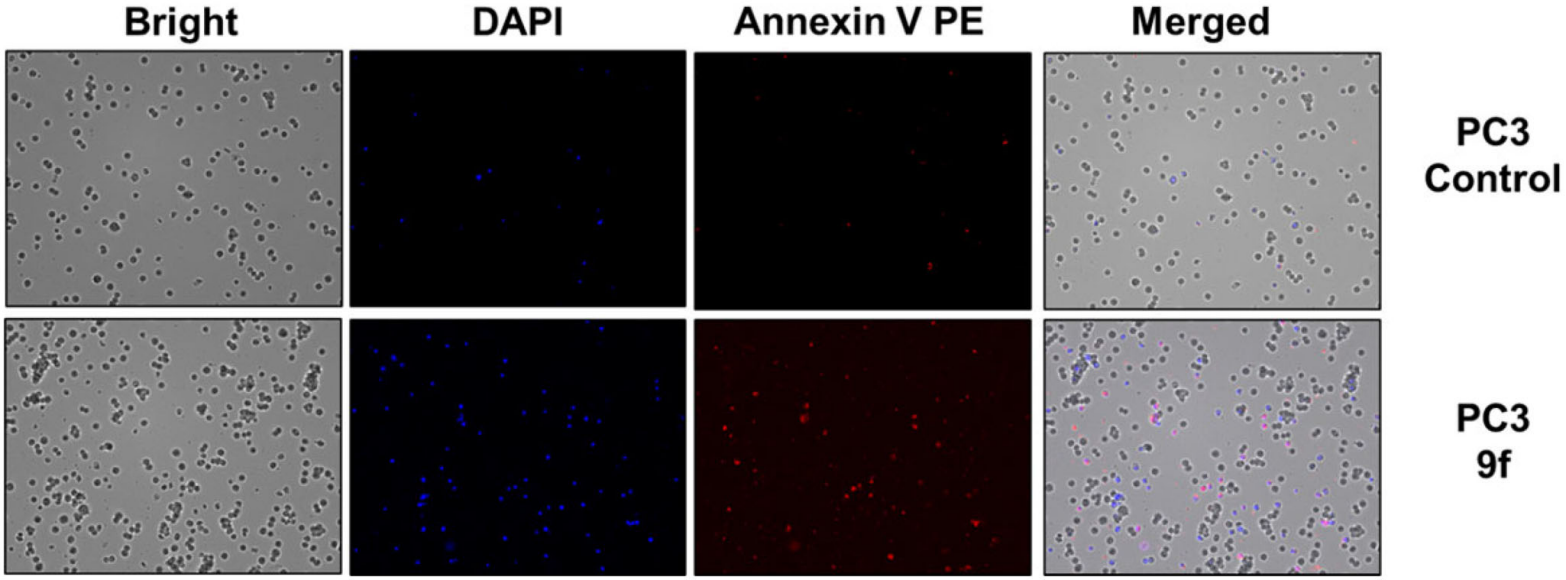
Cellular fluorescence images of PC3 cell line treated with compound **9f** for 24 h. Bright-field images, fluorescence images (DAPI: 4′,6-diamidino-2-phenylindole, Annexin V PE), and merged images were assigned to the PC3 prostate cancer cells with control (without any compound treatment), compound **9f**-treated, showing apoptotic cells.

The control PC3 and 22RV1 cells and compound-treated cells were stained with PE-Annexin V and DAPI for a more appropriate cell death examination, and the cellular fluorescence analysis was then performed using ADAMII LS (Figures 4–6). Apoptosis is a type of programmed cell death that can be detected using Annexin V and the DAPI reagent. On plasma membranes, annexin V binds to phosphatidyl amine and DAPI can bind to DNA in cells. The dot plot and image data detect early and late apoptotic cells using these two fluorophores. The dot plot results revealed that a large portion of compound **9f**-treated 22RV1 cells underwent early apoptosis (13.20%), and simultaneously, some cells had prominently progressed to the late apoptosis phase (15.38%) (Figure 4(B)). We found that most of the cancer cells in the control groups (about 92.12% and 96.03%, respectively) were alive (Figures 4(A,C), 5 and 6). Moreover, compound **9f**-treated PC3 cells went through early apoptosis (6.27%), and late apoptosis phase (16.94%) (Figures 4(D) and 6). Likewise, the fluorescence images for 22RV1 (Figure 5) and PC3 (Figure 6) cells complement the dot plot data, as higher fluorescence of DAPI and RF images clearly distinguished compound **9f**-treated cells from control. Therefore, compound **9f** exerted noticeable damage to 22RV1 and PC3 cells when treated with compound doses around its IC_50_ value. The results of the cell cycle and apoptosis studies suggest that compound **9f** has remarkable anticancer properties for chemotherapy.

Annexin V conjugated to fluorochromes (PE) retains its high affinity for phosphatidylserine (PS), making it a sensitive probe for flow cytometric analysis of apoptotic cells. PE Annexin V staining occurs prior to the loss of membrane integrity that occurs in the final stages of cell death caused by either apoptotic or necrotic processes. As a result, PE Annexin V staining is typically used in conjunction with a vital dye such as propidium iodide (PI) or DAPI to allow the investigator to identify early apoptotic cells (DAPI negative, PE Annexin V positive). Viable cells with intact membranes exclude DAPI, whereas dead and damaged cells’ membranes are permeable to DAPI. When apoptosis is measured over time, cells can often be tracked from PE Annexin V and DAPI negative (viable, or no measurable apoptosis), to PE Annexin V positive and DAPI negative (early apoptosis), and finally to PE Annexin V and DAPI positive (end stage apoptosis and death). The progression of cells through these three stages suggests apoptosis.

### Molecular docking

To validate the dual inhibitory activities of the newly designed TAK-285 derivatives (**9a**–**h**) towards EGFR (PDB ID: 1M17^30^) and HER2 (PDB ID: 3RCD^25^) receptors, molecular docking studies were carried out using the MOE 2019.0102^31^^,^^32^. Besides, the co-crystallised inhibitors (4-anilinoquinazoline (AQ4) and pyrrolo[3,2-*d*]pyrimidine (03P)) of EGFR and HER2 were inserted as reference standards. Since all docked compounds (**9a**–**h**) showed promising results, derivatives **9f** and **9g**, which were biologically superior, were selected for deep investigations compared to the co-crystallised inhibitor in each case.

The binding pocket of EGFR receptor (PDB ID: 1M17^30^) showed that Met769, Gln767, Cys773, Cys751, Lys692, Thr766, and Thr830 are very crucial to produce the antagonistic activity. The docked AQ4 inhibitor bound Cys773 through a pi–H bond (4.68 Å) with a binding score of −7.47 kcal/mol (RMSD = 1.01). However, compound **9f** showed the formation of one H-bond with Lys692 (2.95 Å) and three pi–H bonds with Cys773, Val702, and Arg817 (4.55, 4.33, and 4.65 Å, respectively). Its binding score was found to be −8.79 kcal/mol (RMSD = 1.13), besides compound **9g** binding score was −8.46 kcal/mol (RMSD = 1.45), indicating superior binding affinities for both derivatives compared to that of the docked AQ4 inhibitor. Also, compound **9g** formed one H-bond with Met769 (3.16 Å) and two pi–H interactions with Cys773 and Leu694 (4.33 and 4.61 Å, respectively), as depicted in Table 7.

**Table 7.** 3D binding interactions and positioning of the docked co-crystallised AQ4 inhibitor, **9f**, and **9g** candidates within the EGFR (PDB ID: 1M17) binding pocket.

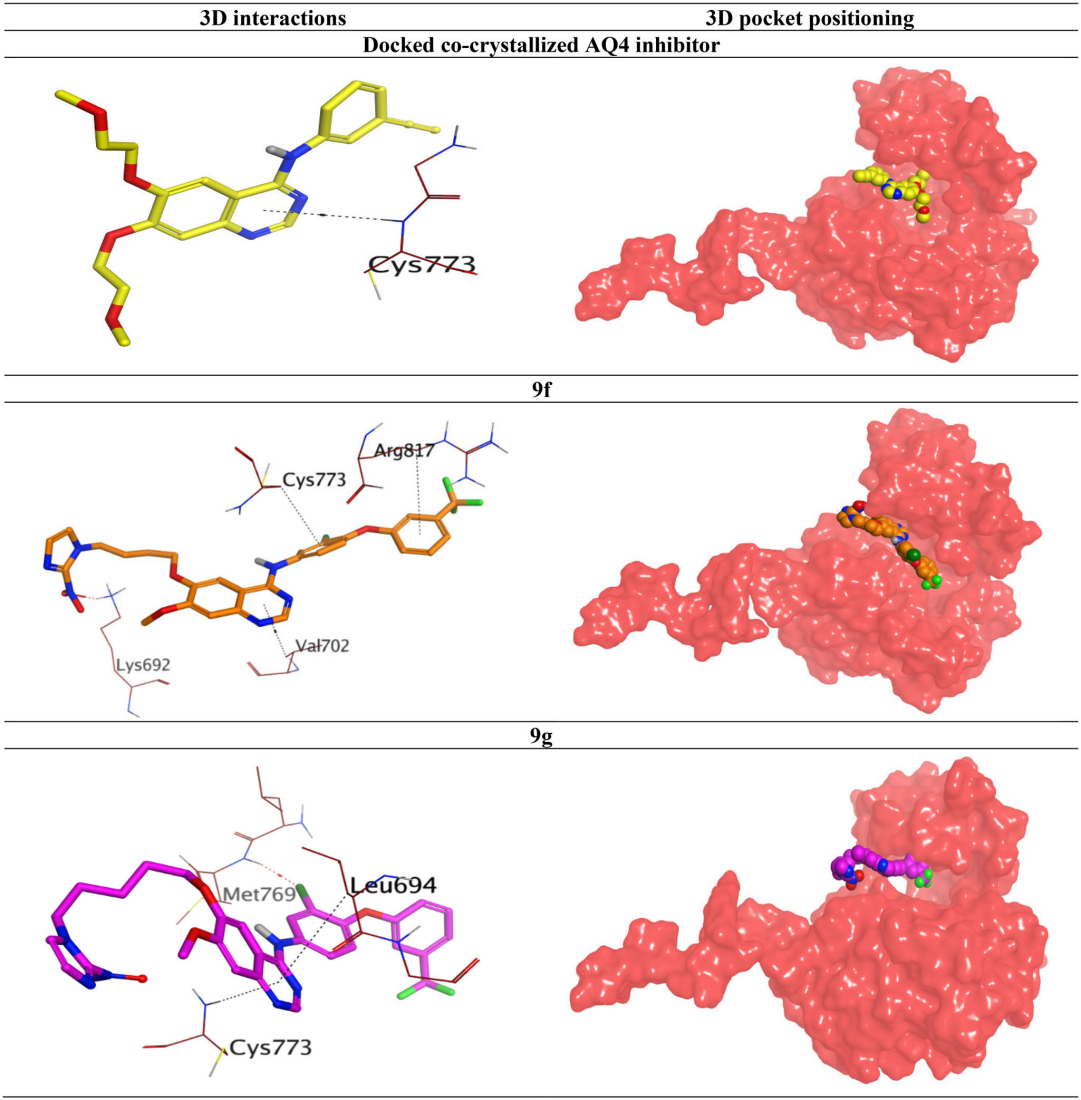	

On the other hand, the binding site of the HER2 receptor (PDB ID: 3RCD^25^) clarified that Met801, Leu726, and Lys753 represent the most important amino acids to produce the antagonistic activity. Herein, the docked 03P inhibitor formed two H-bonds with Met801 and Lys753 (3.08 and 3.04 Å, respectively) with a binding score of −11.52 kcal/mol (RMSD = 1.76). Furthermore, compound **9f** (*S* = −10.64 kcal/mol and RMSD = 1.74) achieved two H-bonds with Met801 and Cys805 (3.43 and 3.70 Å, respectively). Besides, compound **9g** (*S* = −10.26 kcal/mol and RMSD = 1.94) got stabilised through the formation of four H-bonds with Met801 (1), Lys753 (2), and Phe731 (1) at 3.52, 3.01, 3.20, and 3.39 Å, respectively, as represented in Table 8. Based on the above fact, we can conclude the very promising inhibitory activities of the newly designed candidates (especially **9f** and **9g** members) towards the binding pockets of both EGFR and HER2 receptors.

**Table 8.** 3D binding interactions and positioning of the docked co-crystallised 03P inhibitor, **9f**, and **9g** candidates within the HER2 (PDB ID: 3RCD) binding pocket.

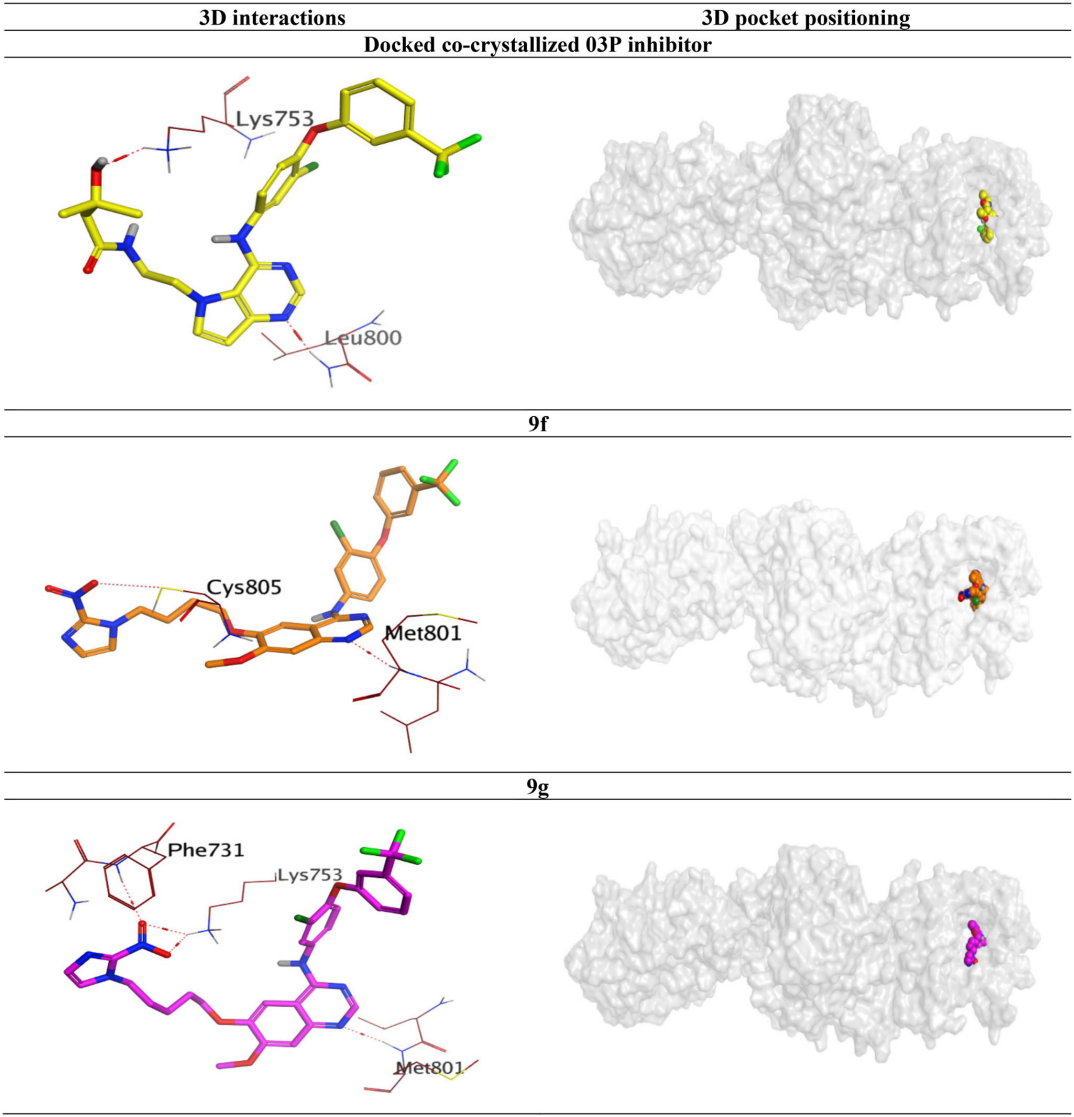	

### Molecular dynamics (MD) simulations

**Figure 7.**
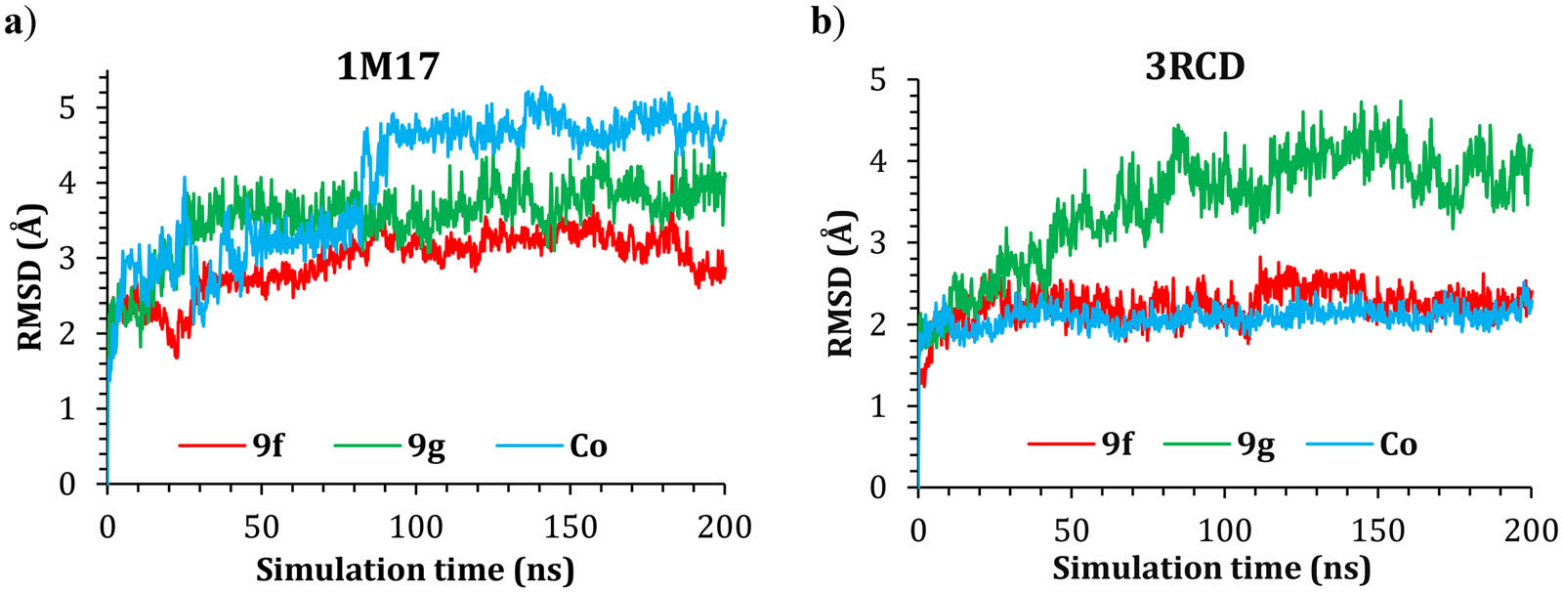
The RMSD of **9f**, **9g**, and co-crystal ligand in complex with (a) 1M17 and (b) 3RCD.

Molecular dynamic simulations were applied to simulate the behaviour of the hit compounds in a cell-like environment. Compounds **9f** and **9g** were selected, and their performance was studied inside the active site of both the EGFR (1M17) and HER2 (3RCD) tyrosine kinase domains. The protein conformational change was monitored via the difference in the position of the Cα atoms of the protein backbone and was reported in Å. The positional change of the Cα for both protein complexes was plotted as a function of simulation time in Figure 7.

**Figure 8.**
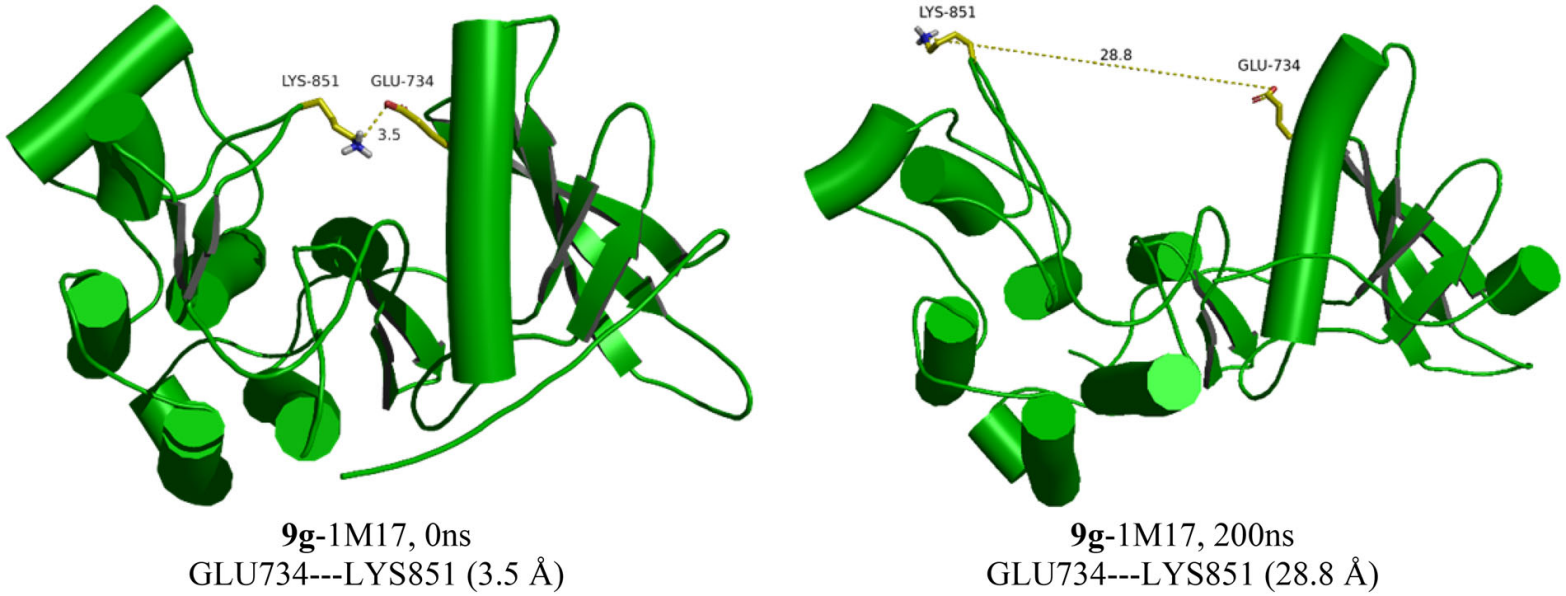
The GLU734–LYS851 distances in the **9g**-1M17 complex during the simulations time.

The RMSD of the 1M17 complexes is plotted in Figure 7(a), and as seen from the figure, the **9f**-1M17 complex showed an RMSD of about 3.00 Å, which is considered acceptable for such protein. The **9g**-1M17 and the Co-1M17 complexes showed a fluctuation at around 4.00 and 5.00 Å, respectively; such instability is due to the presence of the hinge as it is playing a critical rule whenever the ligand tries to orient itself inside the active site, the salt bridge between the residue Glu734 and Lys851 will break, the protein subunits will move apart, and the hinge will open which results in higher fluctuation. The distance between the Glu734 and Lys851 for the **9g**-1M17 complex is depicted in Figure 8, and for **9f**, the co-crystal is illustrated in Figure SI 1 (SI).

**Figure 9.**
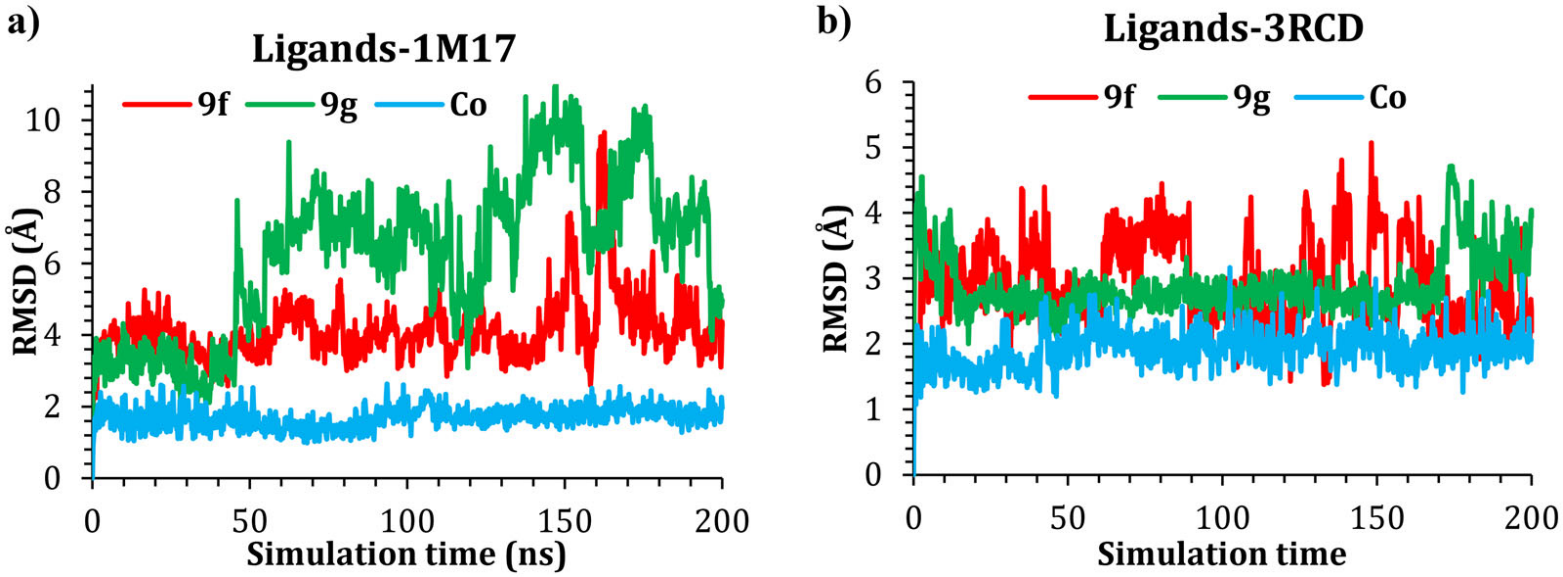
Plots of RMSD for ligand atoms (Å) with respect to the initial structure vs. simulation time (ns) for all the complexes.

In the case of the 3RCD complexes, the **9f**-3RCD and the Co-3RCD were stable and fluctuated at around 2.00 Å. On the other hand, the **9g**-3RCD complex fluctuates at around 4.00 Å, which comes from the unfolded loop of the Arg756, Glu757, Asn758, Thr759, and Ser760, coloured red in Figure 9. On the other hand, the ligand’s behaviour was also monitored via the RMSD with respect to their initial position, and the RMSD plotted as a function of simulation time for ligand–1M17 complexes in Figure 9(a) and ligand–3RCD complexes in Figure 9(b).

**Figure 10.**
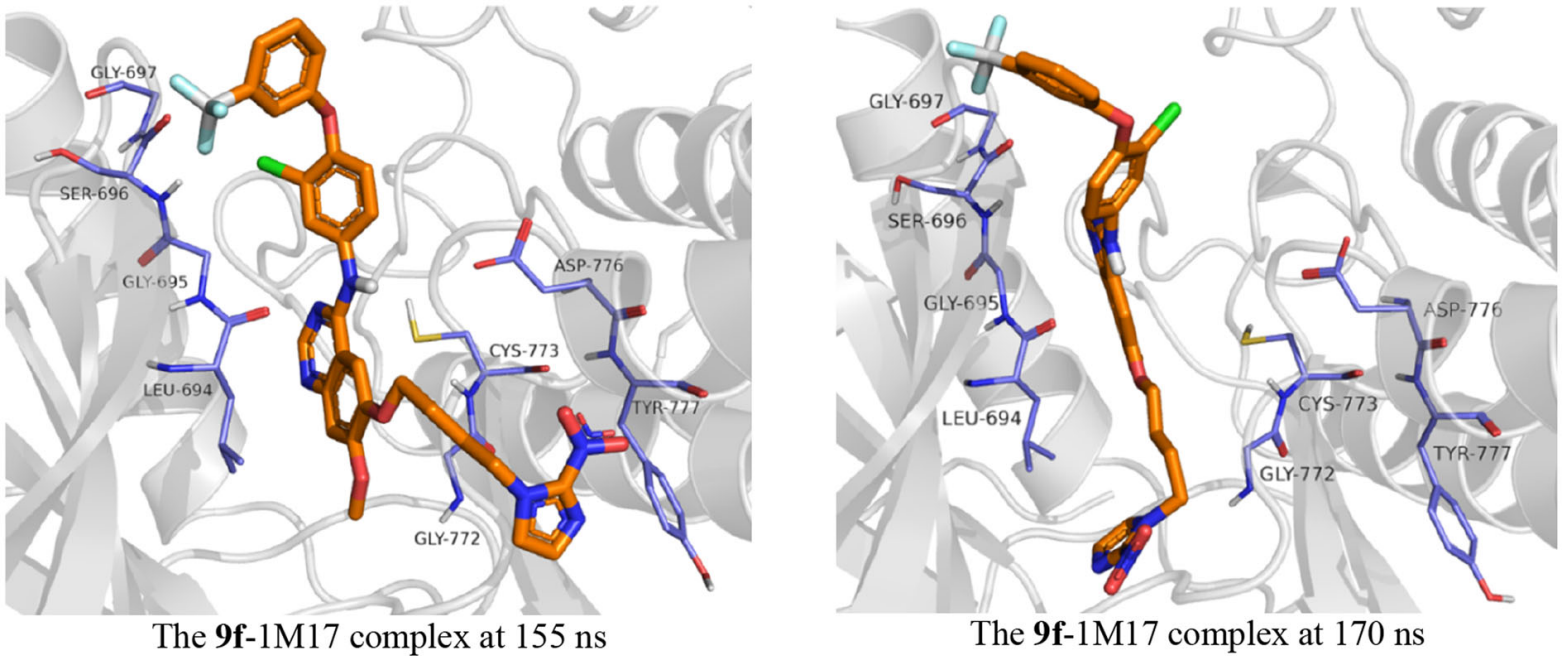
Snapshot of **9f**-1M17 at 155 and 170 ns of simulation time showing the moving of the imidazole group out of the active site at 155 ns.

With regards to 1M17, compound **9g** was unstable inside the active site. This instability is a result of the moving of the imidazole ring out of the active site, and this leads to relocating the ligand within the active site. The butyl bridge rotatability plays a critical role in this instability. Compound **9f**, on the other hand, showed much more stability; the compound was stable during the simulation time, at around 155–170 ns, a fluctuation of 8.00 Å was observed; this fluctuation is again due to the presence of the alkyl bridge, the imidazole group moved out of the protein active site during this period, before it goes back inside the active site as described in Figure 10. The co-crystal ligand showed stability inside the active site with an RMDS of almost 2.00 Å.

In the case of 3RCD, compound **9f** showed a fluctuation between 2.00 and 4.00 Å; the compound imidazole group was shifting its position inside the active site, which led to this RMSD. Compound **9g** also showed similar behaviour, and the pentyl bridge gives the imidazole flexibility to move out/in the active site, which results in an RMSD of 3.00–4.00 Å. The co-crystal ligand holds tight inside the active site with an RMSD of ∼2.00 Å during the simulation.

Further, a deep analysis of the interactions of the compounds **9f**-1M17 and **9g**-3RCD with the active site residues was carried out. The interactions of these ligands with protein residue were plotted using the simulation interaction diagram panel of Maestro software.

**Figure 11.**
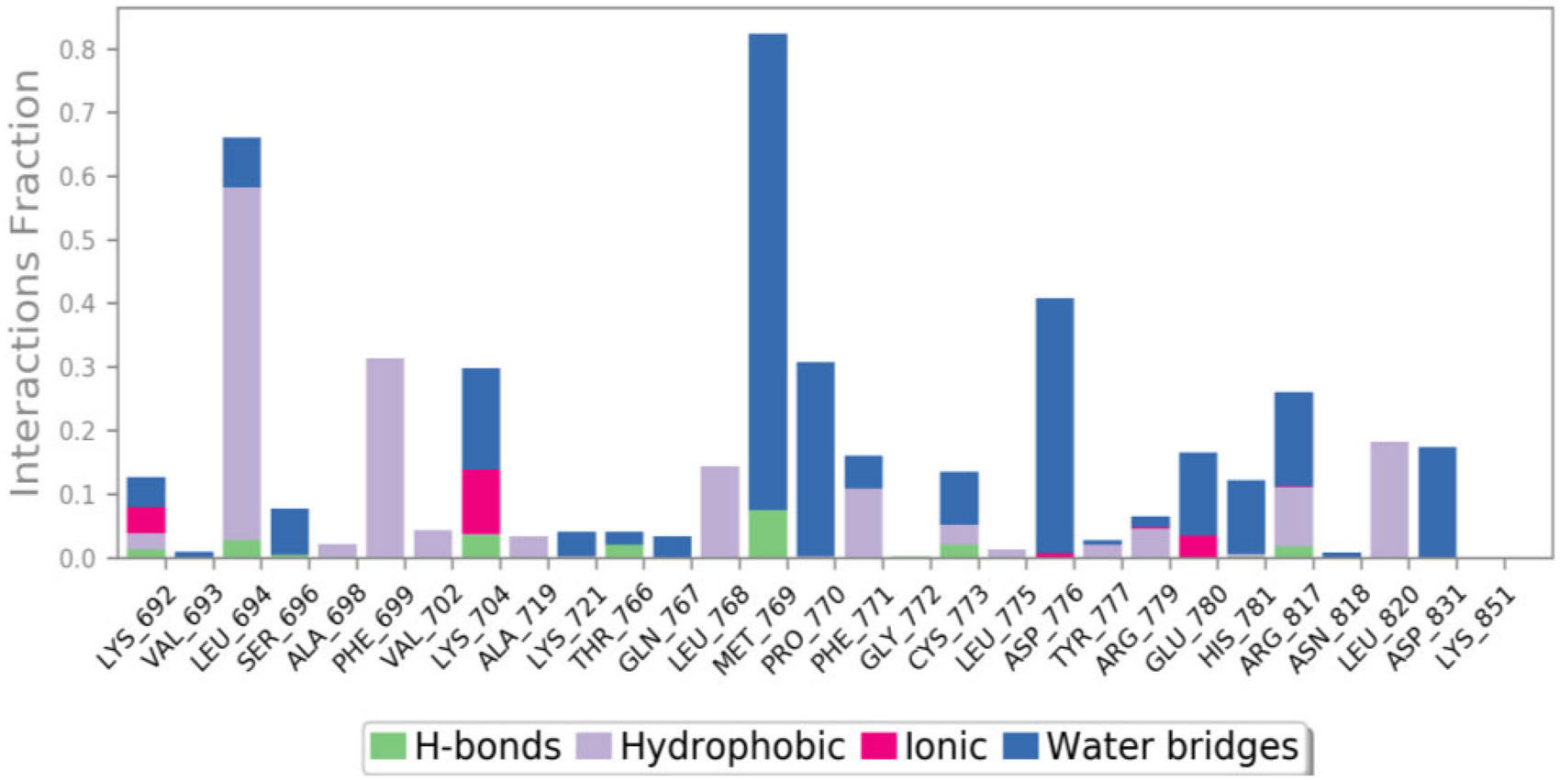
The histogram of **9f**-1M17 contact throughout the trajectory.

**Figure 12.**
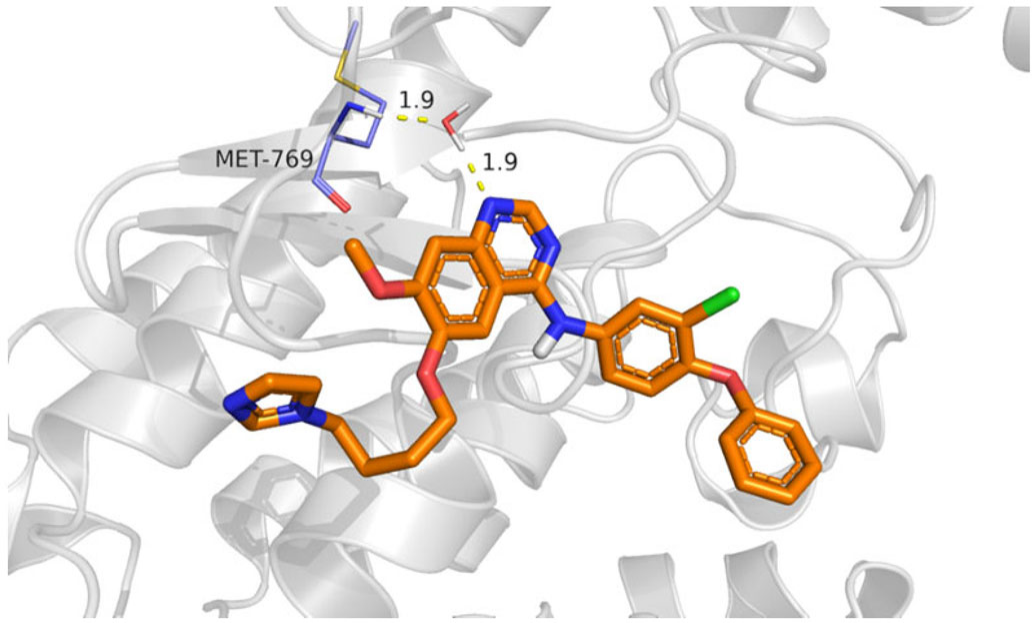
Compound **9f** water bridge H-bond interactions with Met769 residue in 1M17.

The active site cavity of the 1M17 is quite big, and hence, the stability of ligands inside the active side is dependent on water bridge H-bonding, especially to residue Met769, as observed in the case of the reference compound, Figure SI 3. Compound **9f** interactions histogram (Figures 11 and 12) shows that multiple water bridge H-bonds were formed, including residue Met769 (70%), Asp776 (40%), and Pro770 (30%). In addition, residues Leu694 (60%) and Phe699 (32%) were able to form lipophilic interactions with **9f** during the simulation time. The residues that were able to develop interactions more than 30% of the simulation time are reported in Figure SI 4.

**Figure 13.**
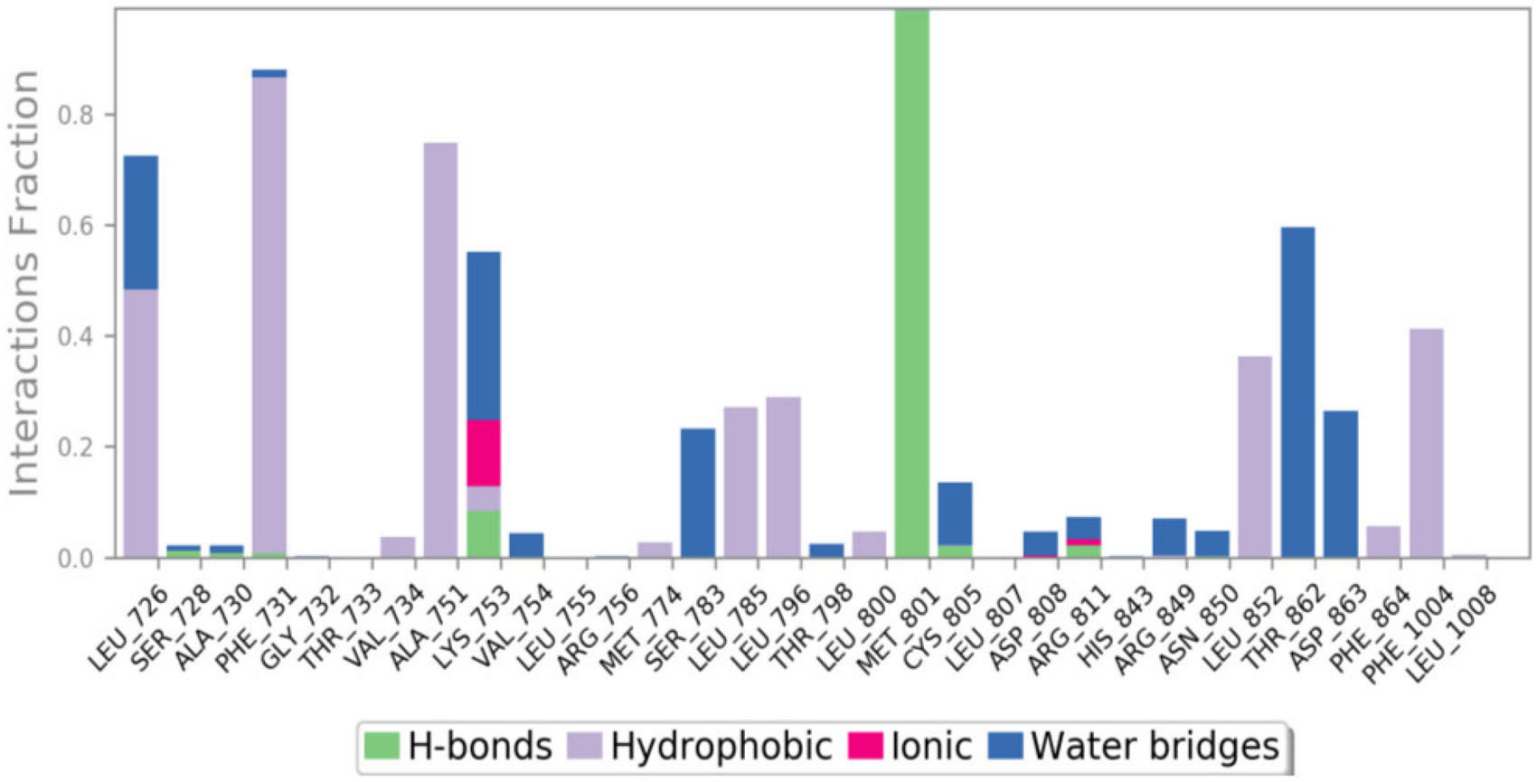
The histogram of **9g**-3RCD contact throughout the trajectory.

**Figure 14.**
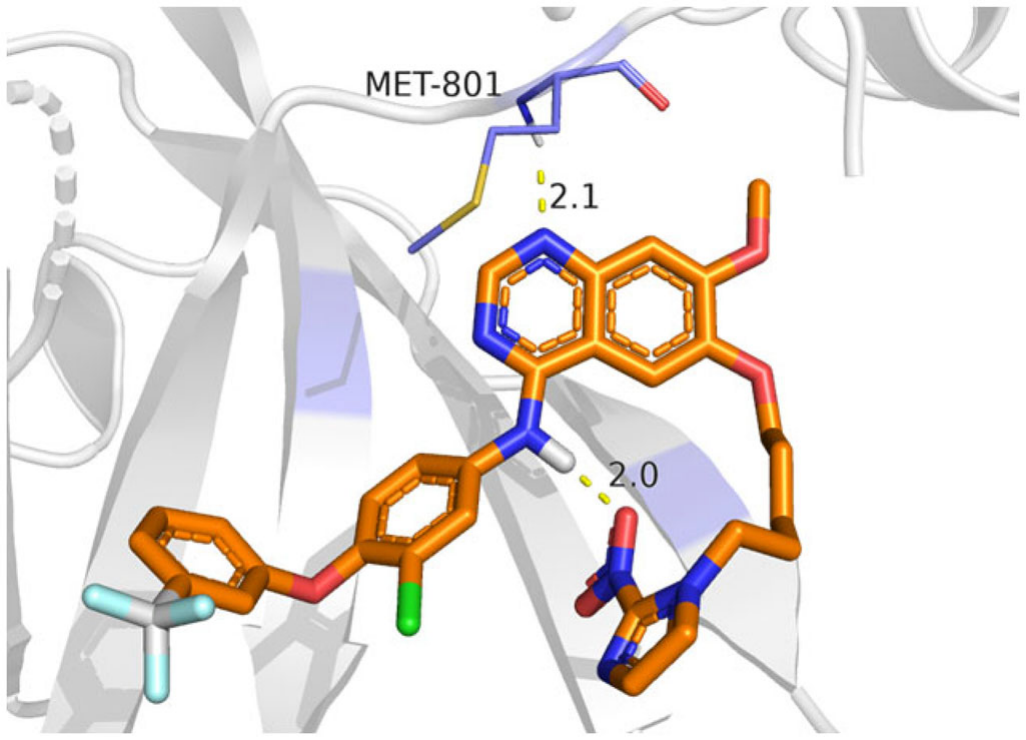
Compound **9g** H-bond interactions with 3RCD residues.

In the case of 3RCD, compound **9g** showed much more stability inside the active site of the 3RCD, as seen in the interaction histogram (Figure 13), the Met801 residue playing an essential role via forming an H-bond interaction almost 100% of the simulation time (Figure 14). Compound **9g** was able to develop lipophilic interactions with Leu726 (50%), Phe731 (95%), Ala751 (75%), Phe1004 (45%), and Leu852 (40%). In addition, Thr862 formed a water bridge hydrogen bond almost 65% of the simulation time, along with Lys753 (35%). The residues that were able to form interactions more than 30% of the simulation time are reported in Figure SI 5.

### MM-GBSA study

The average MM-GBSA binding energy over the last 50 ns was generated using the thermal_mmgbsa.py python script provided by Schrödinger, which also generates Coulomb energy, covalent binding energy, van der Waals energy, lipophilic energy, generalised Born electrostatic solvation energy, and hydrogen-bonding energy. All the obtained data are shown in Table 9. MM-GBSA free binding energy reveals that compounds **9f** and **9g** showed lower binding energy compared to the co-crystal ligand in the case of 1M17, while in the case of 3RCD, compound **9g** showed superiority over **9f**, which in turn, showed higher binding energy compared to the co-crystal ligand.

**Table 9.** Prime MM-GBSA energies for ligands binding at the active sites of EGFR (1M17) and HER2 (3RCD) receptors.

	Δ*G* binding	Coulomb	Covalent	H-bond	Lipo	Solv_GB	vdW
**9f**-1m17	–51.62	–3.91	3.78	–0.42	–17.18	18.61	–51.15
**9g**-1m17	–53.22	–5.69	3.61	–0.76	–18.76	15.62	–46.07
Co-1M17	–63.27	–15.75	1.91	–0.57	–18.90	25.98	–55.93
**9f**-3RCD	–82.64	–4.64	4.40	–0.67	–26.51	18.71	–73.33
**9g**-3RCD	–98.27	–4.20	3.39	–0.51	–31.52	17.10	–81.00
Co-3RCD	–75.81	–10.36	0.63	–0.84	–21.37	24.94	–68.51

Coulomb: Coulomb energy; covalent: covalent binding energy; vdW: van der Waals energy; Lipo: lipophilic energy; Solv_GB: generalised Born electrostatic solvation energy; H-bond: hydrogen-bonding energy.

## Experimental

### Chemistry

The general protocols utilised for the chemical synthesis, structure elucidation, and purity of the synthesised compounds were provided in the Supplementary File. Compounds **2a**–**d** were synthesised in our recently published report^24^. Compounds **4** and **5** were synthesised following the earlier report^33^, and their ^1^H NMR spectra were added in the Supplementary File.

### Synthesis of compounds 7a and 7b

A round-bottom flask was charged with 4-chloro-7-methoxyquinazolin-6-yl acetate (**6**, 1.0 equiv.) and the appropriate aniline reagent (1.2 equiv.) in isopropyl alcohol (*i*-PrOH). The mixture was refluxed for 4 h and cooled to rt after completion of the reaction. The reaction mixture was filtered using *i*-PrOH to give the target intermediate, which was used in the next step without further purification.

*4-((3-Chloro-4-(3,4-dichlorophenoxy)phenyl)amino)-7-methoxyquinazolin-6-yl acetate (***7a***).* Ivory solid. Yield: 100% (1.99 g, 3.96 mmol). ^1^H NMR (400 MHz, DMSO-*d_6_*) *δ* 11.10 (s, 1H, NH), 8.92 (s, 1H, quinazoline-C2-H), 8.61 (s, 1H, quinazoline-C8-H), 8.13 (d, *J* = 2.4 Hz, 1H, Ar-H), 7.76 (dd, *J* = 8.8, 2.4 Hz, 1H, Ar-H), 7.64 (d, *J* = 8.0 Hz, 1H, Ar-H), 7.43 (s, 1H, quinazoline-C5-H), 7.35 (d, *J* = 8.0 Hz, 1H, Ar-H), 7.31 (d, *J* = 4.0 Hz, 1H, Ar-H), 6.97 (dd, *J* = 8.8, 2.8 Hz, 1H, Ar-H), 3.99 (s, 3H, OCH_3_), and 2.38 (s, 3H, OAc-H).

*4-((3-Chloro-4-(3-(trifluoromethyl)phenoxy)phenyl)amino)-7-methoxyquinazolin-6-yl acetate (***7b***).* Ivory solid. Yield: 100% (1.45 g, 2.89 mmol). ^1^H NMR (400 MHz, DMSO-*d_6_*) *δ* 10.99 (s, 1H, NH), 8.91 (s, 1H, quinazoline-C2-H), 8.57 (s, 1H, quinazoline-C8-H), 8.15 (d, *J* = 2.4 Hz, 1H, Ar-H), 7.78 (dd, *J* = 8.0, 4.0 Hz, 1H, Ar-H), 7.63 (t, *J* = 8.0 Hz, 1H, Ar-H), 7.51 (d, *J* = 8.0 Hz, 1H, Ar-H), 7.41 (s, 1H, quinazoline-C5-H), 7.36 (d, *J* = 8.0 Hz, 1H, Ar-H), 7.29–7.24 (m, 2H, Ar-H), 3.99 (s, 3H, OCH_3_), and 2.38 (s, 3H, OAc-H).

### Synthesis of compounds 8a and 8b

The appropriate acetate intermediate (**7**, 3–4 mmol) and an excess amount of aqueous ammonia solution (28% in H_2_O) in methanol (CH_3_OH) were put into the round-bottom flask and stirred for 4 h at rt. The excess solvent of the resulting mixture was partially concentrated, filtered with chilled methanol, and dried to give the required intermediates without further purification.

*4-((3-Chloro-4-(3,4-dichlorophenoxy)phenyl)amino)-7-methoxyquinazolin-6-ol (***8a***).* Ivory solid. Yield: 94.3% (1.72 g, 3.72 mmol). ^1^H NMR (400 MHz, DMSO-*d_6_*) *δ* 9.70 (s, 1H, OH), 9.52 (s, 1H, NH), 8.48 (s, 1H, quinazoline-C2-H), 8.29 (d, *J* = 4.0 Hz, 1H, Ar-H), 7.91 (dd, *J* = 8.0, 4.0 Hz, 1H, Ar-H), 7.79 (s, 1H, quinazoline-C8-H), 7.60 (d, *J* = 8.0 Hz, 1H, Ar-H), 7.29 (d, *J* = 8.0 Hz, 1H, Ar-H), 7.22–7.21 (m, 2H, quinazoline-C5-H and Ar-H), 6.91 (dd, *J* = 8.0, 4.0 Hz, 1H, Ar-H), and 3.96 (s, 3H, OCH_3_). ^13^C NMR (100 MHz, DMSO-*d*_6_) *δ* 157.25 (quinazoline-C4), 156.21 (quinazoline-C7), 154.41 (Ar-C), 152.35 (quinazoline-C2), 147.25 (quinazoline-C6), 146.73 (quinazoline-C8a), 145.12 (Ar-C), 138.83 (Ar-C), 132.46 (Ar-C), 132.00 (Ar-C), 125.27 (Ar-C), 125.23 (Ar-C), 123.10 (Ar-C), 122.95 (Ar-C), 121.96 (Ar-C), 118.70 (Ar-C), 117.05 (Ar-C), 110.10 (quinazoline-C4a), 107.65 (quinazoline-C8), 105.74 (quinazoline-C5), and 56.40 (OCH_3_). HRMS (ESI) *m/z* calculated for C_21_H_15_Cl_3_N_3_O_3_ [M + H]^+^: 462.0179, found: 462.0170.

*4-((3-Chloro-4-(3-(trifluoromethyl)phenoxy)phenyl)amino)-7-methoxyquinazolin-6-ol (***8b***).* Ivory solid. Yield: 64% (0.85 g, 1.85 mmol). ^1^H NMR (400 MHz, DMSO-*d_6_*) *δ* 9.71 (s, 1H, OH), 9.53 (s, 1H, NH), 8.49 (s, 1H, quinazoline-C2-H), 8.30 (d, *J* = 2.4 Hz, 1H, Ar-H), 7.92 (dd, *J* = 8.0, 4.0 Hz, 1H, Ar-H), 7.79 (s, 1H, quinazoline-C8-H), 7.60 (t, *J* = 8.0 Hz, 1H, Ar-H), 7.45 (d, *J* = 8.0 Hz, 1H, Ar-H), 7.30 (d, *J* = 8.0 Hz, 1H, Ar-H), 7.21–7.18 (m, 3H, quinazoline-C_5_ and Ar-H), 3.96 (s, 3H, OCH_3_). ^13^C NMR (100 MHz, DMSO-*d*_6_) *δ* 158.23 (quinazoline-C4), 156.23 (quinazoline-C7), 154.41 (Ar-C), 152.36 (quinazoline-C2), 147.25 (quinazoline-C6), 146.73 (quinazoline-C8a), 145.11 (Ar-C), 138.79 (Ar-C), 131.85 (Ar-C), 131.28 (Ar-C), 125.43 (Ar-C), 123.14 (Ar-C), 123.08 (Ar-C), 122.81 (Ar-C), 122.04 (Ar-C), 120.49 (Ar-C), 119.76 (Ar-C), 113.11 (Ar-C), 110.10 (quinazoline-C4a), 107.65 (quinazoline-C8), 105.75 (quinazoline-C5), and 56.39 (OCH_3_). HRMS (ESI) *m/z* calculated for C_22_H_16_ClF_3_N_3_O_3_ [M + H]^+^: 462.0832, found: 462.0821.

### Synthesis of the final TAK-285 derivatives 9a–h

To the intermediate (**8a**, 1.0 equiv.) in DMF, potassium carbonate (2.0 equiv.) and the appropriate bromoalkyl imidazole (**2a**–**d**, 1.2 equiv.) were added at rt. The reaction mixture was stirred for 4 h at 80 °C and partitioned using EtOAc and water. The collected organic layer was dried over MgSO_4_, filtered, and concentrated *in vacuo*. The crude was purified by flash column chromatography (CH_2_Cl_2_:CH_3_OH = 20:1) to afford the desired final TAK-285 derivatives.

*N-(3-Chloro-4-(3,4-dichlorophenoxy)phenyl)-7-methoxy-6-(3-(2-nitro-1H-imidazol-1-yl)propoxy)quinazolin-4-amine (***9a***).* Yellow solid (101.9 mg, 0.17 mmol). m.p.: 237–238 °C. HPLC purity: 96.95% (retention time, RT = 14.885 min). ^1^H NMR (400 MHz, DMSO-*d_6_*) *δ* 9.55 (s, 1H, NH), 8.52 (s, 1H, quinazoline-C2-H), 8.18 (d, *J* = 2.8 Hz, 1H, Ar-H), 7.85 (dd, *J* = 8.8, 2.4 Hz, 1H, Ar-H), 7.81 (s, 1H, quinazoline-C8-H), 7.67 (d, *J* = 0.8 Hz, 1H, imidazole-C5-H), 7.62 (d, *J* = 12.0 Hz, 1H, Ar-H), 7.32 (d, *J* = 8.0 Hz, 1H, Ar-H), 7.23 (d, *J* = 2.8 Hz, 1H, imidazole-C4-H), 7.21 (s, 1H, quinazoline-C5-H), 7.17 (d, *J* = 0.8 Hz, 1H, Ar-H), 6.93 (dd, *J* = 8.0, 4.0 Hz, 1H, Ar-H), 4.62 (t, *J* = 6.0 Hz, 2H, propyl-CH_2_), 4.19 (t, *J* = 6.0 Hz, 2H, propyl-CH_2_), 3.92 (s, 3H, OCH_3_), 2.42–2.35 (m, 2H, propyl-CH_2_). ^13^C NMR (100 MHz, DMSO-*d_6_*) *δ* 157.14 (quinazoline-C4), 156.38 (quinazoline-C7), 155.02 (Ar-C), 153.18 (imidazole-C2), 148.40 (quinazoline-C2), 147.66 (quinazoline-C6), 145.60 (quinazoline-C8a), 138.35 (Ar-C), 132.48 (Ar-C), 132.04 (Ar-C), 128.34 (Ar-C), 128.24 (imidazole-C5), 125.36 (imidazole-C4), 125.27 (Ar-C), 123.69 (Ar-C), 122.87 (Ar-C), 122.47 (Ar-C), 118.87 (Ar-C), 117.21 (Ar-C), 109.23 (quinazoline-C4a), 107.81 (quinazolie-C8), 103.35 (quinazoline-C5), 66.54 (propyl-CH_2_), 56.36 (OCH_3_), 47.43 (propyl-CH_2_), and 29.61 (propyl-CH_2_). HRMS (ESI) *m/z* calculated for C_27_H_22_C_l3_N_6_O_5_ [M + H]^+^: 615.0717, found: 615.0723.

*N-(3-Chloro-4-(3,4-dichlorophenoxy)phenyl)-7-methoxy-6-(4-(2-nitro-1H-imidazol-1-yl)butoxy)quinazolin-4-amine (***9b***).* Yellow solid (74 mg, 0.12 mmol). m.p.: 101–102 °C. HPLC purity: 96.17% (RT = 15.333 min). ^1^H NMR (400 MHz, DMSO-*d_6_*) *δ* 9.56 (s, 1H, NH), 8.52 (s, 1H, quinazoline-C2-H), 8.19 (d, *J* = 4.0 Hz, 1H, Ar-H), 7.86 (dd, *J* = 9.2, 2.4 Hz, 1H, Ar-H), 7.81 (s, 1H, quinazoline-C8-H), 7.76 (d, *J* = 4.0 Hz, 1H, imidazole-C5-H), 7.62 (d, *J* = 12.0 Hz, 1H, Ar-H), 7.32 (d, *J* = 8.0 Hz, 1H, imidazole-C4-H), 7.24 (d, *J* = 4.0 Hz, 1H, Ar-H), 7.21 (s, 1H, quinazoline-C5-H), 7.19 (d, *J* = 0.8 Hz, 1H, Ar-H), 6.93 (dd, *J* = 8.0, 4.0 Hz, 1H, Ar-H), 4.50 (t, *J* = 8.0 Hz, 2H, butyl-CH_2_), 4.18 (t, *J* = 6.0 Hz, 2H, butyl-CH_2_), 3.92 (s, 3H, OCH_3_), 2.04–1.97 (m, 2H, butyl-CH_2_), and 1.86–1.79 (m, 2H, butyl-CH_2_). ^13^C NMR (100 MHz, DMSO-*d_6_*) *δ* 157.14 (quinazoline-C4), 156.35 (quinazoline-C7), 155.00 (Ar-C), 153.07 (imidazole-C2), 148.66 (quinazoline-C2), 147.49 (quinazoline-C6), 145.59 (quinazoline-C8a), 138.36 (Ar-C), 132.48 (Ar-C), 132.04 (Ar-C), 128.35 (Ar-C), 128.31 (imidazole-C_5_), 125.36 (imidazole-C_4_), 125.26 (Ar-C), 123.71 (Ar-C), 122.85 (Ar-C), 122.50 (Ar-C), 118.86 (Ar-C), 117.21 (Ar-C), 109.30 (quinazoline-C4a), 107.77 (quinazoline-C8), 103.15 (quinazoline-C5), 68.96 (butyl-CH_2_), 56.36 (OCH_3_), 49.70 (butyl-CH_2_), 27.15 (butyl-CH_2_), and 25.88 (butyl-CH_2_). HRMS (ESI) *m/z* calculated for C_28_H_24_C_l3_N_6_O_5_ [M + H]^+^: 629.0874, found: 629.0881.

*N-(3-Chloro-4-(3,4-dichlorophenoxy)phenyl)-7-methoxy-6-((5-(2-nitro-1H-imidazol-1-yl)pentyl)oxy)quinazolin-4-amine (***9c***).* Yellow solid (98.3 mg, 0.15 mmol). m.p.: 94–95 °C. HPLC purity: 97.95% (RT = 15.902 min). ^1^H NMR (400 MHz, DMSO-*d_6_*) *δ* 9.55 (s, 1H, NH), 8.51 (s, 1H, quinazoline-C2-H), 8.19 (d, *J* = 4.0 Hz, 1H, Ar-H), 7.89 (dd, *J* = 8.8, 2.8 Hz, 1H, Ar-H), 7.80 (s, 1H, quinazoline-C8-H), 7.71 (d, *J* = 0.4 Hz, 1H, imidazole-C5-H), 7.62 (d, *J* = 12.0 Hz, 1H, Ar-H), 7.32 (d, *J* = 8.0 Hz, 1H, imidazole-C4-H), 7.24 (d, *J* = 4.0 Hz, 1H, Ar-H), 7.20 (s, 1H, quinazoline-C5-H), 7.18 (d, *J* = 0.8 Hz, 1H, Ar-H), 6.93 (dd, *J* = 8.0, 4.0 Hz, 1H, Ar-H), 4.43 (t, *J* = 6.0 Hz, 2H, pentyl-CH_2_), 4.13 (t, *J* = 8.0 Hz, 2H, pentyl-CH_2_), 3.92 (s, 3H, OCH_3_), 1.92–1.83 (m, 4H, pentyl-2CH_2_), and 1.51–1.44 (m, 2H, pentyl-CH_2_). ^13^C NMR (100 MHz, DMSO-*d_6_*) *δ* 157.15 (quinazoline-C4), 156.33 (quinazoline-C7), 154.98 (Ar-C), 153.02 (imidazole-C2), 148.79 (quinazoline-C2), 147.45 (quinazoline-C6), 145.57 (quinazoline-C8a), 138.38 (Ar-C), 132.48 (Ar-C), 132.04 (Ar-C), 128.35 (Ar-C), 128.30 (imidazole-C5), 125.35 (imidazole-C4), 125.26 (Ar-C), 123.72 (Ar-C), 122.85 (Ar-C), 122.51 (Ar-C), 118.85 (Ar-C), 117.20 (Ar-C), 109.31 (quinazoline-C4a), 107.74 (quinazoline-C8), 102.89 (quinazoline-C5), 69.04 (pentyl-CH_2_), 56.33 (OCH_3_), 49.75 (pentyl-CH_2_), 30.00 (pentyl-CH_2_), 28.56 (pentyl-CH_2_), and 23.00 (pentyl-CH_2_). HRMS (ESI) *m/z* calculated for C_29_H_26_C_l3_N_6_O_5_ [M + H]^+^: 643.1030, found: 643.1031.

*N-(3-Chloro-4-(3,4-dichlorophenoxy)phenyl)-7-methoxy-6-((6-(2-nitro-1H-imidazol-1-yl)hexyl)oxy)quinazolin-4-amine (***9d***).* Yellow solid (81 mg, 0.12 mmol). m.p.: 90–91 °C. HPLC purity: 95.31% (RT = 16.266 min). ^1^H NMR (400 MHz, DMSO-*d_6_*) *δ* 9.55 (s, 1H, NH), 8.51 (s, 1H, quinazoline-C2-H), 8.19 (d, *J* = 4.0 Hz, 1H, Ar-H), 7.87 (dd, *J* = 8.0, 4.0 Hz, 1H, Ar-H), 7.79 (s, 1H, quinazoline-C8-H), 7.69 (s, 1H, Ar-H), 7.61 (d, *J* = 8.0 Hz, 1H, imidazole-C5-H), 7.32 (d, *J* = 12.0 Hz, 1H, imidazole-C4-H), 7.23 (d, *J* = 4.0 Hz, 1H, Ar-H), 7.19 (s, 1H, quinazoline-C5-H), 7.16 (s, 1H, Ar-H), 6.92 (dd, *J* = 8.0, 4.0 Hz, 1H, Ar-H), 4.38 (t, *J* = 8.0 Hz, 2H, hexyl-CH_2_), 4.12 (t, *J* = 8.0 Hz, 2H, hexyl-CH_2_), 3.92 (s, 3H, OCH_3_), 1.86–1.77 (m, 4H, hexyl-2CH_2_), 1.54–1.46 (m, 2H, hexyl-CH_2_), and 1.41–1.34 (m, 2H, hexyl-CH_2_). ^13^C NMR (100 MHz, DMSO-*d_6_*) *δ* 157.15 (quinazoline-C4), 156.33 (quinazoline-C7), 154.99 (Ar-C), 153.00 (imidazole-C2), 148.85 (quinazoline-C2), 147.44 (quinazoline-C6), 145.57 (quinazoline-C8a), 138.39 (Ar-C), 132.48 (Ar-C), 132.04 (Ar-C), 128.28 (Ar-C), 128.25 (imidazole-C_5_), 125.35 (imidazole-C4), 125.25 (Ar-C), 123.72 (Ar-C), 122.85 (Ar-C), 122.52 (Ar-C), 118.85 (Ar-C), 117.21 (Ar-C), 109.32 (quinazoline-C4a), 107.74 (quinazoline-C8), 102.86 (quinazoline-C5), 69.12 (hexyl-CH_2_), 56.33 (OCH_3_), 49.80 (hexyl-CH_2_), 30.17 (hexyl-CH_2_), 28.92 (hexyl-CH_2_), 26.05 (hexyl-CH_2_), and 25.56 (hexyl-CH_2_). HRMS (ESI) *m/z* calculated for C_30_H_28_C_l3_N_6_O_5_ [M + H]^+^: 657.1187, found: 657.1184.

*N-(3-Chloro-4-(3-(trifluoromethyl)phenoxy)phenyl)-7-methoxy-6-(3-(2-nitro-1H-imidazol-1-yl)propoxy)quinazolin-4-amine (***9e***).* Yellow solid (97.2 mg, 0.16 mmol). m.p.: 232–233 °C. HPLC purity: 98.08% (RT = 14.336 min). ^1^H NMR (400 MHz, DMSO-*d_6_*) *δ* 9.58 (s, 1H, NH), 8.54 (s, 1H, quinazoline-C2-H), 8.22 (d, *J* = 4.0 Hz, 1H, Ar-H), 7.88 (dd, *J* = 9.0, 2.6 Hz, 1H, Ar-H), 7.84 (s, 1H, quinazoline-C8-H), 7.69 (s, 1H, Ar-H), 7.63 (t, *J* = 8.0 Hz, 1H, Ar-H), 7.49 (d, *J* = 8.0 Hz, 1H, imidazole-C5-H), 7.36 (d, *J* = 6.0 Hz, 1H, imidazole-C4-H), 7.25–7.23 (m, 3H, quinazoline-C5-H and Ar-H), 7.19 (d, *J* = 0.8 Hz, 1H, Ar-H), 4.64 (t, *J* = 8.0 Hz, 2H, propyl-CH_2_), 4.22 (t, *J* = 6.0 Hz, 2H, propyl-CH_2_), 3.95 (s, 3H, OCH_3_), and 2.43–2.38 (m, 2H, propyl-CH_2_). ^13^C NMR (100 MHz, DMSO-*d_6_*) *δ* 158.14 (quinazoline-C4), 156.39 (quinazoline-C7), 155.02 (Ar-C), 153.18 (imidazole-C2), 148.39 (quinazoline-C2), 147.65 (quinazoline-C6), 145.58 (quinazoline-C8a), 145.20 (Ar-C), 138.31 (Ar-C), 131.89 (Ar-C), 131.30 (Ar-C), 130.98 (imidazole-C5), 128.34 (imidazole-C4), 128.23 (Ar-C), 125.45 (CF_3_), 123.75 (Ar-C), 123.02 (Ar-C), 122.55 (Ar-C), 120.65 (Ar-C), 119.87 (Ar-C), 113.22 (quinazoline-C4a), 109.23 (Ar-C), 107.80 (quinazoline-C8), 103.34 (quinazoline-C5), 66.54 (propyl-CH_2_), 56.35 (OCH_3_), 47.43 (propyl-CH_2_), and 29.61 (propyl-CH_2_). HRMS (ESI) *m/z* calculated for C_28_H_23_ClF_3_N_6_O_5_ [M + H]^+^: 615.1371, found: 615.1371.

*N-(3-Chloro-4-(3-(trifluoromethyl)phenoxy)phenyl)-7-methoxy-6-(4-(2-nitro-1H-imidazol-1-yl)butoxy)quinazolin-4-amine (***9f***).* Yellow solid (53.1 mg, 0.08 mmol). m.p.: 103–104 °C. HPLC purity: 95.07% (RT = 14.794 min). ^1^H NMR (400 MHz, DMSO-*d_6_*) *δ* 9.57 (s, 1H, NH), 8.52 (s, 1H, quinazoline-C2-H), 8.20 (d, *J* = 4.0 Hz, 1H, Ar-H), 7.87 (dd, *J* = 9.2, 2.8 Hz, 1H, Ar-H), 7.82 (s, 1H, quinazoline-C8-H), 7.75 (s, 1H, Ar-H), 7.61 (t, *J* = 8.0 Hz, 1H, Ar-H), 7.47 (d, *J* = 4.0 Hz, 1H, imidazole-C5-H), 7.34 (d, *J* = 12.0 Hz, 1H, imidazole-C4-H), 7.23–7.19 (m, 4H, quinazoline-C5-H and Ar-H), 4.50 (t, *J* = 8.0 Hz, 2H, butyl-CH_2_), 4.18 (t, *J* = 8.0 Hz, 2H, butyl-CH_2_), 3.92 (s, 3H, OCH_3_), 2.03–1.97 (m, 2H, butyl-CH_2_), and 1.85–1.79 (m, 2H, butyl-CH_2_). ^13^C NMR (100 MHz, DMSO-*d_6_*) *δ* 157.60 (quinazoline-C4), 155.83 (quinazoline-C7), 154.47 (Ar-C), 152.55 (imidazole-C2), 148.12 (quinazoline-C2), 146.98 (quinazoline-C6), 145.04 (quinazoline-C8a), 144.52 (Ar-C), 137.78 (Ar-C), 131.35 (Ar-C), 130.75 (Ar-C), 130.43 (imidazole-C5), 127.80 (imidazole-C4), 127.76 (Ar-C), 124.89 (CF_3_), 123.24 (Ar-C), 122.47 (Ar-C), 122.06 (Ar-C), 120.11 (Ar-C), 119.33 (Ar-C), 112.62 (quinazoline-C4a), 108.75 (Ar-C), 107.26 (quinazoline-C8), 102.62 (quinazoline-C5), 68.41 (butyl-CH_2_), 55.82 (OCH_3_), 49.15 (butyl-CH_2_), 26.60 (butyl-CH_2_), and 25.33 (butyl-CH_2_). HRMS (ESI) *m/z* calculated for C_29_H_25_ClF_3_N_6_O_5_ [M + H]^+^: 629.1527, found: 629.1528.

*N-(3-Chloro-4-(3-(trifluoromethyl)phenoxy)phenyl)-7-methoxy-6-((5-(2-nitro-1H-imidazol-1-yl)pentyl)oxy)quinazolin-4-amine (***9g***).* Yellow solid (103.4 mg, 0.16 mmol). m.p.: 90–91 °C. HPLC purity: 95.59% (RT = 15.254 min). ^1^H NMR (400 MHz, DMSO-*d_6_*) *δ* 9.56 (s, 1H, NH), 8.51 (s, 1H, quinazoline-C2-H), 8.20 (d, *J* = 4.0 Hz, 1H, Ar-H), 7.87 (dd, *J* = 8.8, 2.4 Hz, 1H, Ar-H), 7.80 (s, 1H, quinazoline-C8-H), 7.71 (d, *J* = 0.4 Hz, 1H, Ar-H), 7.61 (t, *J* = 8.0 Hz, 1H, Ar-H), 7.47 (d, *J* = 8.0 Hz, 1H, imidazole-C5-H), 7.34 (d, *J* = 12.0 Hz, 1H, imidazole-C4-H), 7.22–7.18 (m, 4H, quinazoline-C5-H and Ar-H), 4.43 (t, *J* = 8.0 Hz, 2H, pentyl-CH_2_), 4.13 (t, *J* = 8.0 Hz, 2H, pentyl-CH_2_), 3.92 (s, 3H, OCH_3_), 1.91–1.83 (m, 4H, pentyl-2CH_2_), and 1.52–1.44 (m, 2H, pentyl-CH_2_). ^13^C NMR (100 MHz, DMSO-*d_6_*) *δ* 158.15 (quinazoline-C4), 156.34 (quinazoline-C7), 154.98 (Ar-C), 153.03 (imidazole-C2), 148.79 (quinazoline-C2), 147.45 (quinazoline-C6), 145.56 (quinazoline-C8a), 145.00 (Ar-C), 138.35 (Ar-C), 131.89 (Ar-C), 131.30 (Ar-C), 130.98 (imidazole-C5), 128.34 (imidazole-C4), 128.29 (Ar-C), 125.43 (CF_3_), 123.78 (Ar-C), 123.00 (Ar-C), 122.60 (Ar-C), 120.65 (Ar-C), 119.86 (Ar-C), 113.20 (quinazoline-C4a), 109.31 (Ar-C), 107.75 (quinazoline-C8), 102.90 (quinazoline-C5), 69.04 (pentyl-CH_2_), 56.32 (OCH_3_), 49.75 (pentyl-CH_2_), 30.00 (pentyl-CH_2_), 28.56 (pentyl-CH_2_), and 23.00 (pentyl-CH_2_). HRMS (ESI) *m/z* calculated for C_30_H_27_ClF_3_N_6_O_5_ [M + H]^+^: 643.1684, found: 643.1693.

*N-(3-Chloro-4-(3-(trifluoromethyl)phenoxy)phenyl)-7-methoxy-6-((6-(2-nitro-1H-imidazol-1-yl)hexyl)oxy)quinazolin-4-amine (***9h***).* Yellow solid (134.3 mg, 0.20 mmol). m.p.: 84–85 °C. HPLC purity: 96.10% (RT = 15.657 min). ^1^H NMR (400 MHz, DMSO-*d_6_*) *δ* 9.56 (s, 1H, NH), 8.51 (s, 1H, quinazoline-C2-H), 8.20 (d, *J* = 2.4 Hz, 1H, Ar-H), 7.88 (dd, *J* = 8.0, 4.0 Hz, 1H, Ar-H), 7.81 (s, 1H, quinazoline-C8-H), 7.69 (s, 1H, Ar-H), 7.61 (t, *J* = 8.0 Hz, 1H, Ar-H), 7.47 (d, *J* = 8.0 Hz, 1H, imidazole-C5-H), 7.33 (d, *J* = 8.0 Hz, 1H, imidazole-C4-H), 7.22–7.20 (m, 3H, quinazoline-C5-H and Ar-H), 7.16 (s, 1H, Ar-H), 4.38 (t, *J* = 6.0 Hz, 2H, hexyl-CH_2_), 4.13 (t, *J* = 6.0 Hz, 2H, hexyl-CH_2_), 3.93 (s, 3H, OCH_3_), 1.87–1.78 (m, 4H, hexyl-2CH_2_), 1.54–1.47 (m, 2H, hexyl-CH_2_), and 1.42–1.34 (m, 2H, hexyl-CH_2_). ^13^C NMR (100 MHz, DMSO-*d_6_*) *δ* 158.15 (quinazoline-C4), 156.33 (quinazoline-C7), 154.97 (Ar-C), 153.00 (imidazole-C2), 148.84 (quinazoline-C2), 147.44 (quinazoline-C6), 145.54 (quinazoline-C8a), 144.98 (Ar-C), 138.36 (Ar-C), 131.87 (Ar-C), 131.30 (Ar-C), 130.98 (imidazole-C5), 128.28 (imidazole-C4), 128.23 (Ar-C), 125.43 (CF_3_), 123.77 (Ar-C), 122.97 (Ar-C), 122.59 (Ar-C), 120.63 (Ar-C), 119.88 (Ar-C), 113.16 (quinazoline-C4a), 109.32 (Ar-C), 107.73 (quinazoline-C8), 102.84 (quinazoline-C5), 69.11 (hexyl-CH_2_), 56.30 (OCH_3_), 49.80 (hexyl-CH_2_), 30.17 (hexyl-CH_2_), 28.92 (hexyl-CH_2_), 26.06 (hexyl-CH_2_), and 25.56 (hexyl-CH_2_). HRMS (ESI) *m/z* calculated for C_31_H_29_ClF_3_N_6_O_5_ [M + H]^+^: 657.1840, found: 657.1841.

### *In vitro* kinase assays

Enzyme inhibitory assays were carried out as described in our previous reports in the literature^33–35^. Briefly, the Kinase HotSpot^SM^ service from Reaction Biology Co. (Malvern, PA) was used to screen the tested compounds. The HotSpot assay platform includes specific kinase/substrate pairs and required cofactors. The base reaction buffer consisted of 20 mM Hepes (pH 7.5), 10 mM MgCl_2_, 1 mM EGTA, 0.02% Brij35, 0.02 mg/mL BSA, 0.1 mM Na_3_VO_4_, 2 mM DTT, and 1% DMSO. The tested compounds were dissolved in 100% DMSO to a specific concentration, and serial dilution was conducted. The reaction mixture containing the tested compound and 33P-ATP was incubated at room temperature for 2 h, and radioactivity was detected using the filter-binding method.

### Cell culture

Prostate cancer cell lines (PC3 and 22RV1) of American Type Culture Collection (ATCC) were obtained from Korean Cell Center (KCL). PC3 and 22RV1 cell lines were cultured in Roswell Park Memorial Institute Medium (RPMI) 1640 and Dulbecco’s modified Eagle’s medium (DMEM) (GenDepot, Barker, TX) supplemented with 1% penicillin–streptomycin and 10% foetal bovine serum (FBS). The cells were maintained at 37 °C in a 5% CO_2_ with a 95% humid atmosphere.

### MTT assay

To further analyse the intracellular cytotoxicity effect of the as-synthesised compounds, MTT assay was performed. Moreover, the experiments were divided into subcategories. In each group, cancer cells were first seeded in 96-well plates (4 × 10^4^ cells per well) and then incubated for 24 h at 37 °C in a 5% CO_2_-containing atmosphere. Subsequently, the medium from the culture plate was removed, and 100 µL of RPMI media containing different concentrations of compounds (0.01, 0.005, 0.0025, 0.00125, and 0.000625 µM) were then added into the cell lines in each well and incubated for 24 h in a 5% CO_2_ humidified incubator at 37 °C. Afterwards, MTT solution (150 μL, 1 mg/mL) was added to each well, replacing the media-containing compounds. After 4 h of incubation, the MTT reagent was removed, and 200 μL of DMSO was then added to dissolve the formazan crystals, and colour intensity was measured at a wavelength of 540 nm using a BioTek Synergy H1 instrument (BioTek, Winooski, VT). IC_50_ was calculated according to the equation of Boltzmann sigmoidal concentration–response curve using Graph Pad Prism 5 (La Jolla, CA).

### Analysis of the cell cycle distribution

To determine the effect of the tested compound on the cell cycle distribution of PC3 and 22RV1 cell lines. Cell cycles can be analysed with checking the cell cycle change compared to the control group. Control cells without any treatment were used as a reference point for determining the cell cycle arrest phase for the test sample. Cell cycle analysis was performed using the Cell Cycle Analysis Kit (ADAMII LS, NanoEntek, Seoul, South Korea). Briefly, cancer cells were first seeded in six-well plates (0.8 × 10^6^ cells per well) and then incubated for 24 h at 37 °C in a 5% CO_2_-containing atmosphere. Subsequently, the medium from the culture plate was removed, and 3 mL of RPMI and DMEM media containing concentrations of compound **9f** at its IC_50_ value were then added into the cells in each well and incubated for 24 h in a 5% CO_2_ humidified incubator at 37 °C. Afterward, the media was removed following the washing once with PBS and the 25 µL cell sample mixtures were stained with 25 µL PI stain. 25 µL of sample mixture was loaded into ADAMII LS assay slide, then the slide was incubated at room temperature for 1 min in the dark and run on the ADAMII LS fluorescence cell analyser. Cell cycle distribution was calculated using ADAMII LS software (ADAMII LS, NanoEntek, Seoul, South Korea).

### Apoptosis analysis

The apoptotic effect of a potent compound on PC3 and 22RV1 cell lines was assessed using apoptosis analysis with Annexin V-PE, DAPI solution (ADAMII LS, NanoEntek, Seoul, South Korea). Early and late apoptotic effects were analysed compared to the control. Cells were seeded in a six-well plate with cells density 0.8 × 10^5^ cells/well, in a volume of 3 mL RPMI and DMEM medium and incubated for 24 h at 37 °C in a 5% CO_2_-containing atmosphere. Subsequently, the medium from the culture plate was replaced by 3 mL of RPMI and DMEM media containing compound **9f** at its IC_50_ value and incubated for 24 h at 37 °C and in the presence of 5% CO_2_. The apoptosis assay was performed using the ADAMII LS apoptosis analysis kit. Briefly, the apoptosis-induced cell sample was prepared using a cell scraper (alfa aesar) after washing with phosphate-buffered saline (PBS). The cell was resuspended in 100 µL 1X Annexin V binding buffer, and 5 µL Annexin V-PE reagent was added afterward and incubated for 15 min at room temperature. The sample was subsequently centrifuged, and the pallet was resuspended again with 500 µL 1X Annexin V binding buffer and added 1.25 µL DAPI reagent. The slide loaded with the prepared sample was incubated at room temperature for 1 min in the dark and run on the ADAMII LS fluorescence cell analyser. The comparative data including dot plot graphs and images were calculated and analysed using ADAMII LS software (NanoEntek, Seoul, South Korea).

### Statistical analysis

The statistical analysis of the data was done by standard deviations and all values represent the mean ± SD of three independent experiments.

### Molecular docking

Two molecular docking processes were performed for the newly designed and synthesised derivatives (**9a**–**h**) towards the two target receptors (EGFR and HER2) using the MOE 2019.0102^31^^,^^32^. Besides, the co-crystallised ligands were incorporated as reference controls. The designed candidates (**9a**–**h**) were sketched using the ChemDraw program, transferred into the MOE window, and prepared to be inserted into the same database besides the co-crystallised inhibitor in each case, as previously discussed^36^^,^^37^. The X-ray structures of the target receptors (EGFR and HER2) with PDB IDs: 1M17^30^ and 3RCD^25^, respectively, were downloaded, protonated, and prepared for the docking process as described earlier^38^^,^^39^. Then, two docking processes (general subtypes) were performed within the EGFR and HER2 receptors in the presence of the corresponding prepared database in each case. The items of the program specifications were adjusted to the general docking subtype process, as mentioned before^40^^,^^41^. Finally, we redocked the native co-crystallised inhibitors of both EGFR and HER2 receptors within their binding pockets to validate the applied forcefield. Herein, the validity was confirmed by observing the same binding modes of the superimposed docked co-crystallised inhibitor in each case over its native one^42^. Moreover, promising root mean square deviation (RMSD) values were obtained for EGFR and HER2 receptors (1.32 and 1.76 Å, respectively).

### Molecular dynamics simulations and MM-GBSA study

The MD simulations and MM-GBSA study were performed following same procedures found in our earlier report^24^.

## Conclusions

EGFR and HER2 have been co-expressed and recognised in numerous solid tumours. Successfully, new TAK-285 derivatives (**9a**–**h**) were synthesised and assessed as EGFR/HER2 dual inhibitors. Applying “HotSpot^SM^” assay at 10 µM concentration in the presence of 10 µM of ATP, all compounds **9a**–**h** exhibited encouraging percentage inhibition ranges against both kinases. The most active candidate (**9f**) showed nanomolar IC_50_ values of 2.3 nM over EGFR and 234 nM over HER2. A kinase selectivity panel of derivative **9f** indicated a potential selective profile. Potent nanomolar IC_50_ values of compounds were obtained against PC3 and 22RV1 cell lines. The cell cycle of 22RV1 and PC3 cells was inhibited by compound **9f** by 62.74 and 49.43% in the G2/M phase, respectively. Also, cells treated with compound **9f** underwent early apoptosis. Molecular simulation studies revealed insights about the binding mode of the designed compounds. Overall, we report compound **9f** as a new potent dual EGFR/HER2 inhibitor with significant cytotoxic activity against 22RV1 and PC3 prostate carcinoma cell lines and could be a potent anticancer drug for future medication.

## Supplementary Material

Supplemental Material
