# Supplementary material for: Development of new TAK-285 derivatives as potent EGFR/HER2 inhibitors possessing antiproliferative effects against 22RV1 and PC3 prostate carcinoma cell lines
Source: J Enzyme Inhib Med Chem. 2023 Apr 25;38(1):2202358. doi: 10.1080/14756366.2023.2202358 (PMC10132233; doi:10.1080/14756366.2023.2202358)
Supplement: Supplemental Material [file IENZ_A_2202358_SM9724.pdf]

Supplementary material

Development of new TAK-285 derivatives as potent EGFR/HER2 inhibitors possessing antiproliferative effects against 22RV1 and PC3 prostate carcinoma cell lines

Seohyun Son [1], Ahmed Elkamhawy [1,2], Anam Rana Gul [3], Ahmed A. Al-Karmalawy [4], Radwan Alnajjar [5,6,7], Ahmed Abdeen [8], Samah F. Ibrahim [9], Saud O. Alshammari [10], Qamar A. Alshammari [11], Won Jun Choi [1], Tae Jung Park [3,*], Kyeong Lee [1,*]

[1] *BK21 FOUR Team and Integrated Research Institute for Drug Development, College of Pharmacy, Dongguk University-Seoul, Goyang, 10326, Republic of Korea*
[2] *Department of Pharmaceutical Organic Chemistry, Faculty of Pharmacy, Mansoura University, Mansoura 35516, Egypt*
[3] *Department of Chemistry, Research Institute of Chem-Bio Diagnostic Technology, Chung-Ang University, 84 Heukseok-ro, Dongjak-gu, Seoul 06974, Republic of Korea*
[4] *Pharmaceutical Chemistry Department, Faculty of Pharmacy, Ahram Canadian University, 6th of October City, Giza 12566, Egypt*
[5] *Department of Chemistry, Faculty of Science, University of Benghazi, Benghazi, Libya*
[6] *PharmD, Faculty of Pharmacy, Libyan International Medical University, Benghazi, Libya*
[7] *Department of Chemistry, University of Cape Town, Rondebosch 7701, South Africa*
[8] *Department of Forensic Medicine and Toxicology, Faculty of Veterinary Medicine, Benha University, Toukh 13736, Egypt*
[9] *Department of Clinical Sciences, College of Medicine, Princess Nourah bint Abdulrahman University, P.O. Box 84428, Riyadh 11671, Saudi Arabia*
[10] *Department of Plant Chemistry and Natural Products, Faculty of Pharmacy, Northern Border University, Arar 91431, Saudi Arabia*
[11] *Department of Pharmacology and Toxicology, Faculty of Pharmacy, Northern Border University, Arar 91431, Saudi Arabia*

[*] Co-corresponding authors

Tae Jung Park: tjpark@cau.ac.kr

Kyeong Lee: kaylee@dongguk.edu

Contents

1. **The general protocols utilized for the chemical synthesis, structure elucidation, and purity of the synthesized compounds**

2. **Molecular dynamics simulations**

3. **MD trajectory analysis and prime MM-GBSA calculations**

4. **^{1}H NMR spectrum of 4, 5, 7a-7b**

5. **^{1}H NMR spectrum of 8a-8b**

6. **^{1}H NMR and ^{13}C NMR spectrum of 9a-9h**

7. **HRMS chart of 9a-9h**

8. **HPLC purity chart of 9a-9h**

The general protocols utilized for the chemical synthesis, structure elucidation, and purity of the synthesized compounds

Commercially available reagents and solvents were used without further purification. All chemical reactions were monitored using TLC on 0.25 mm silica plates (E. Merck; silica gel 60 F254). The ^{1}H NMR spectra were acquired on Bruker 400 MHz spectrometer in dimethyl sulfoxide-d_6 (DMSO-d_6) as a solvent. Chemical shifts were confirmed in ppm units relative to the internal standard (tetramethylsilane), while coupling constants (J) were supplied in Hz units. The ^{13}C NMR spectra were performed on Varian 100 MHz spectrometer using DMSO-d_6 solvent. High-resolution mass spectra (HRMS) were acquired on Waters G2 QTOF mass spectrometer with an ESI source. The purity of the synthesized compounds was determined using reverse phase high-performance liquid chromatography (HPLC) using Waters 2695 system with PAD 996 with Welch Xtimate C18 column (150 mm x 4.6 mm x 5 μm; Condition: mobile phase A: 0.05% TFA in water, mobile phase B: CH_3CN; Gradient: 90% of Mobile phase B in 30 min.) at 281 nm. The melting points (M.p.) were determined by Thermo Scientific 9200 equipment (Temperature rise 10 °C per minute) and were not corrected.

SI 1. Molecular dynamics simulations

The MD simulations were carried out using Desmond simulation package of Schrödinger LLC [1]. The NPT ensemble with the temperature 300 K and a pressure 1 bar was applied in all runs. The simulation length was 200 ns with a relaxation time 1 ps for the ligands. The OPLS3 force field parameters were used in all simulations [2]. The cutoff radius in Coulomb interactions was 9.0 Å. The orthorhombic periodic box boundaries were set 10 Å away from the protein atoms. The water molecules were explicitly described using the transferable intermolecular potential with three points (TIP3P) model [3, 4]. Salt concentration set to 0.15 M NaCl and was built using the System Builder utility of Desmond [5]. The Martyna−Tuckerman−Klein chain coupling scheme with a coupling constant of 2.0 ps was used for the pressure control and the Nosé−Hoover chain coupling scheme for the temperature control [6, 7]. Nonbonded forces were calculated using a RESPA integrator where the short-range forces were updated every step and the long-range forces were updated every three steps. The trajectories were saved at 20 ns intervals for analysis. The behavior and interactions between the ligands and protein were analyzed using the Simulation Interaction Diagram tool implemented in Desmond MD

package. The stability of MD simulations was monitored by looking on the RMSD of the ligand and protein atom positions in time.

SI 2. MD trajectory analysis and prime MM-GBSA calculations

Simulation interactions diagram panel of Maestro software was used to monitoring interactions contribution in the ligand-protein stability. The molecular mechanics generalized born/solvent accessibility (MM – GBSA) was performed to calculate the ligand binding free energies and ligand strain energies for docked compounds over the last 50 ns with thermal_mmgbsa.py python script provided by Schrodinger which takes a Desmond trajectory file, splits it into individual snapshots, runs the MM-GBSA calculations on each frame, and outputs the average computed binding energy.

Figure SI 1: The Glu734---Lys851 distances in the two complexes during the simulations time

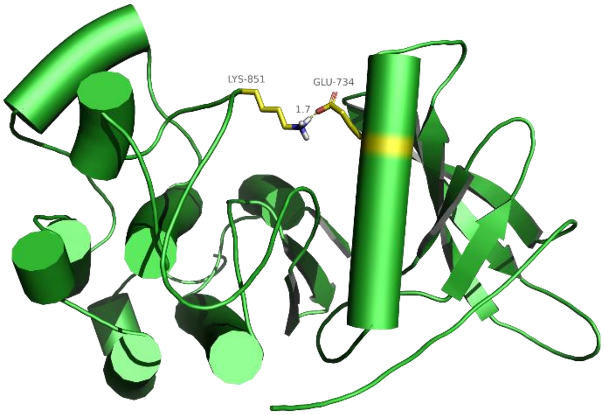

9f-1M17, 0ns
Glu734---Lys851 (1.7 Å)

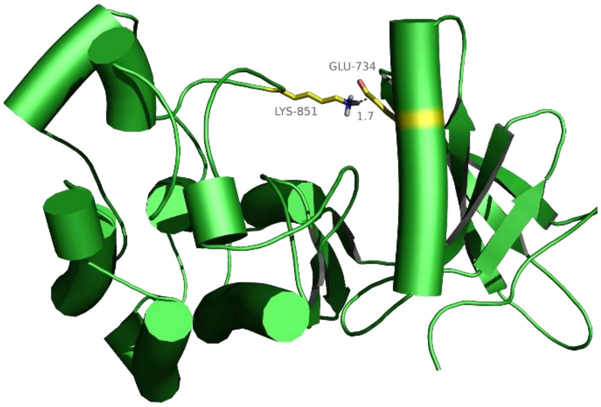

9f-1M17, 200ns
Glu734---Lys851 (1.7 Å)

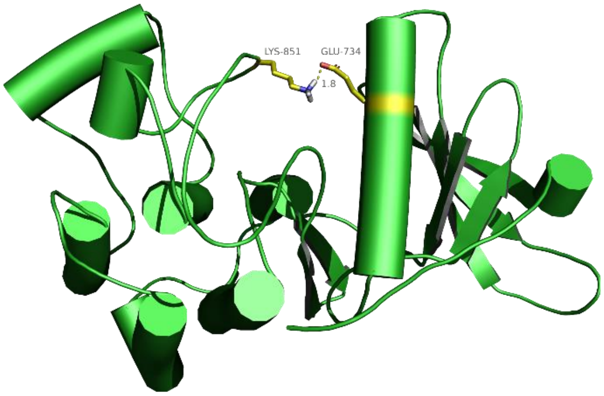

Co-1M17, 0ns
Glu734---Lys851 (1.8 Å)

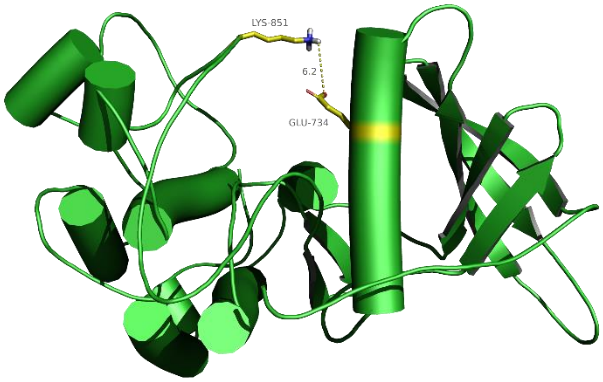

Co-1M17, 200ns
Glu734---Lys851 (6.2 Å)

Figure SI 2: Snapshots ever 20th ns of **9g**-3RCD complex showing the fluctuated unfolded loop (red)

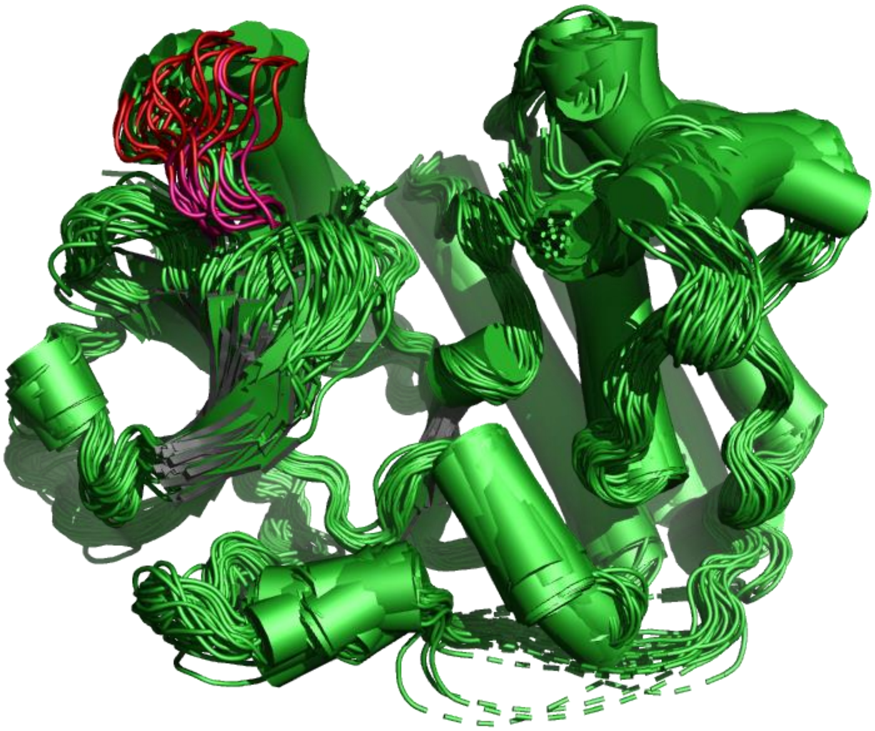

Figure SI 3: The histogram of Co-1M17 contact throughout the trajectory

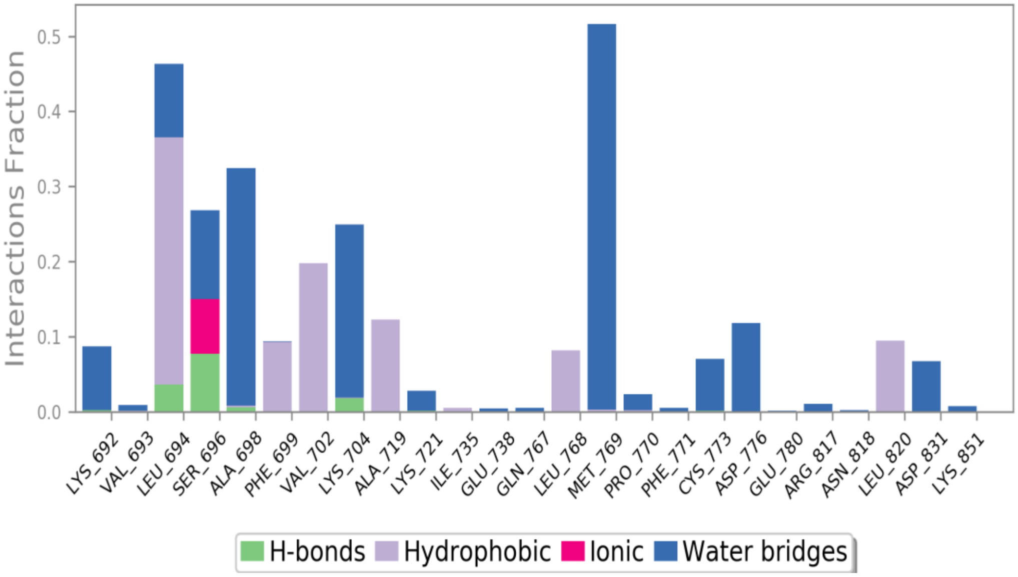

Figure SI 4: 9f-1M17 interactions that were observed more than 30% of the simulation time

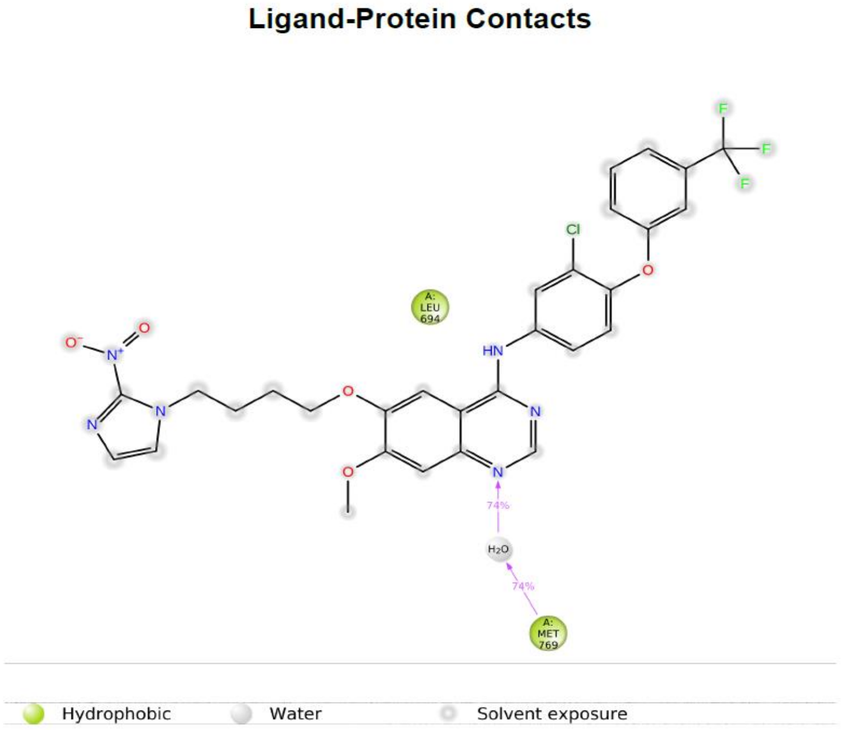

Figure SI 5: 9g-3RCD interactions that were observed more than 30% of the simulation time

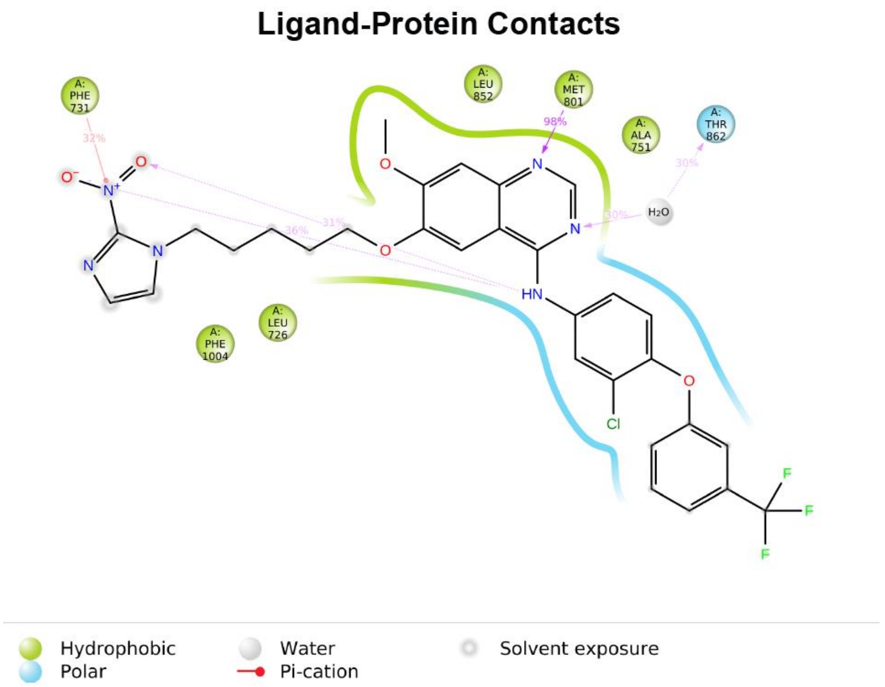

Figure SI 6. ^{1}H NMR spectrum of compound **4**

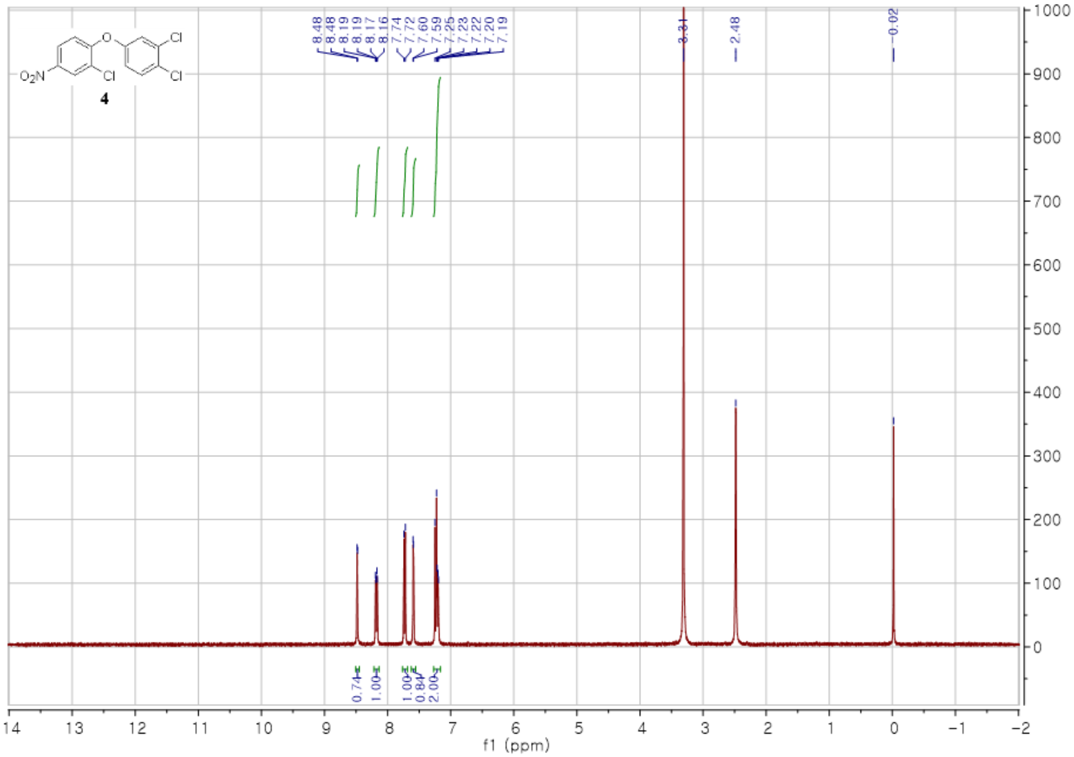

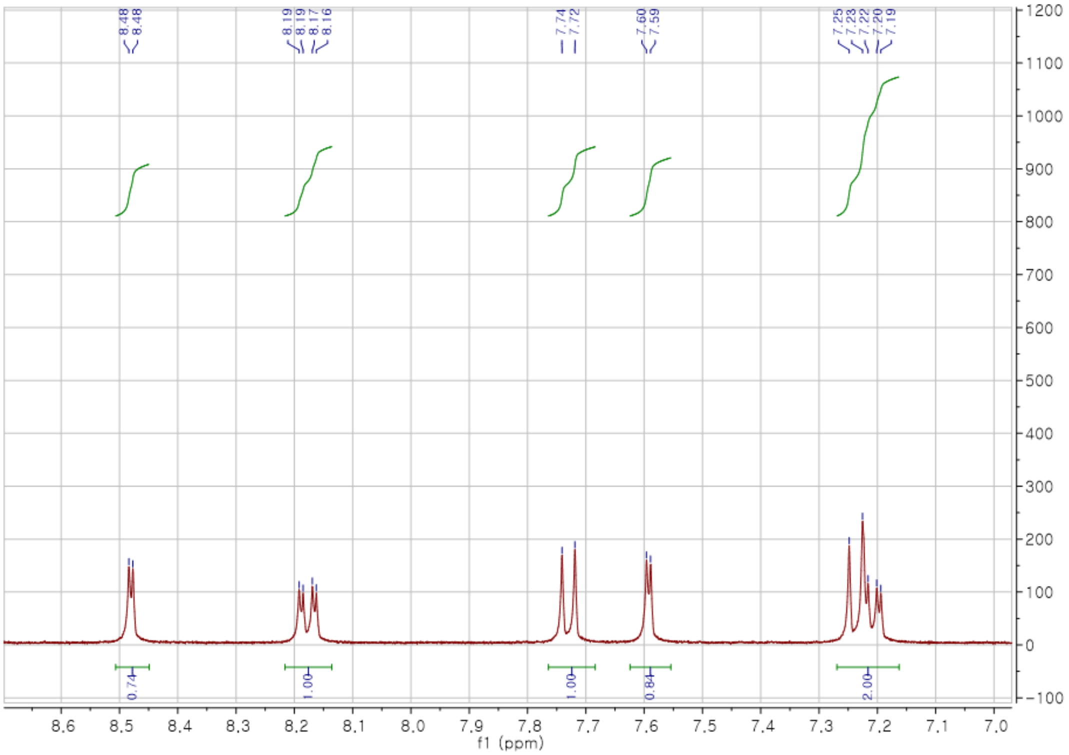

Figure SI 7. ^{1}H NMR spectrum of compound **5**

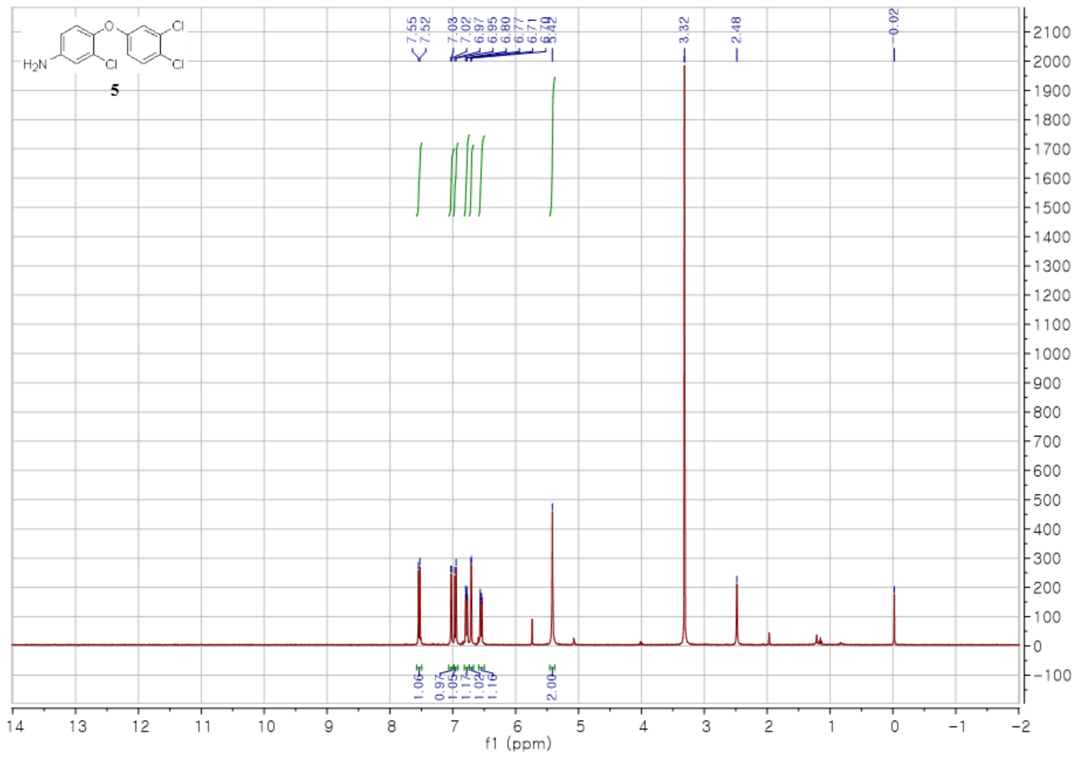

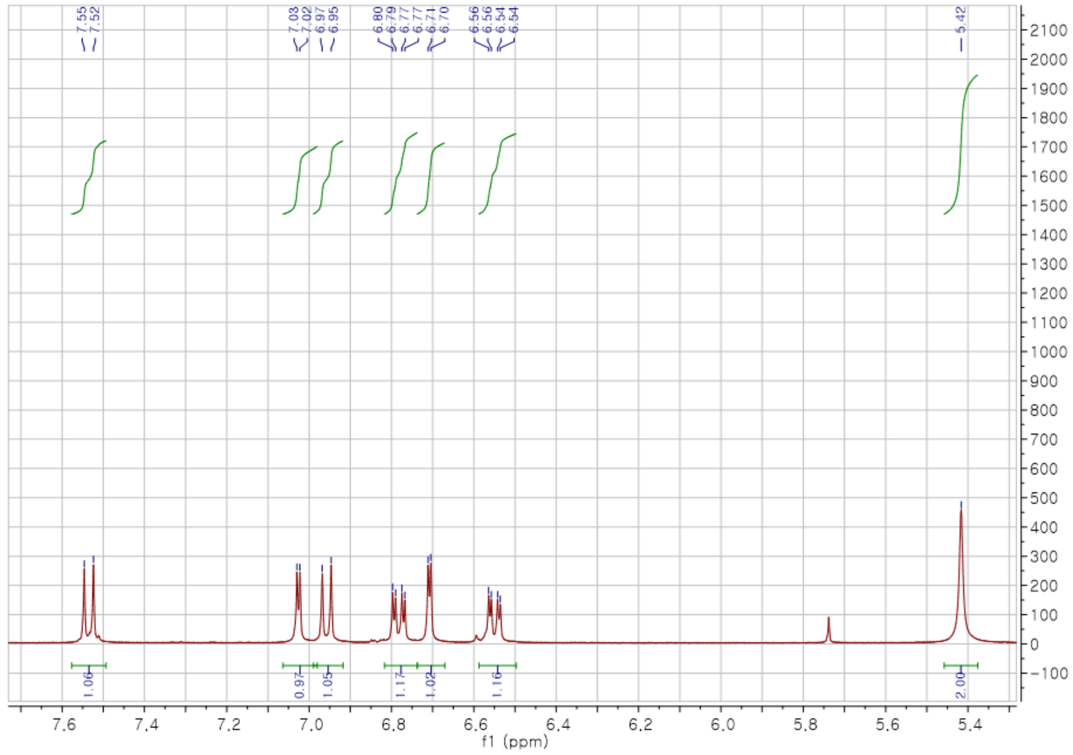

Figure SI 8. ^{1}H NMR spectrum of compound 7**a**

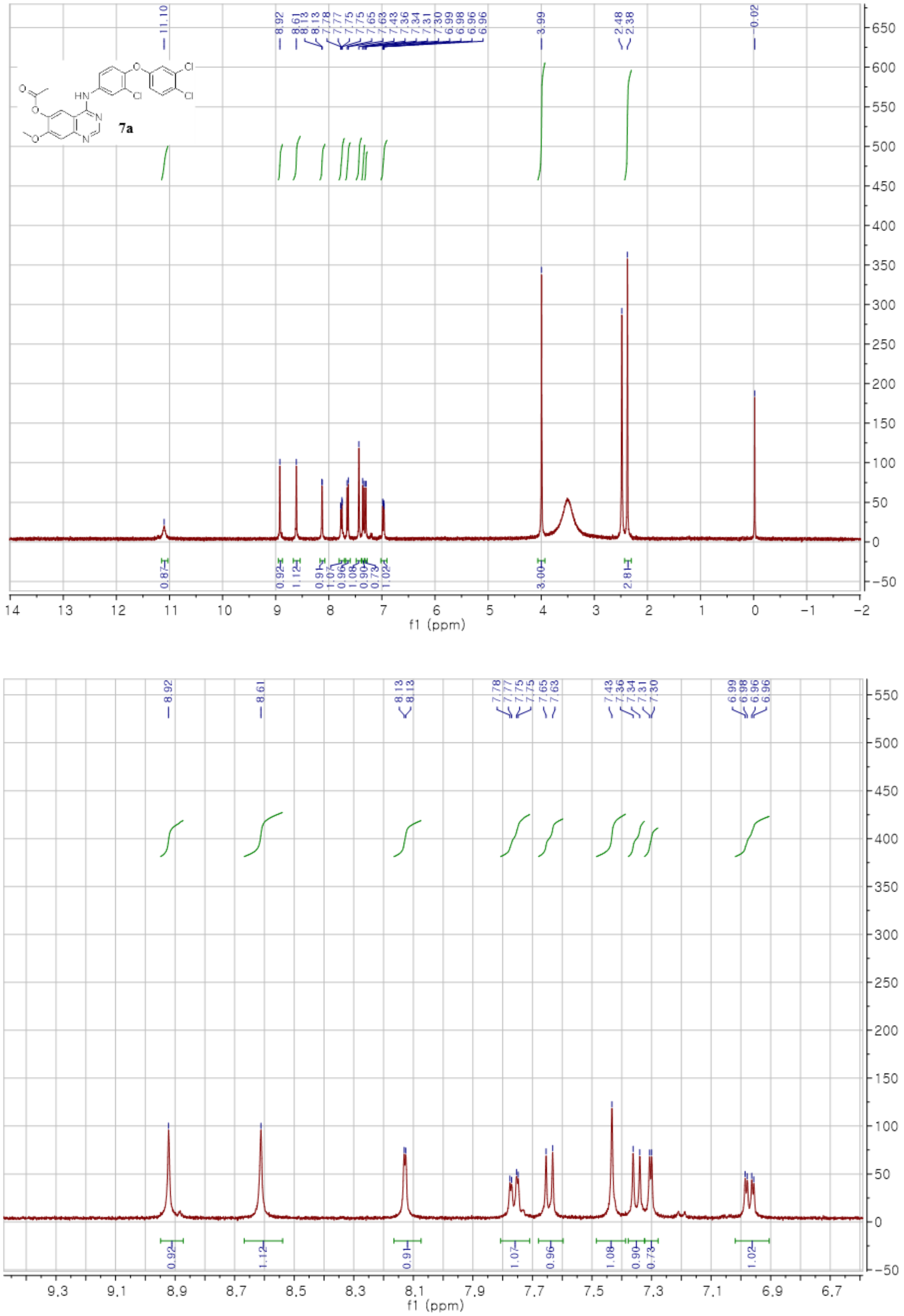

Figure SI 9. ^{1}H NMR spectrum of compound **7b**

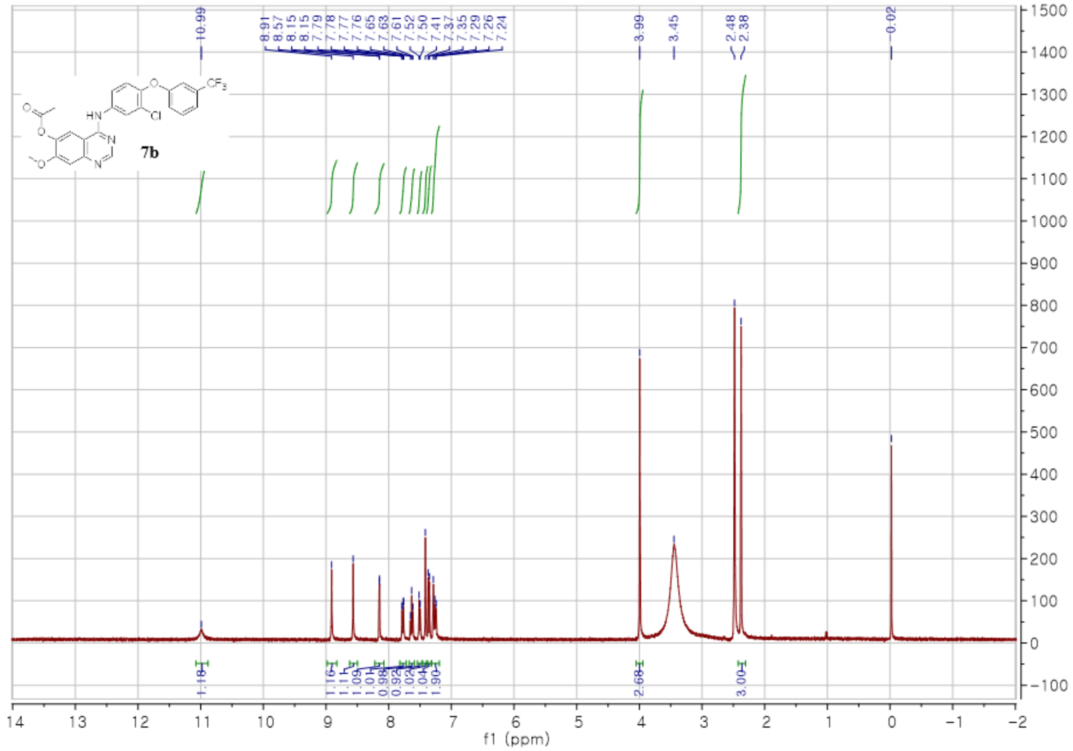

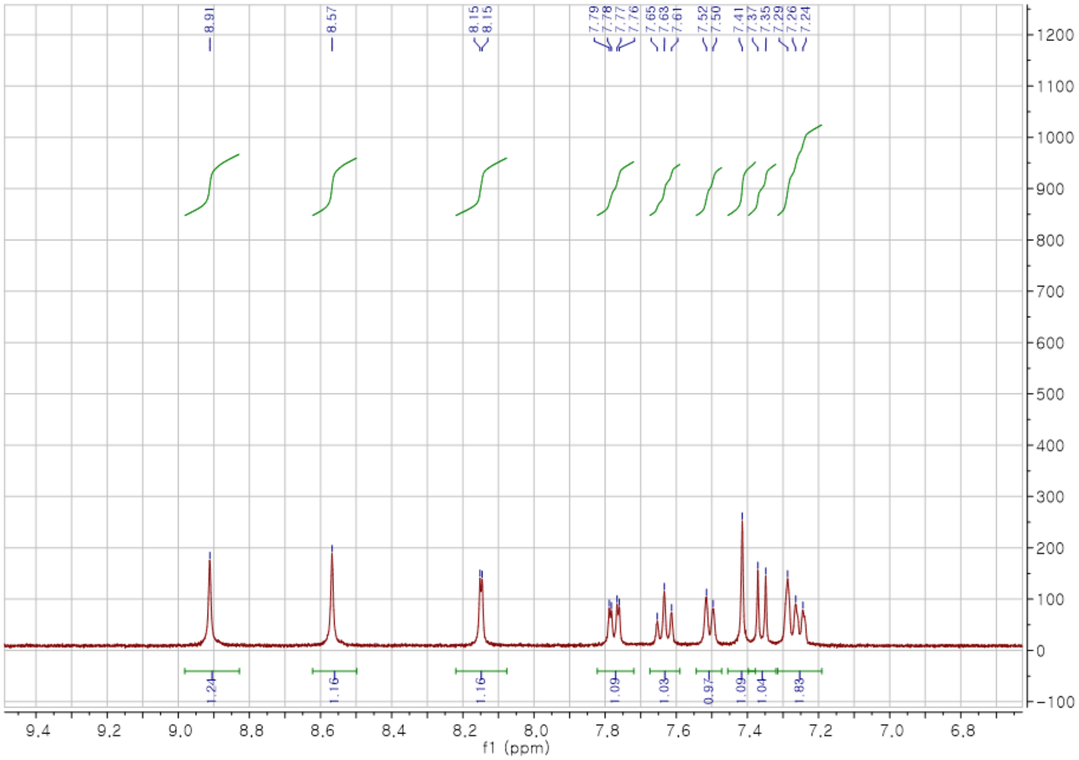

Figure SI 10. ^{1}H NMR spectrum of compound **8a**

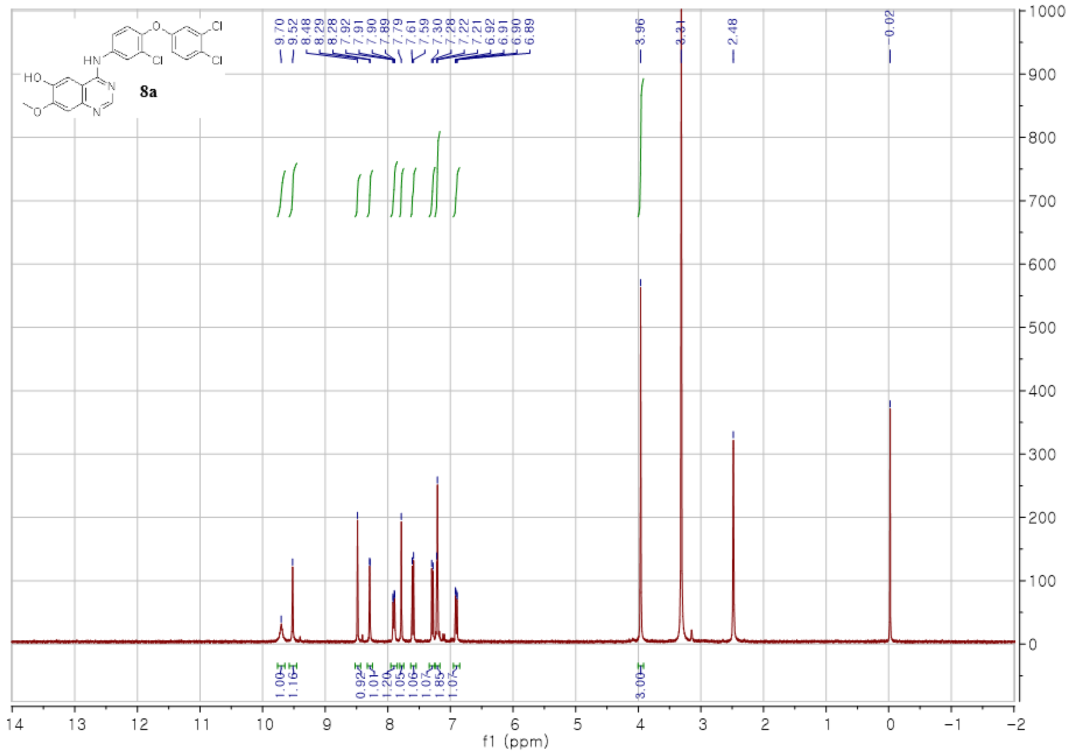

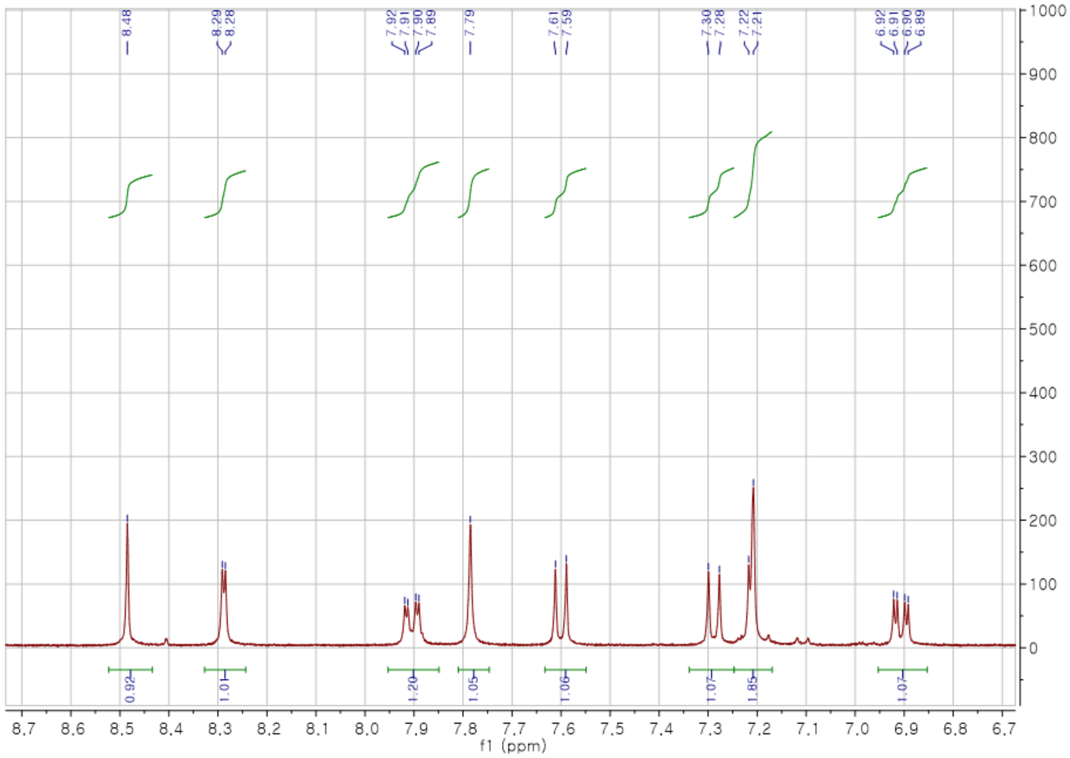

Figure SI 11. ^{13}C NMR spectrum of compound **8a**

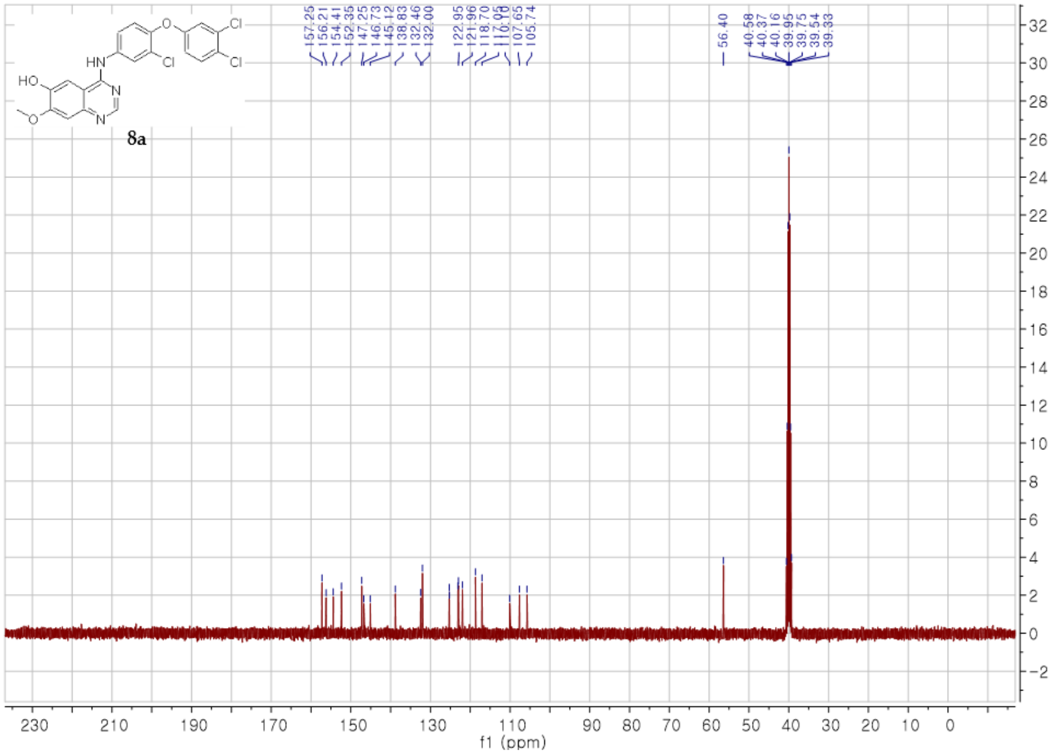

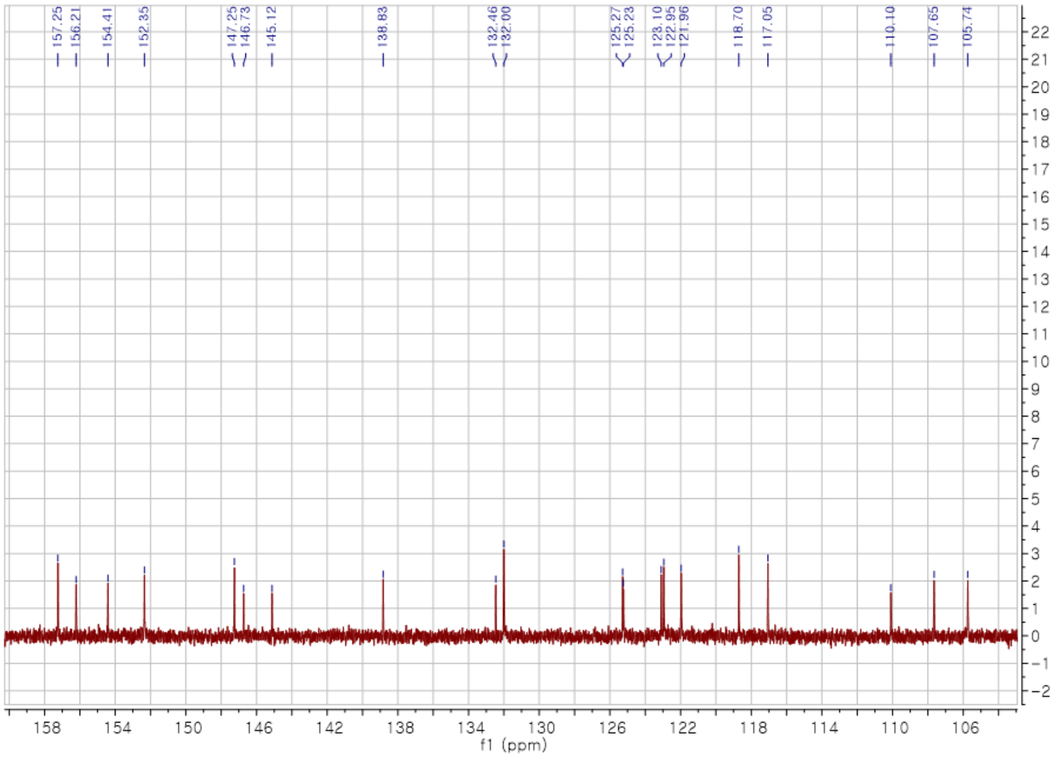

Figure SI 12. ^{1}H NMR spectrum of compound **8b**

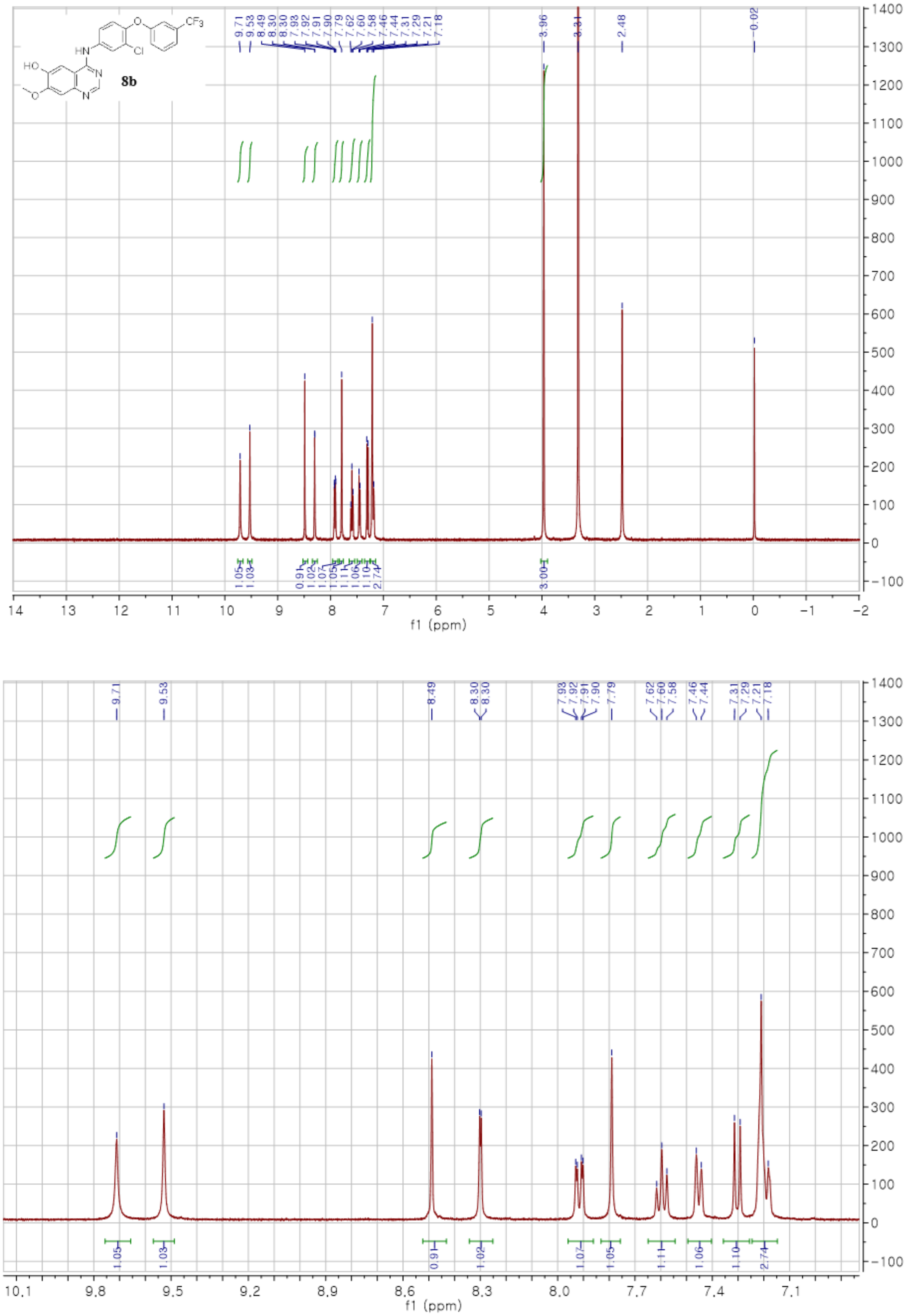

Figure SI 13. ^{13}C NMR spectrum of compound **8b**

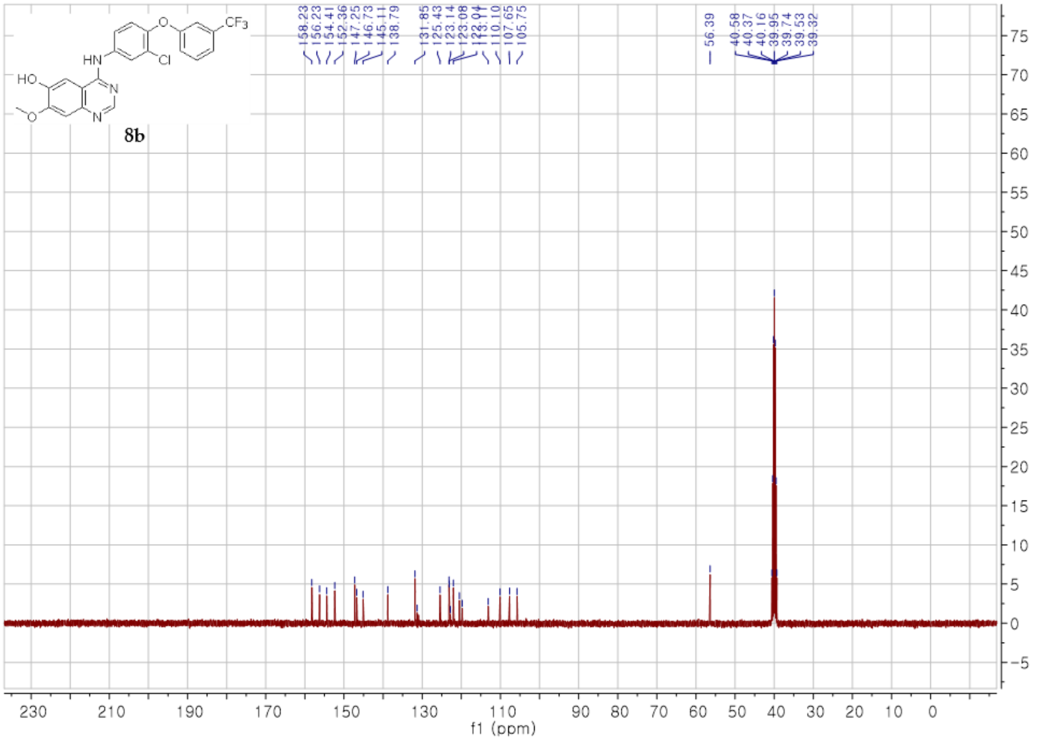

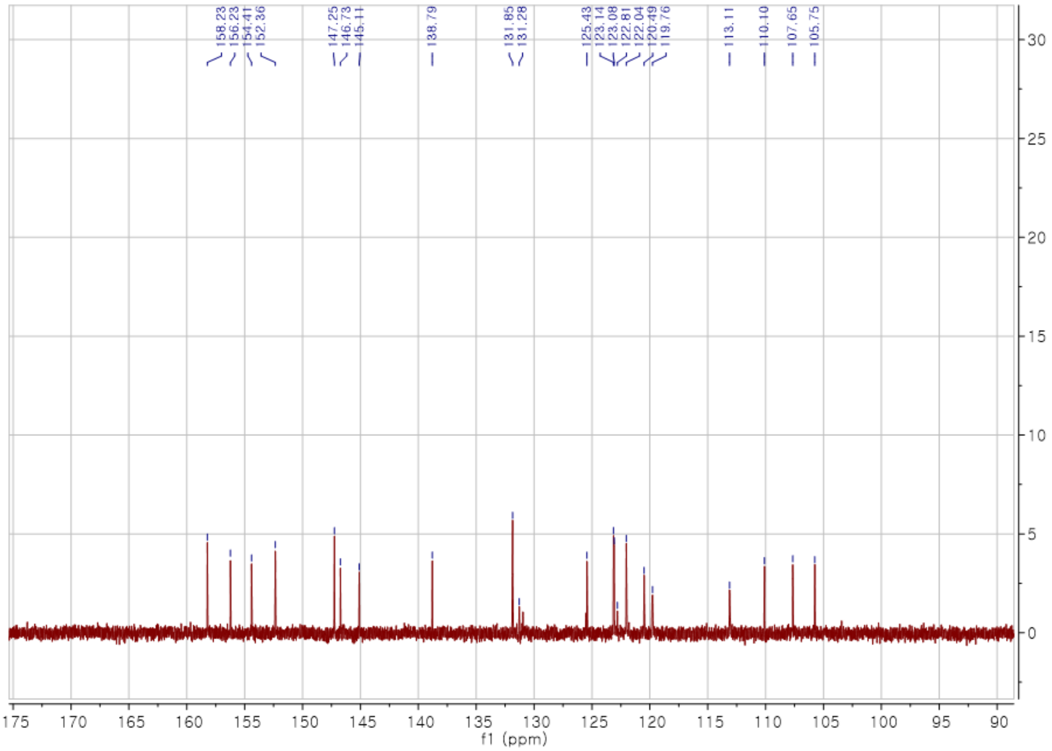

Figure SI 14. ^{1}H NMR spectrum of compound **9a**

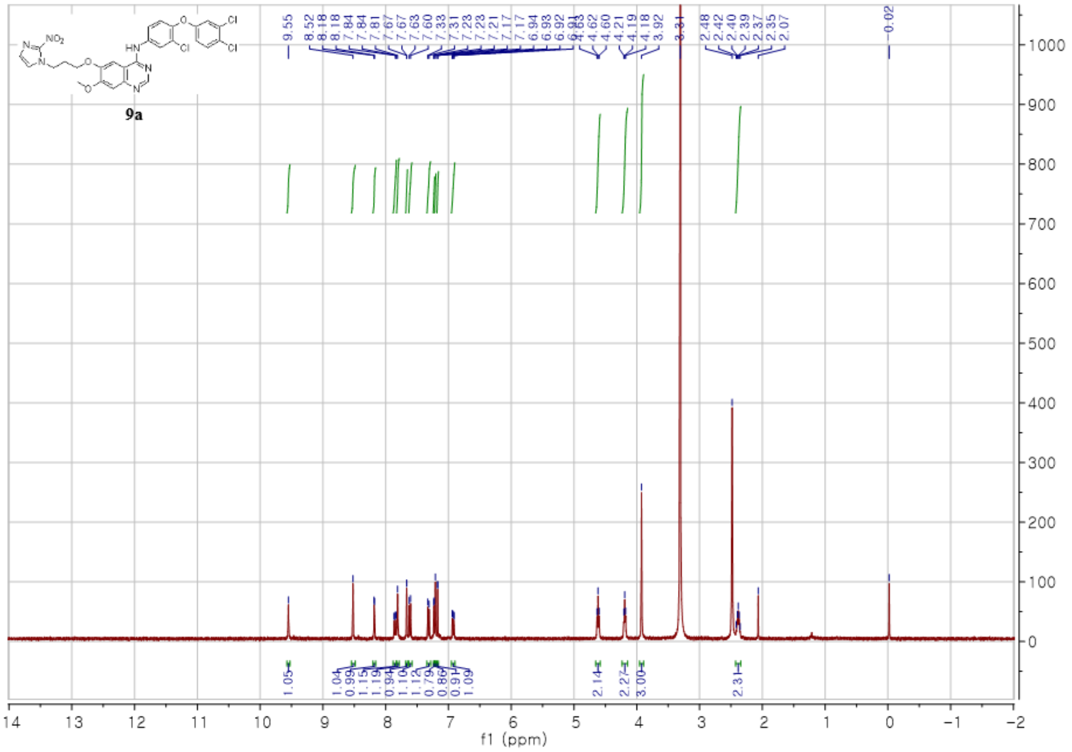

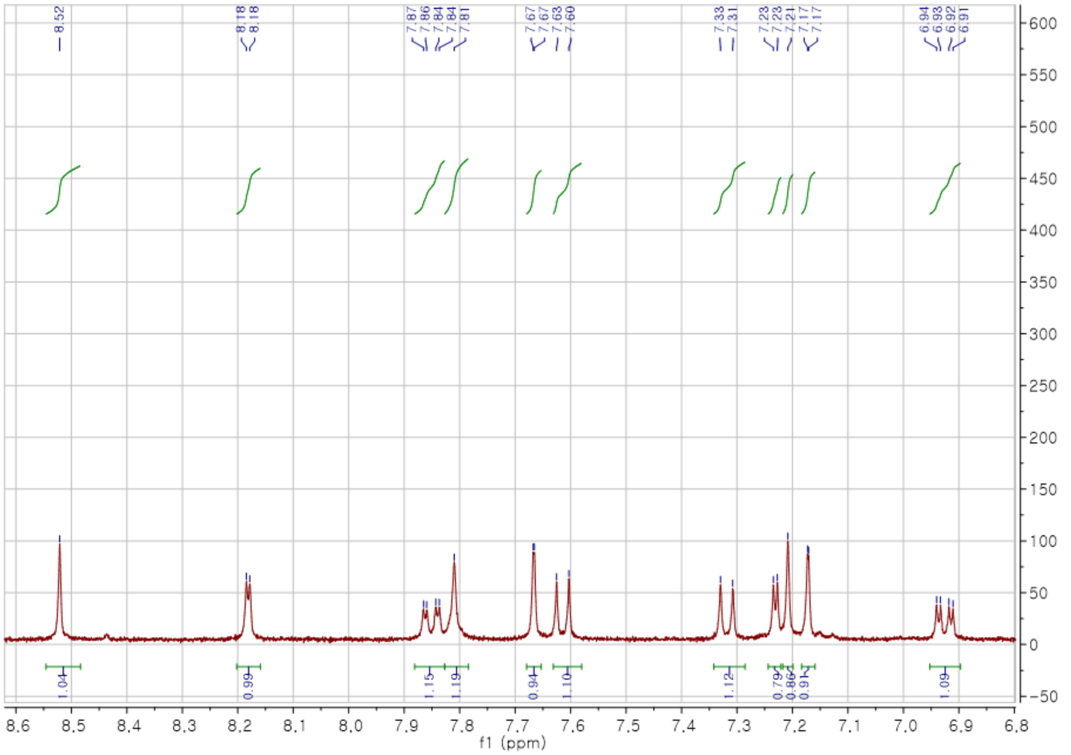

Figure SI 15. ^{13}C NMR spectrum of compound **9a**

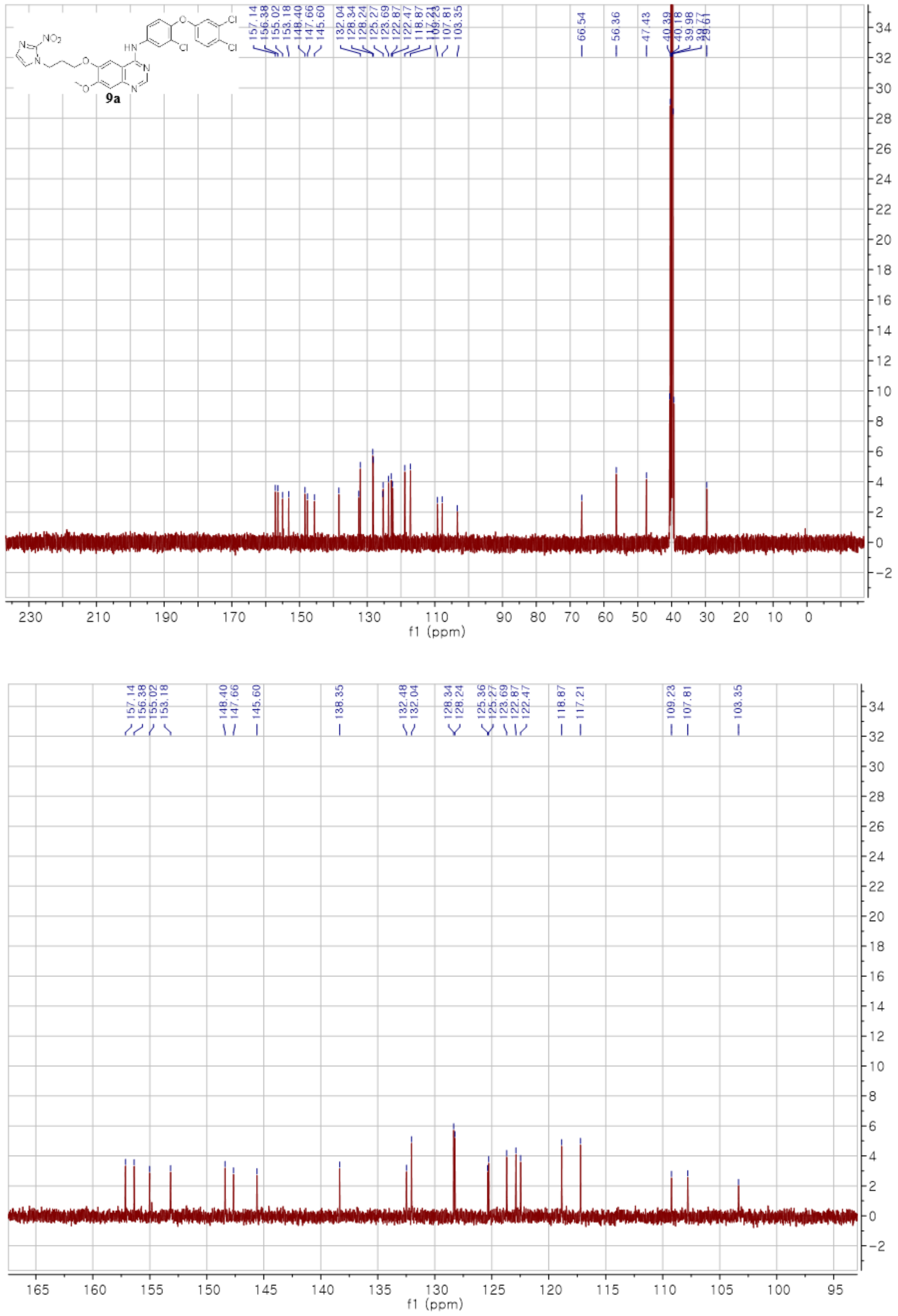

Figure SI 16. HRMS chart of compound **9a**

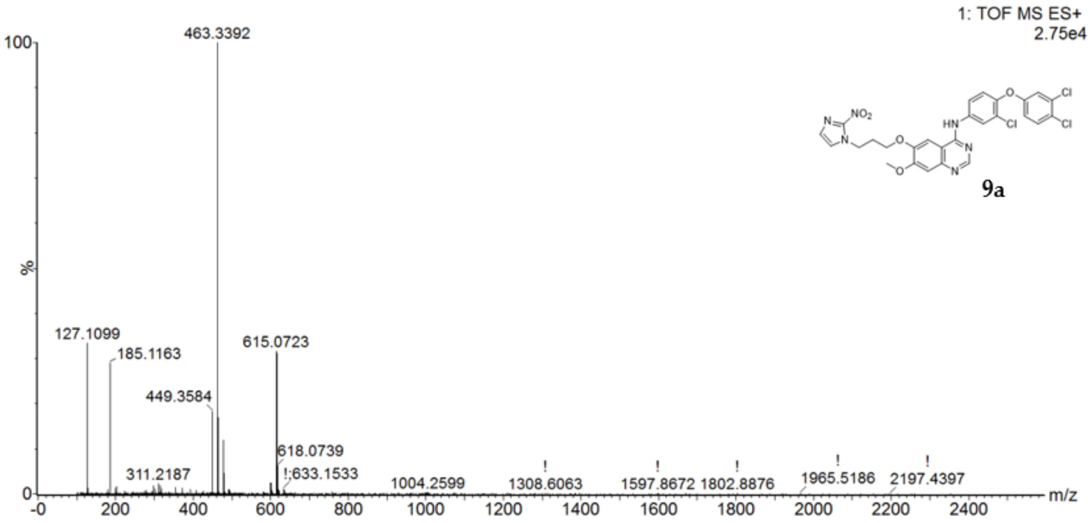

Figure SI 17. HPLC purity chart of compound **9a**

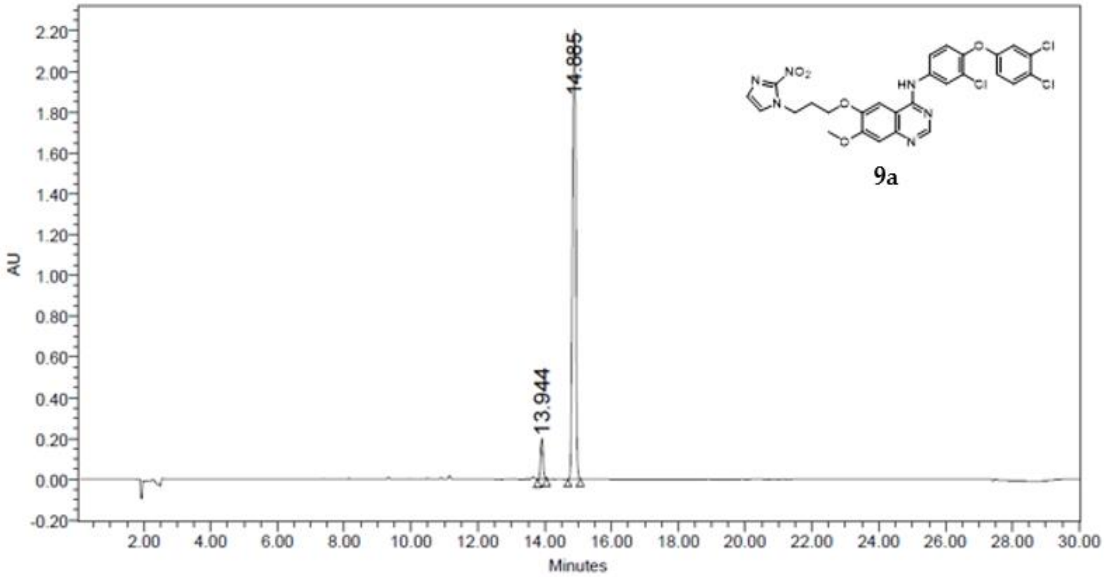

	RT	Area	% Area	Height
1	13.944	461545	3.05	207528
2	14.885	14670820	96.95	2207209

Figure SI 18. ^1H NMR spectrum of compound **9b**

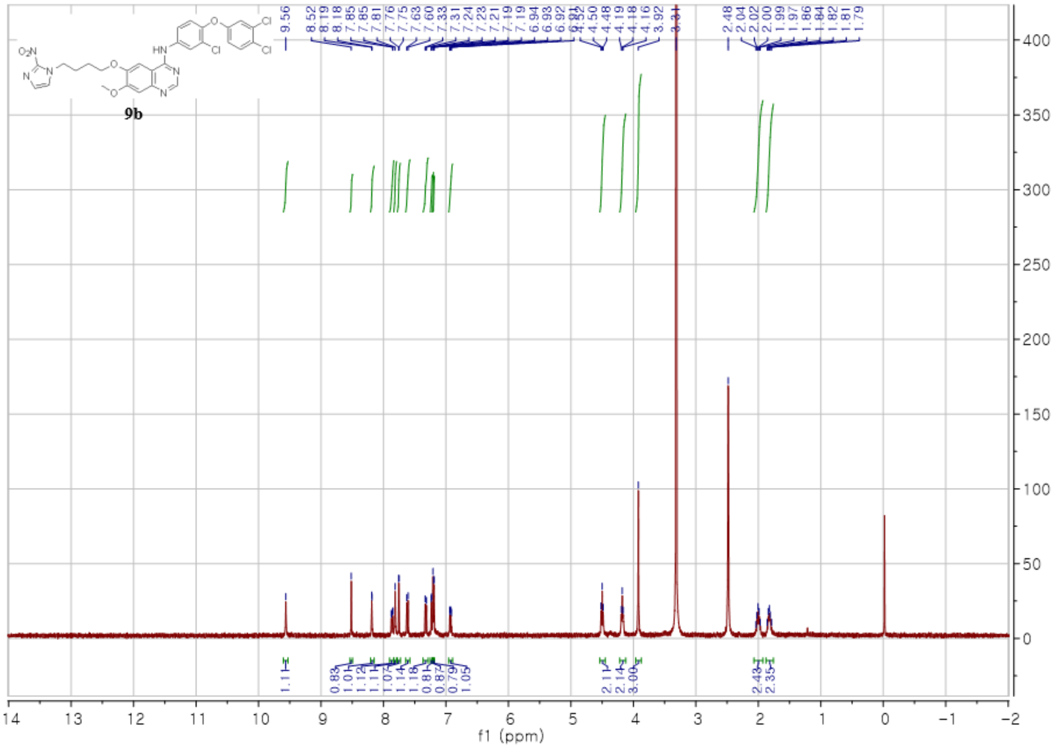

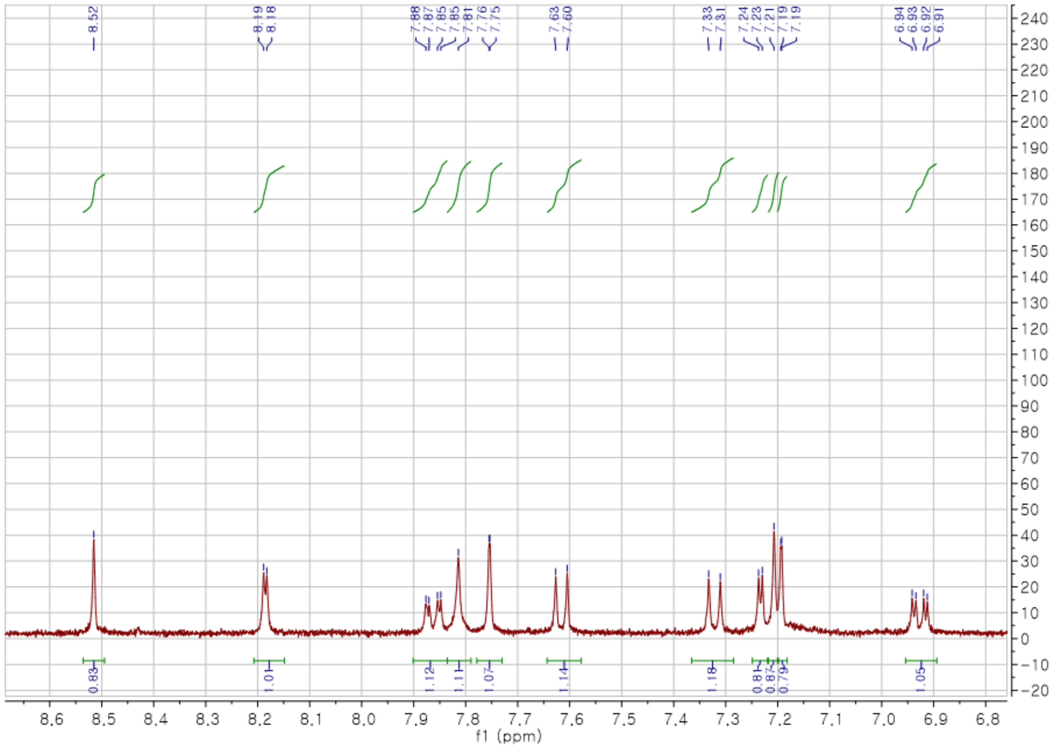

Figure SI 19. ^{13}C NMR spectrum of compound **9b**

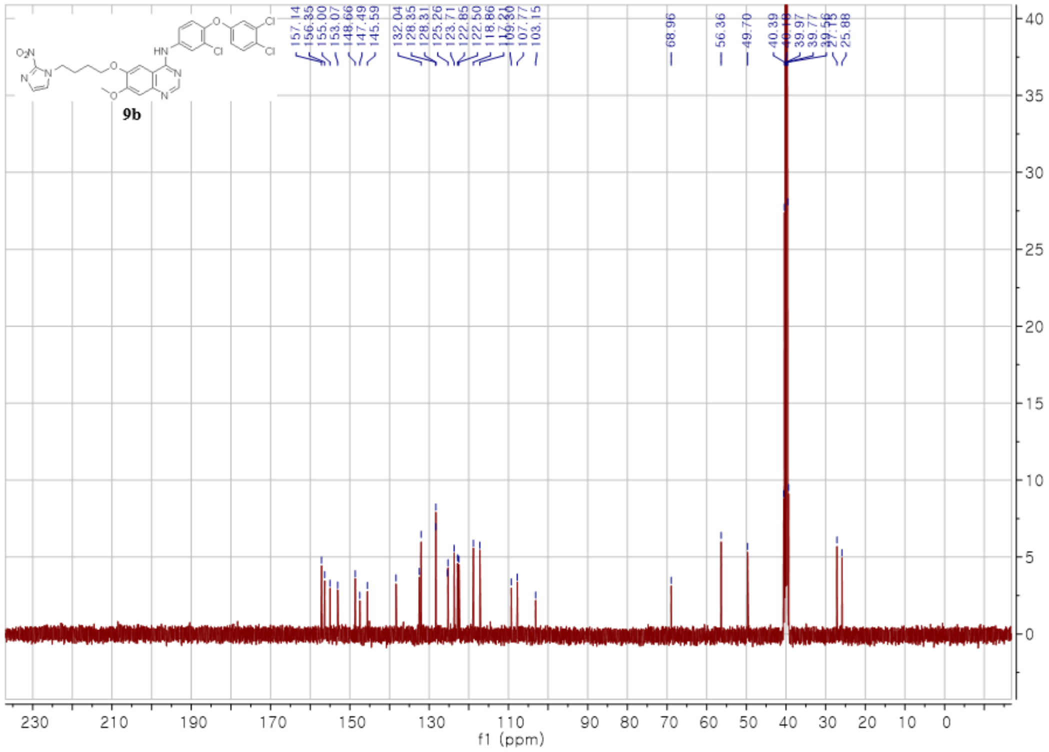

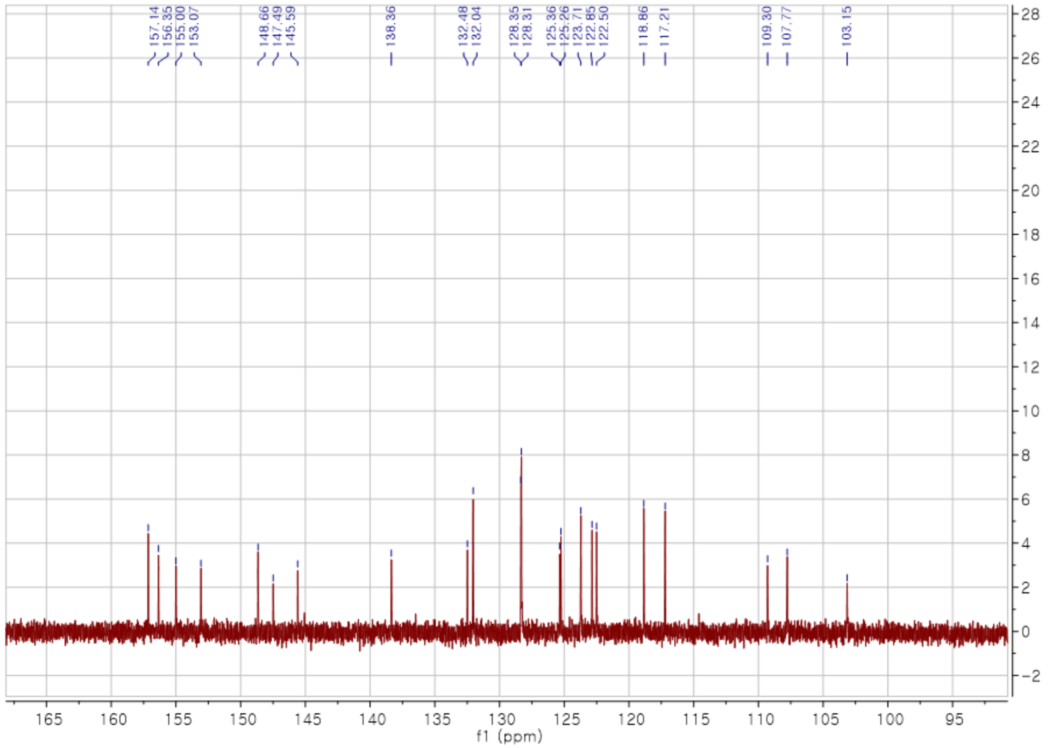

Figure SI 20. HRMS chart of compound **9b**

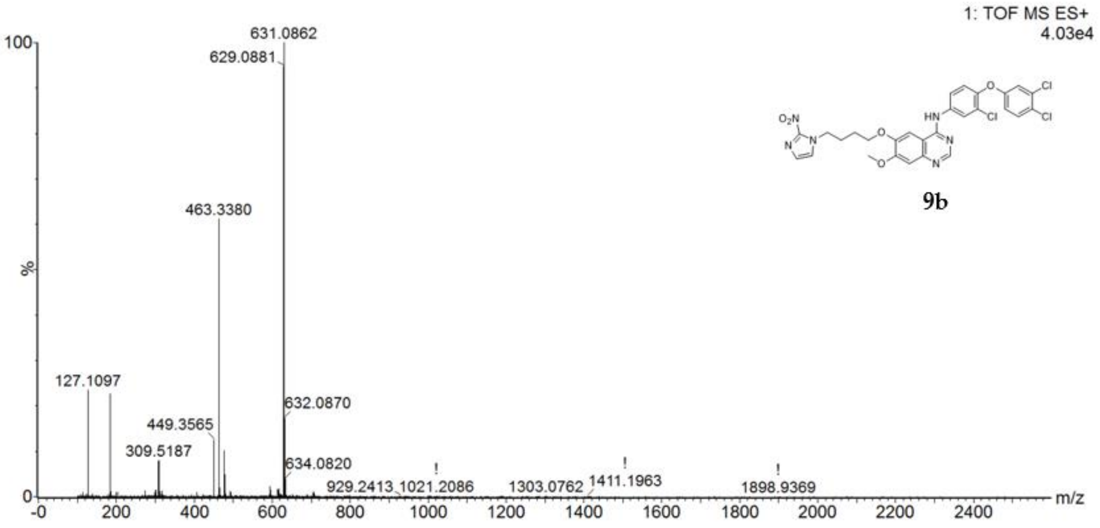

Figure SI 21. HPLC purity chart of compound **9b**

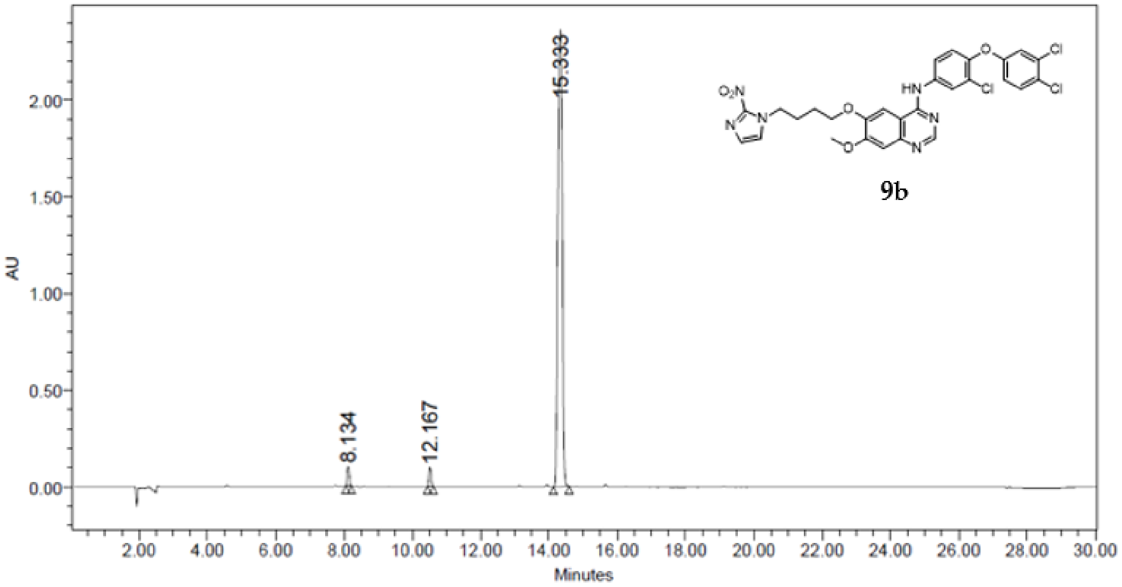

	RT	Area	% Area	Height
1	8.134	441783	1.92	105201
2	12.167	439482	1.91	101293
3	15.333	22128293	96.17	2392102

Figure SI 22. ^{1}H NMR spectrum of compound **9c**

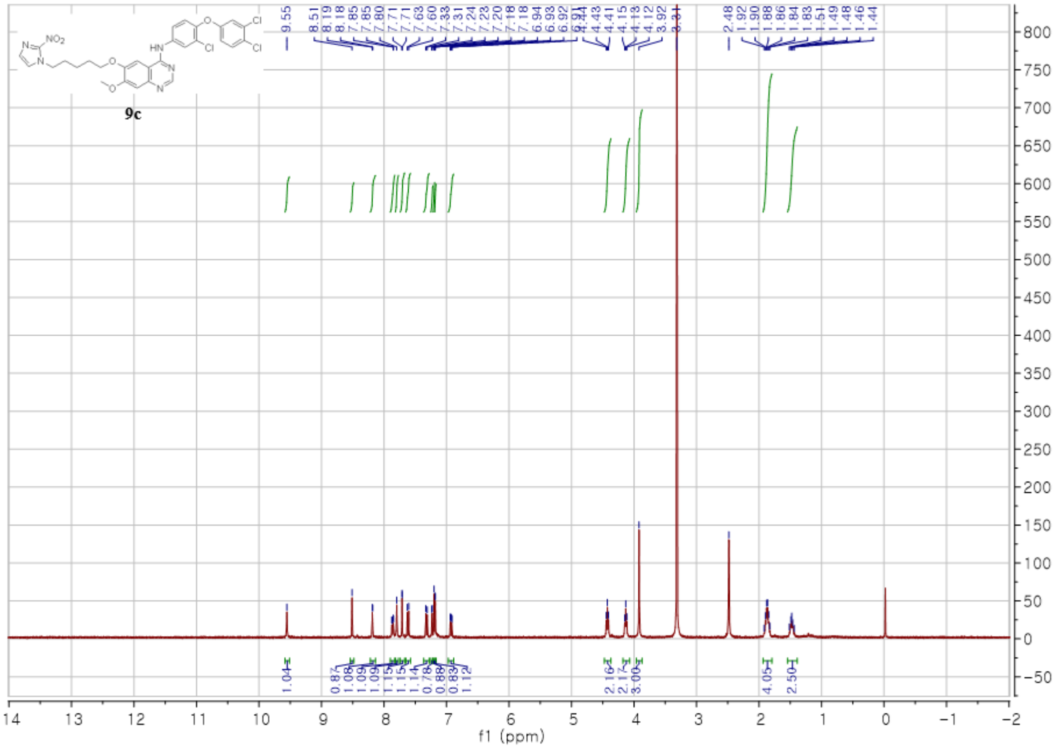

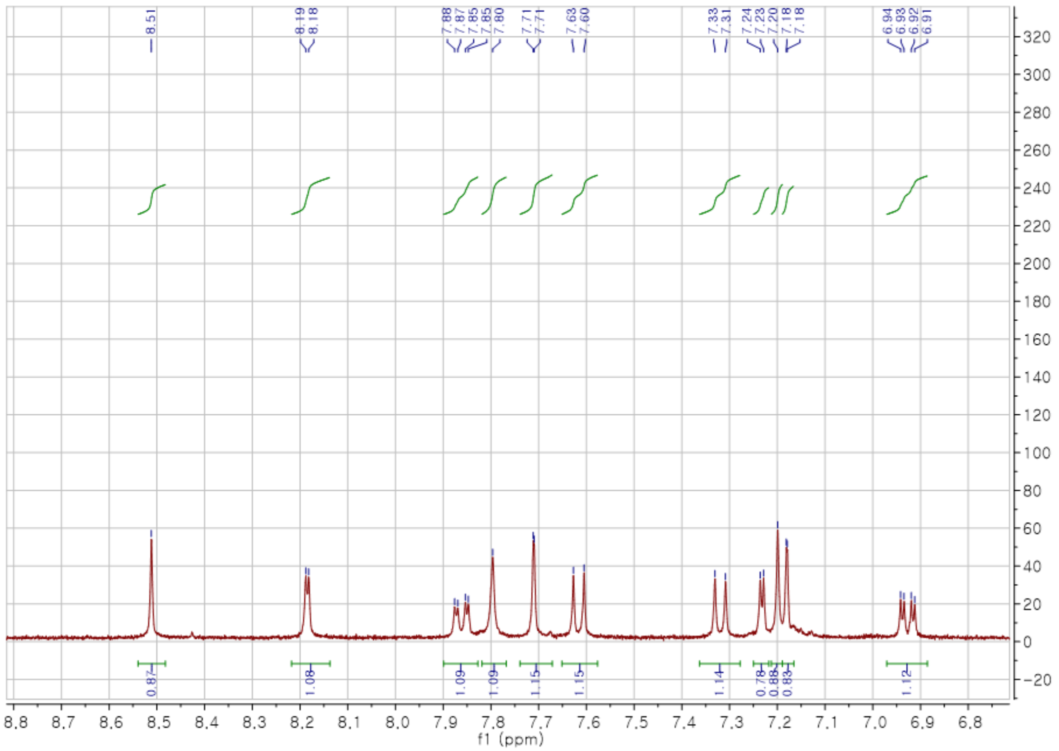

Figure SI 23. ^{13}C NMR spectrum of compound **9c**

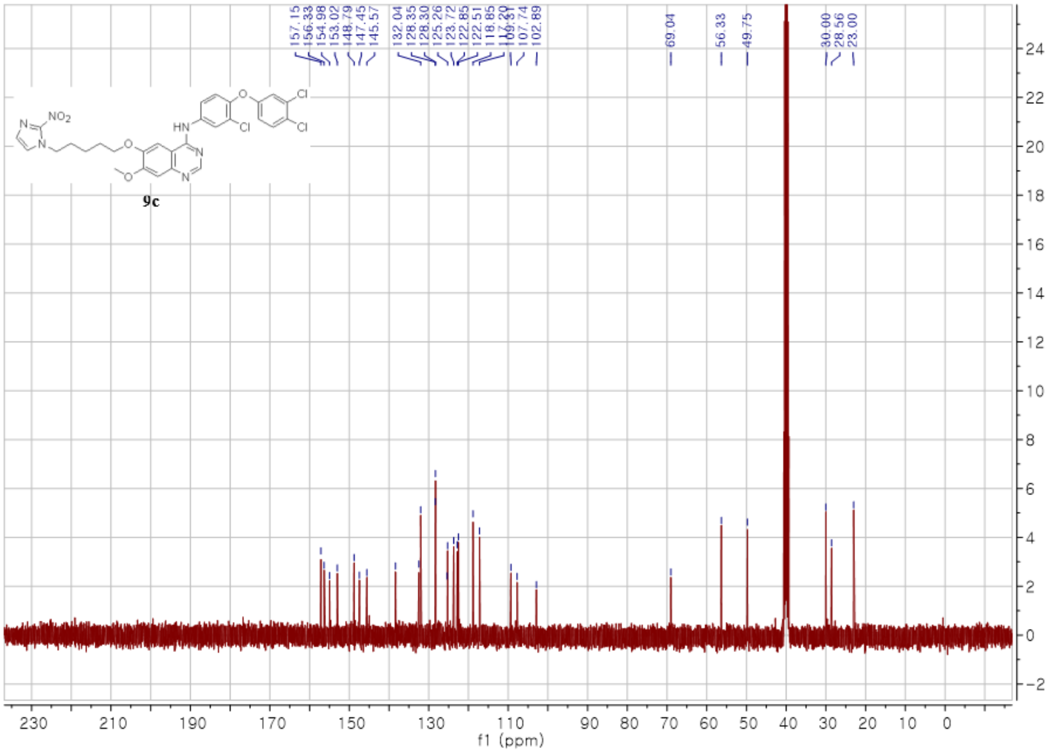

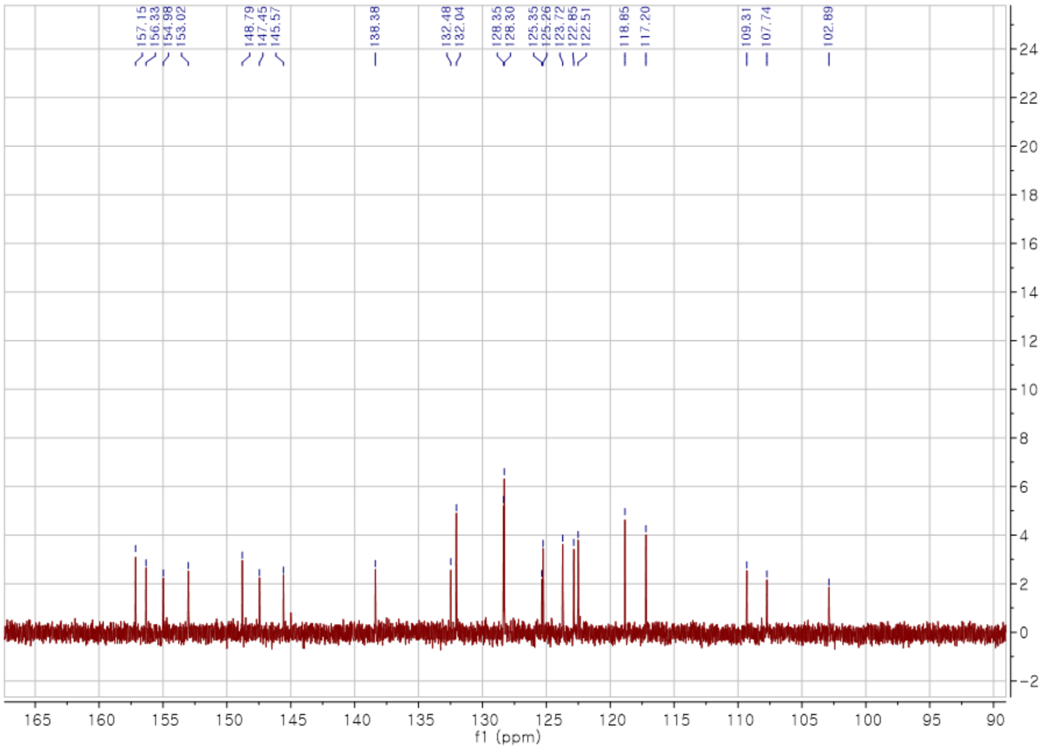

Figure SI 24. HRMS chart of compound **9c**

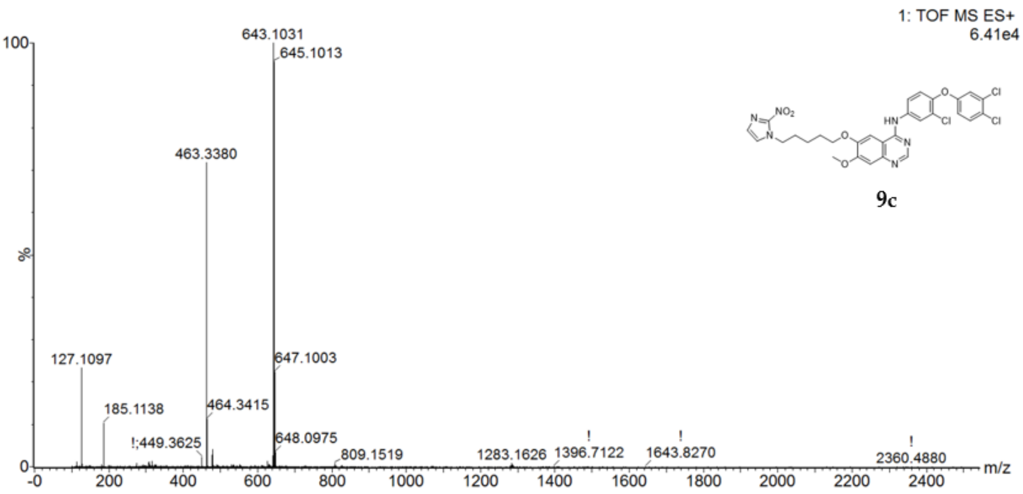

Figure SI 25. HPLC purity chart of compound **9c**

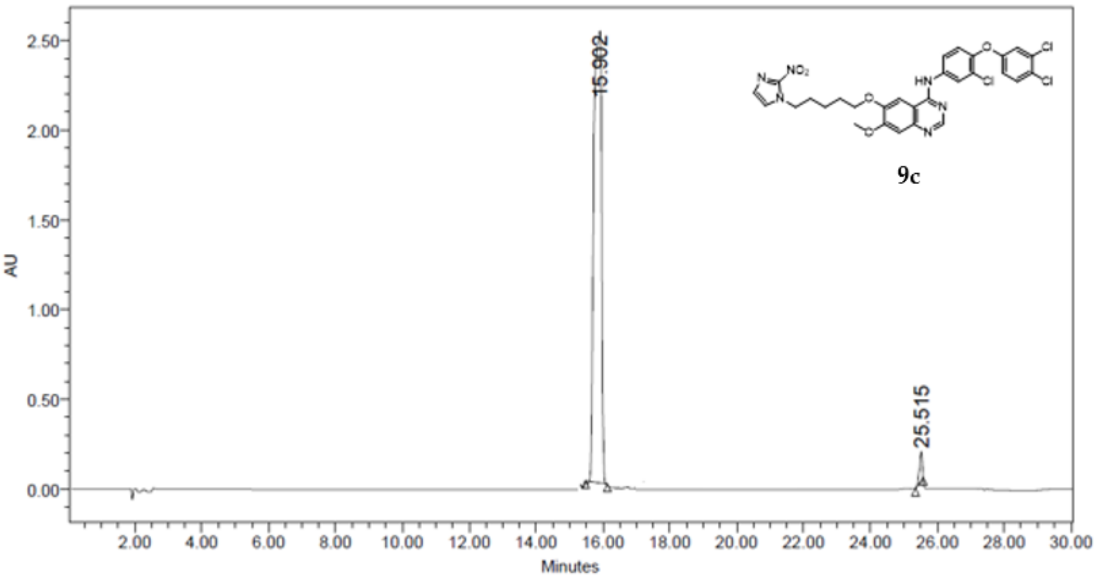

	RT	Area	% Area	Height
1	15.902	44524928	97.95	2545600
2	25.515	931731	2.05	162474

Figure SI 26. ^{1}H NMR spectrum of compound **9d**

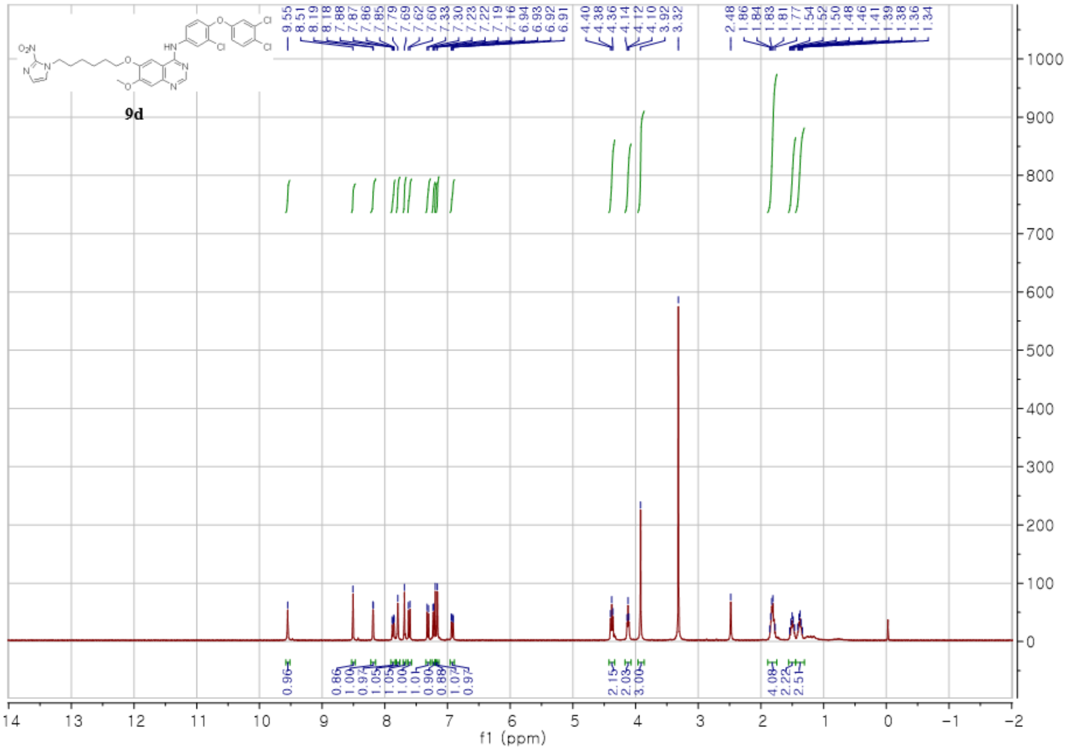

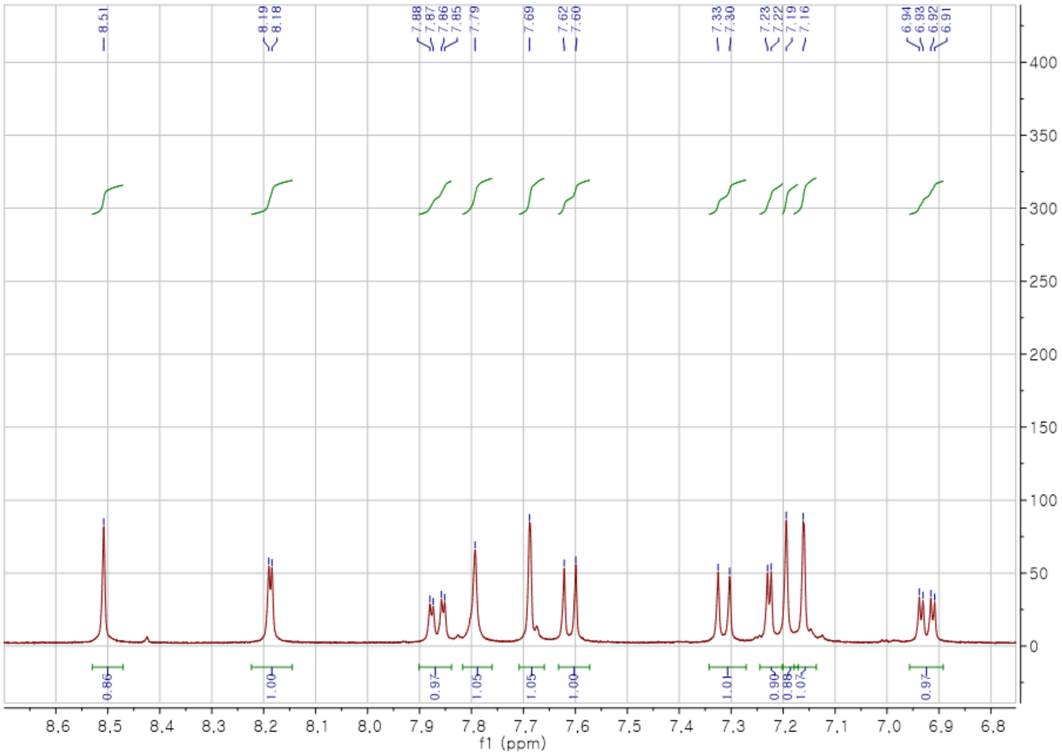

Figure SI 27. ^{13}C NMR spectrum of compound **9d**

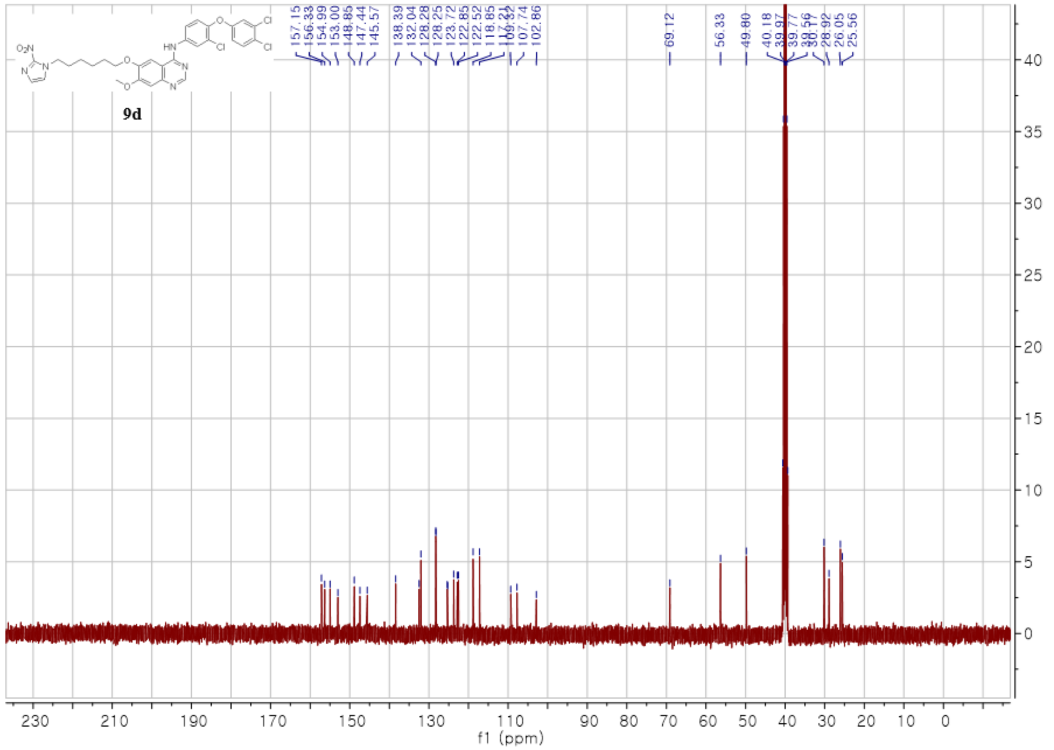

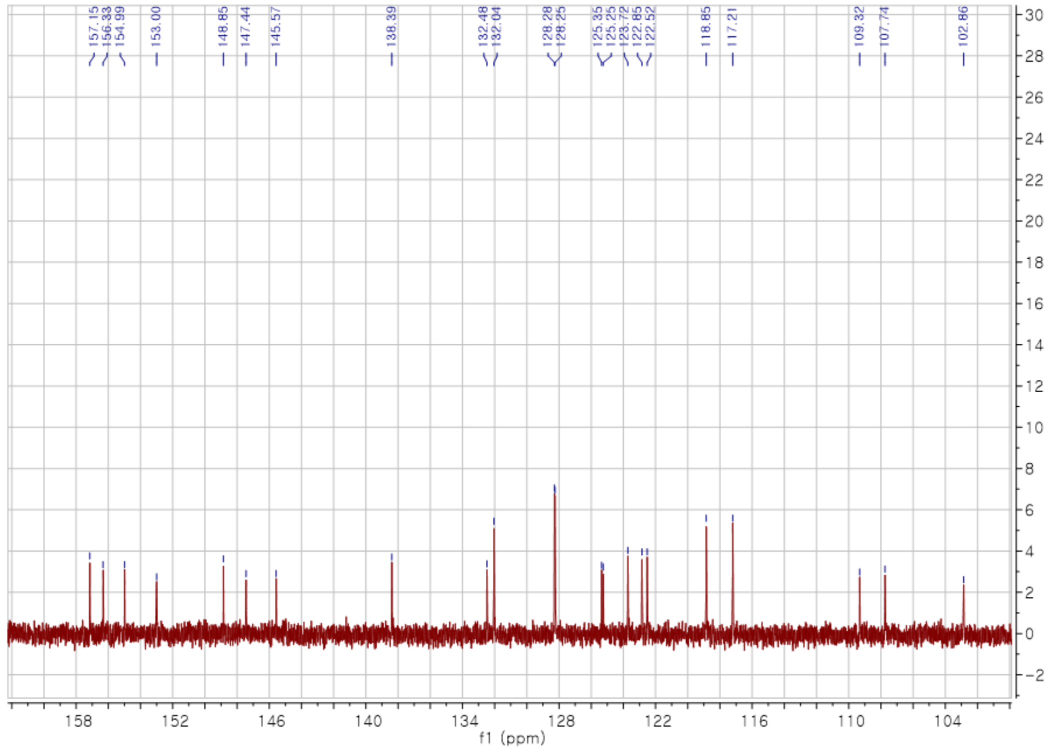

Figure SI 28. HRMS chart of compound **9d**

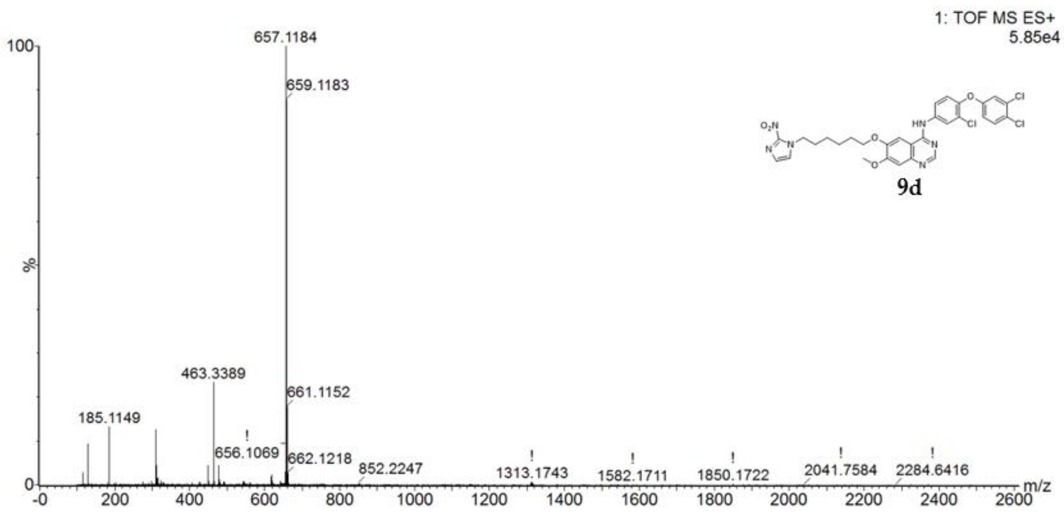

Figure SI 29. HPLC purity chart of compound **9d**

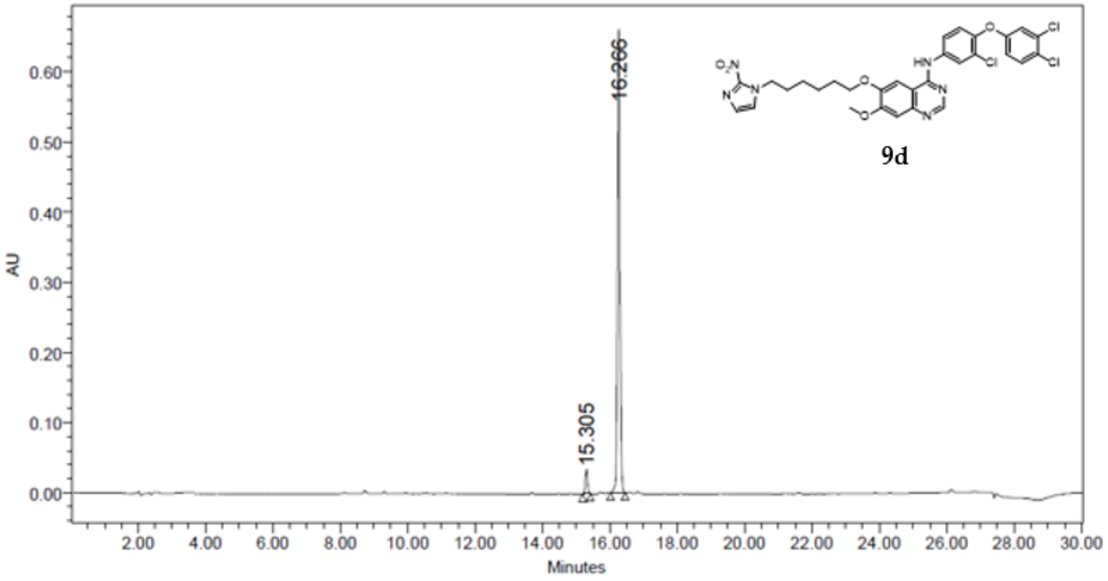

	RT	Area	% Area	Height
1	15.305	166653	4.69	34129
2	16.266	3384269	95.31	653605

Figure SI 30. ^{1}H NMR spectrum of compound **9e**

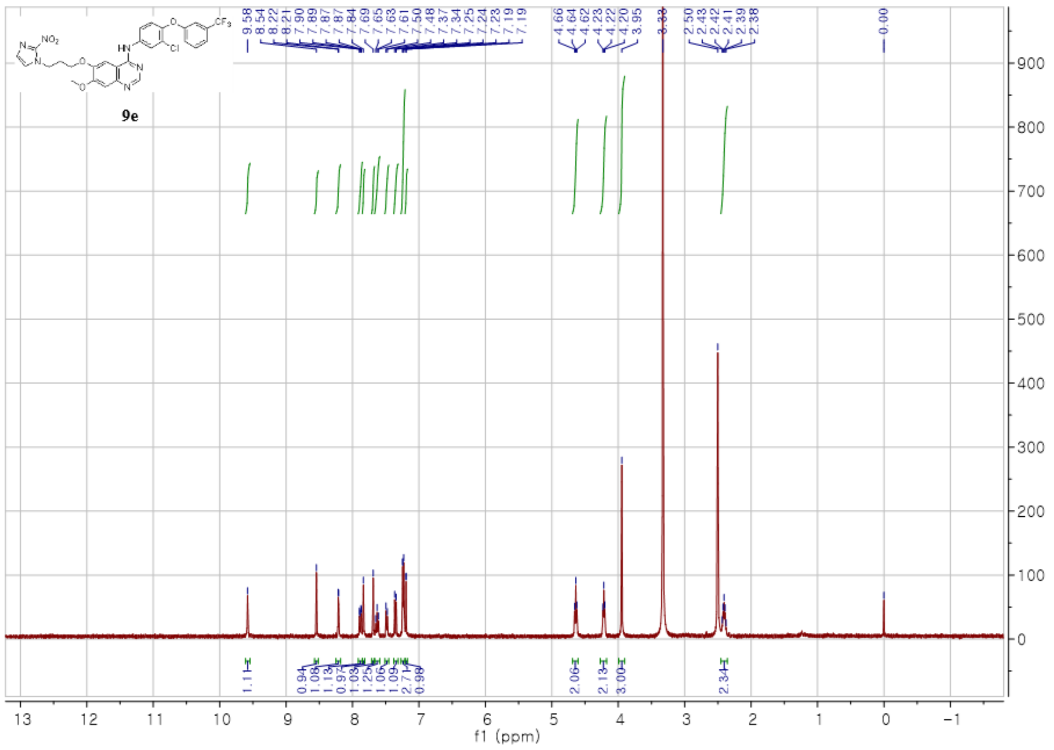

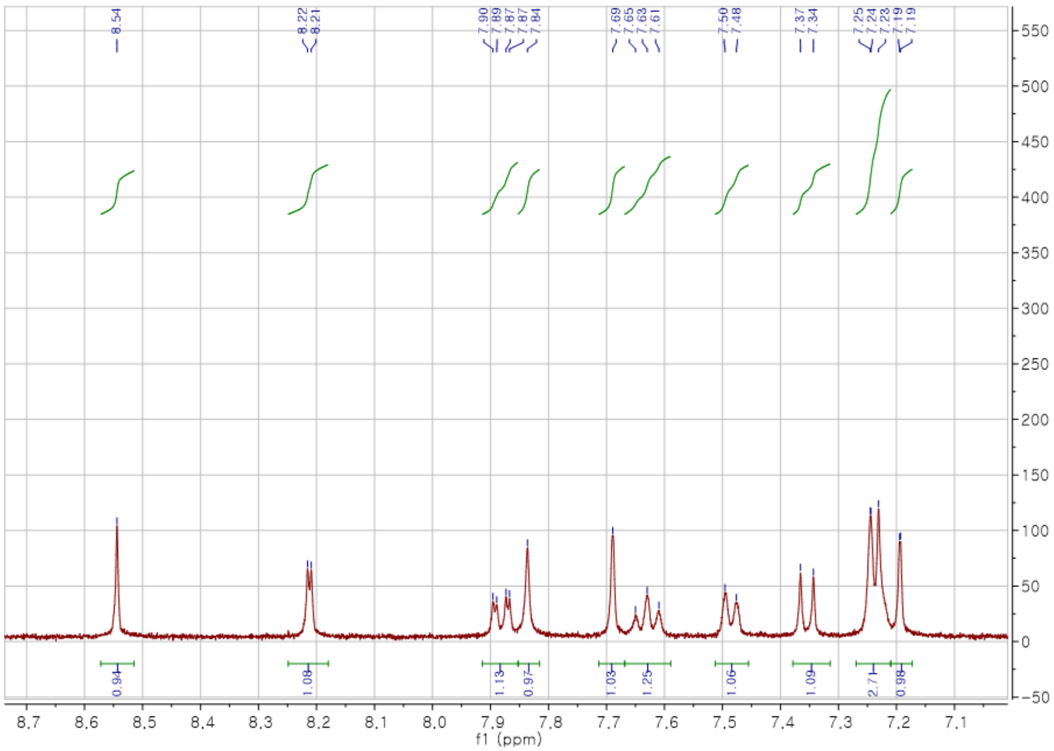

Figure SI 31. ^{13}C NMR spectrum of compound **9e**

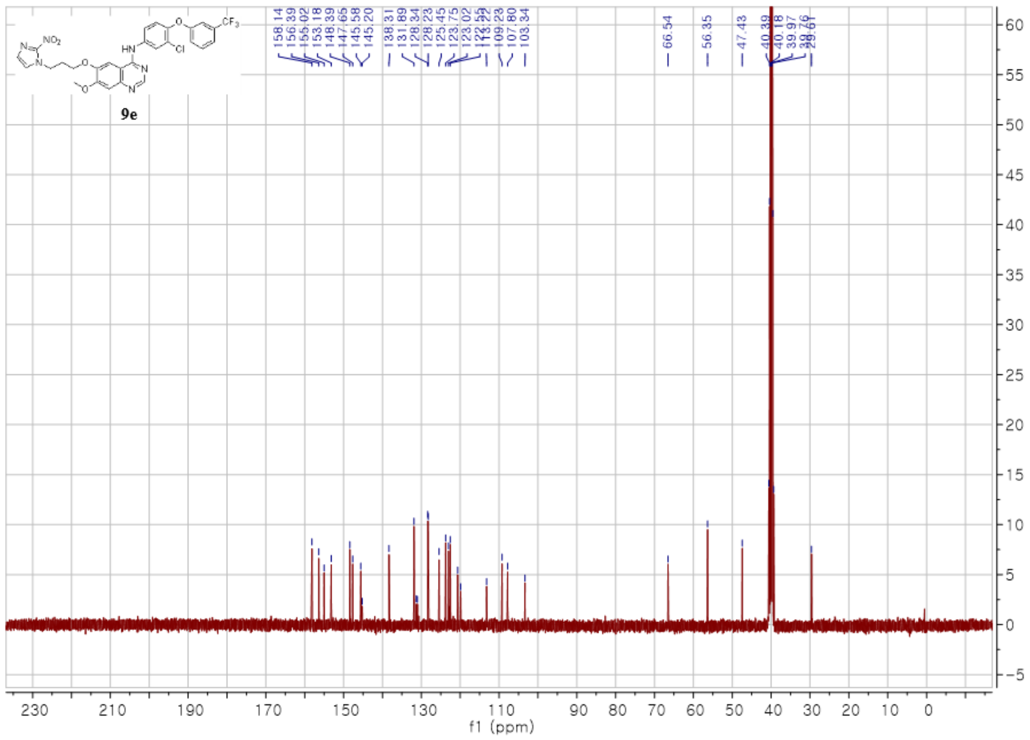

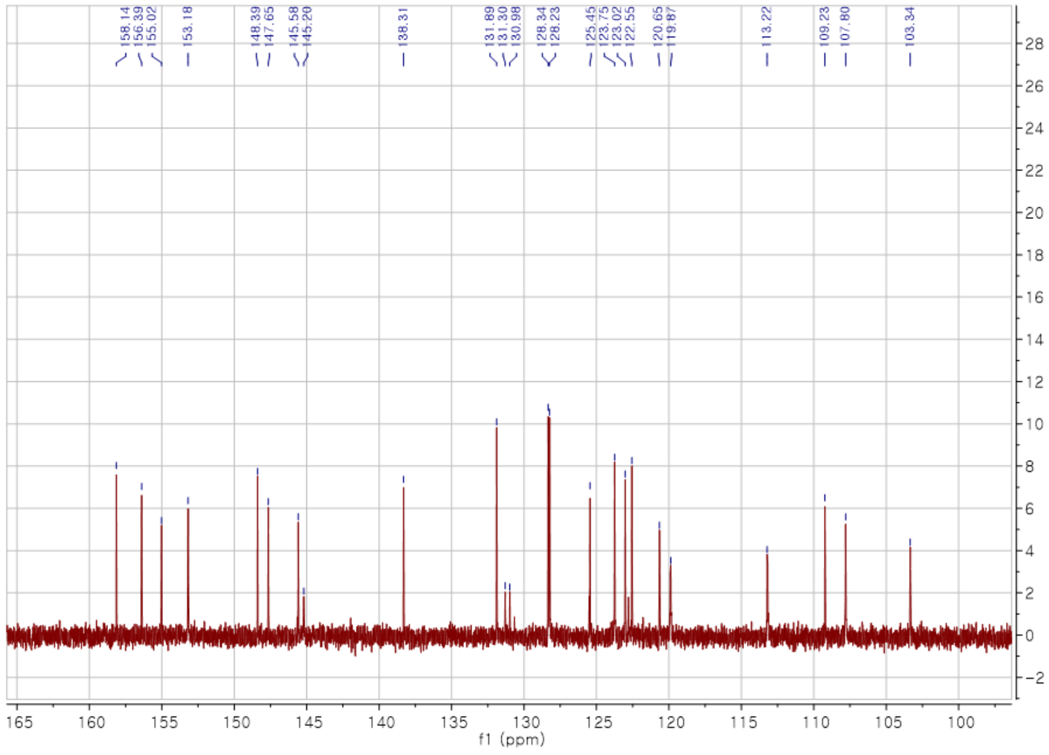

Figure SI 32. HRMS chart of compound **9e**

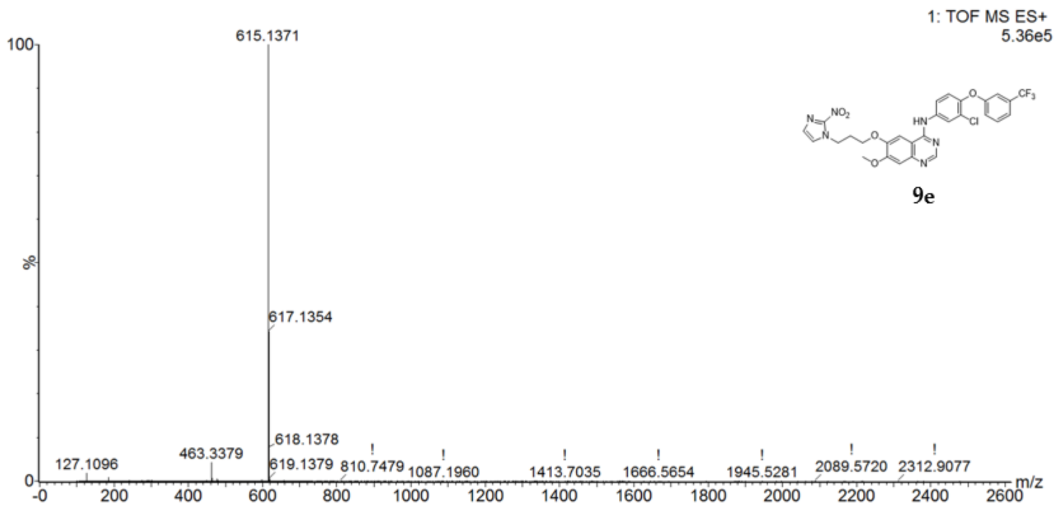

Figure SI 33. HPLC purity chart of compound **9e**

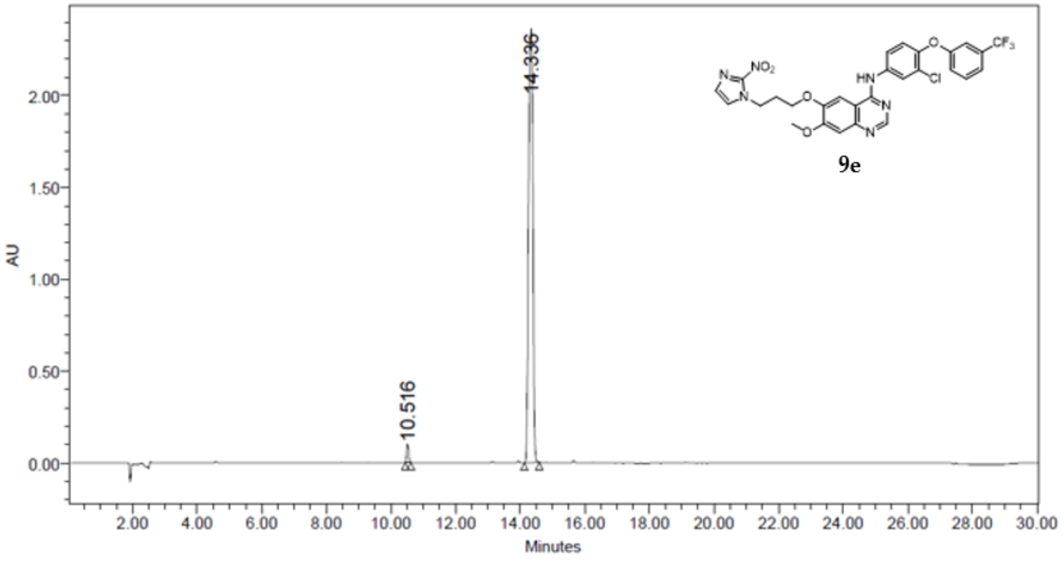

	RT	Area	% Area	Height
1	10.516	432214	1.92	98965
2	14.336	22056246	98.08	2390526

Figure SI 34. ^{1}H NMR spectrum of compound **9f**

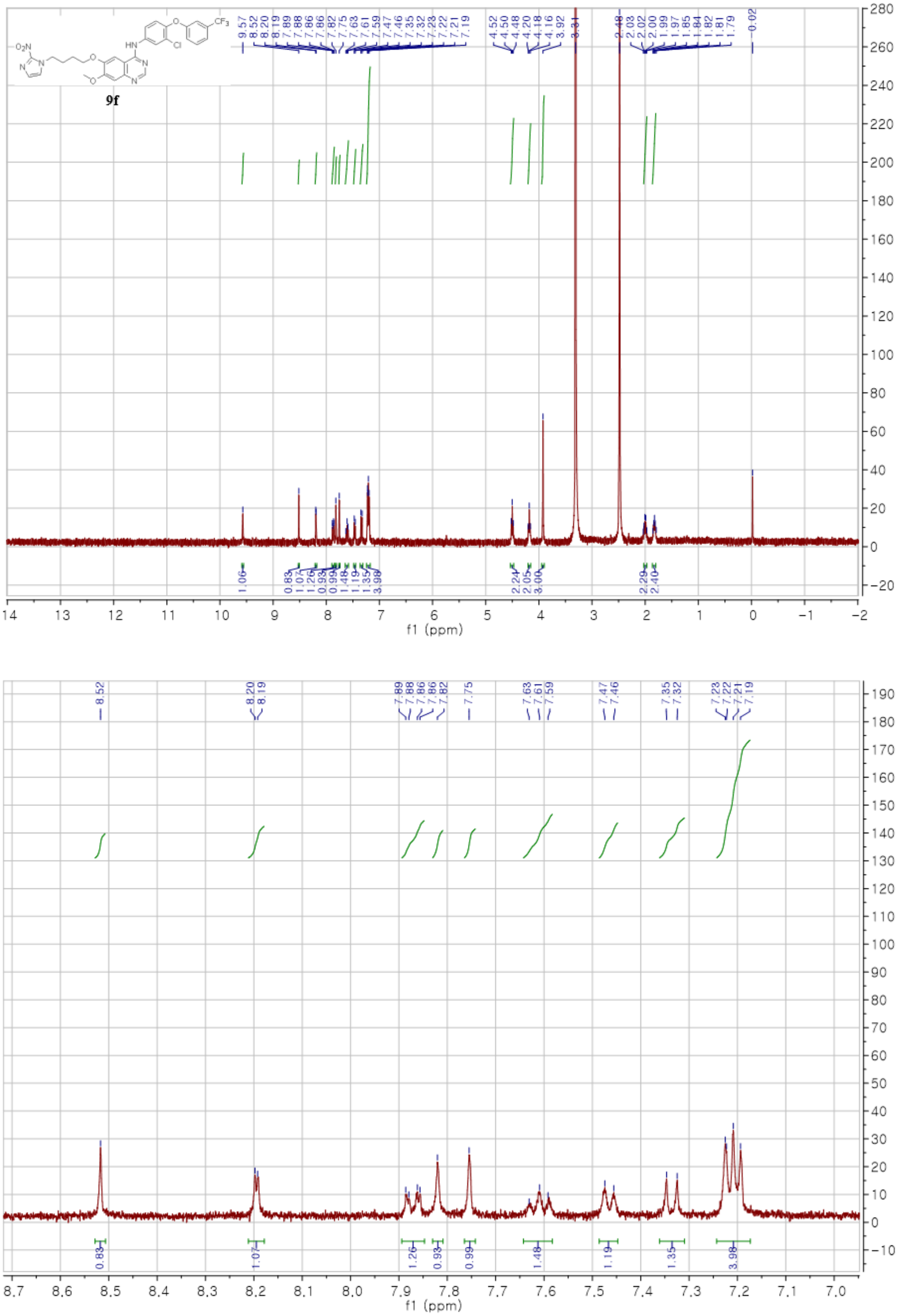

Figure SI 35. ¹³C NMR spectrum of compound **9f**

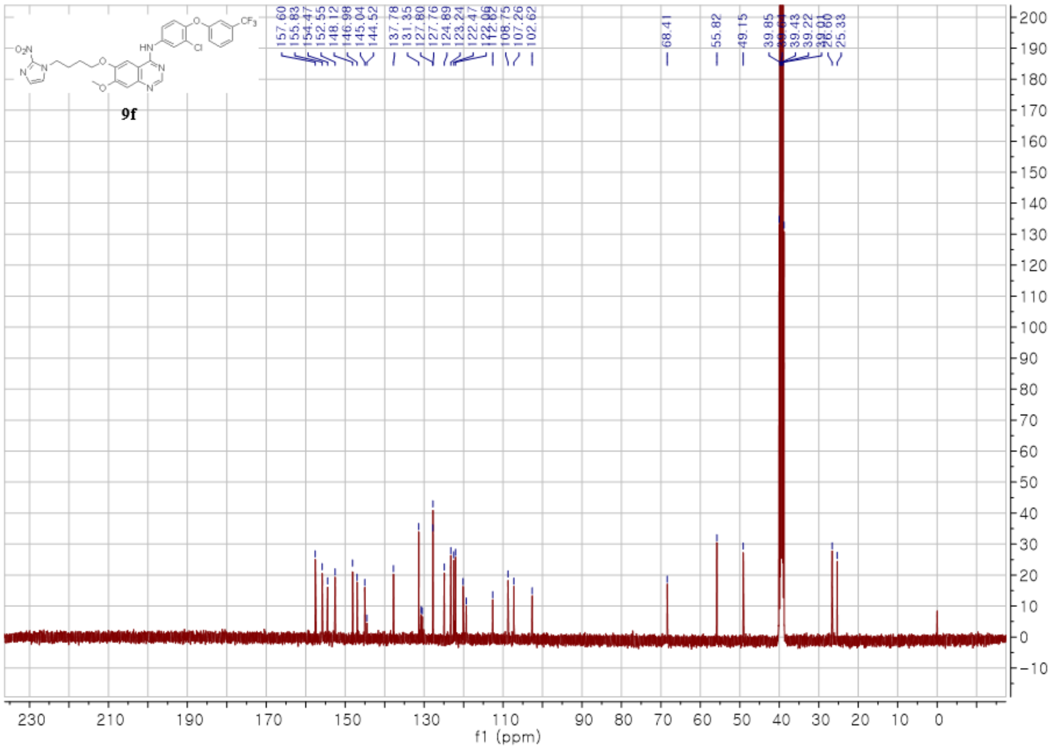

9f

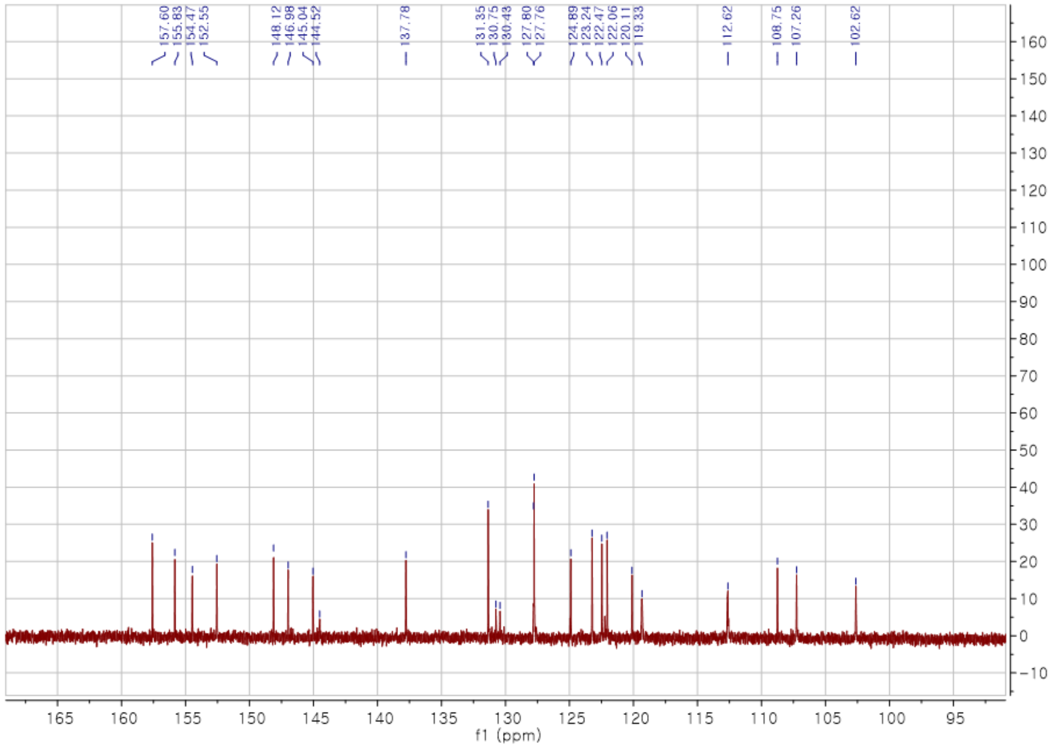

Figure SI 36. HRMS chart of compound **9f**

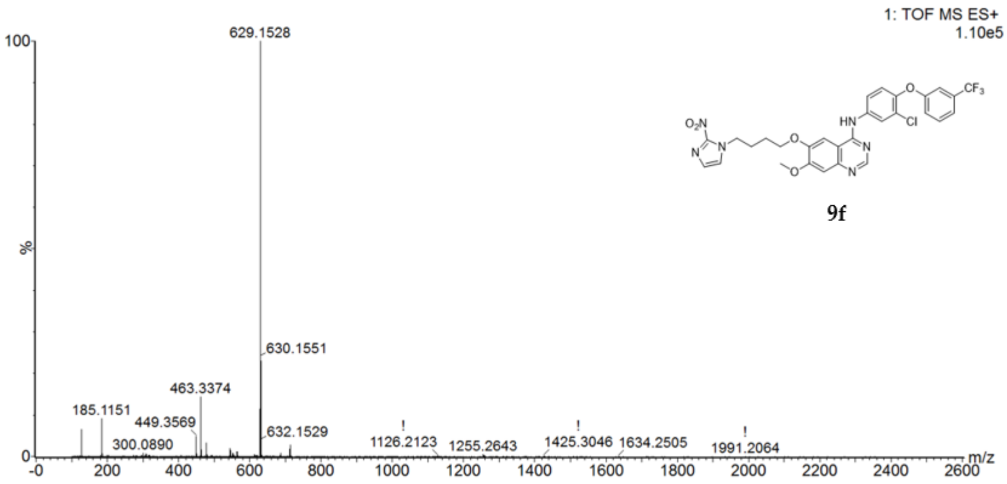

Figure SI 37. HPLC purity chart of compound **9f**

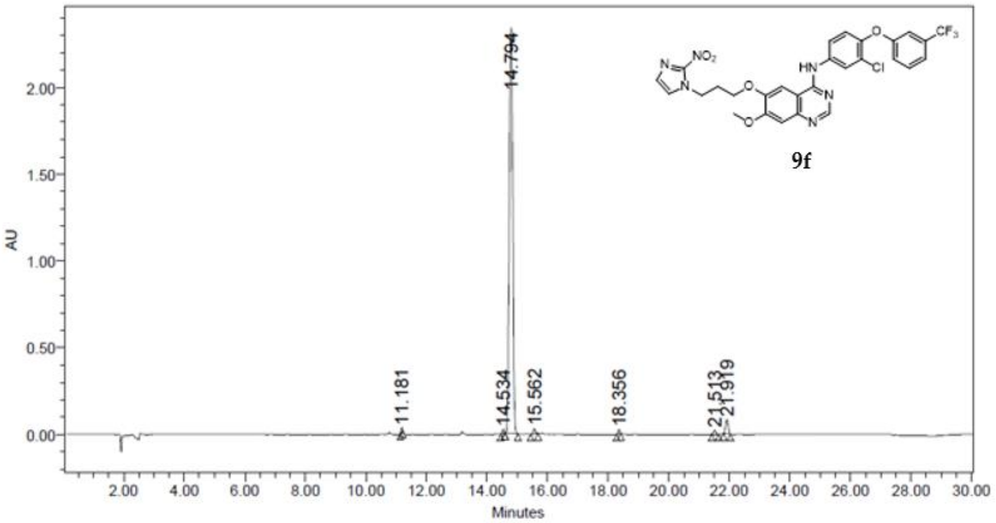

	RT	Area	% Area	Height
1	11.181	70259	0.35	23097
2	14.534	79720	0.40	20813
3	14.794	18994297	95.07	2353810
4	15.562	121467	0.61	27583
5	18.356	97936	0.49	22156
6	21.513	132728	0.66	22843
7	21.919	482602	2.42	80805

Figure SI 38. ^{1}H NMR spectrum of compound **9g**

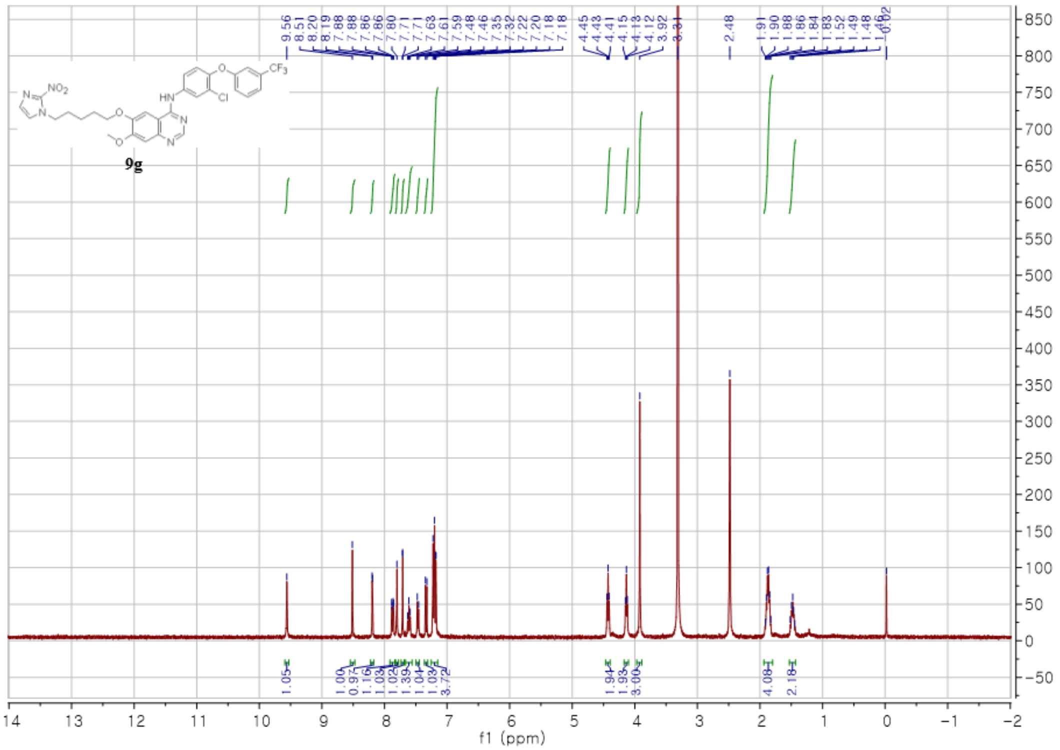

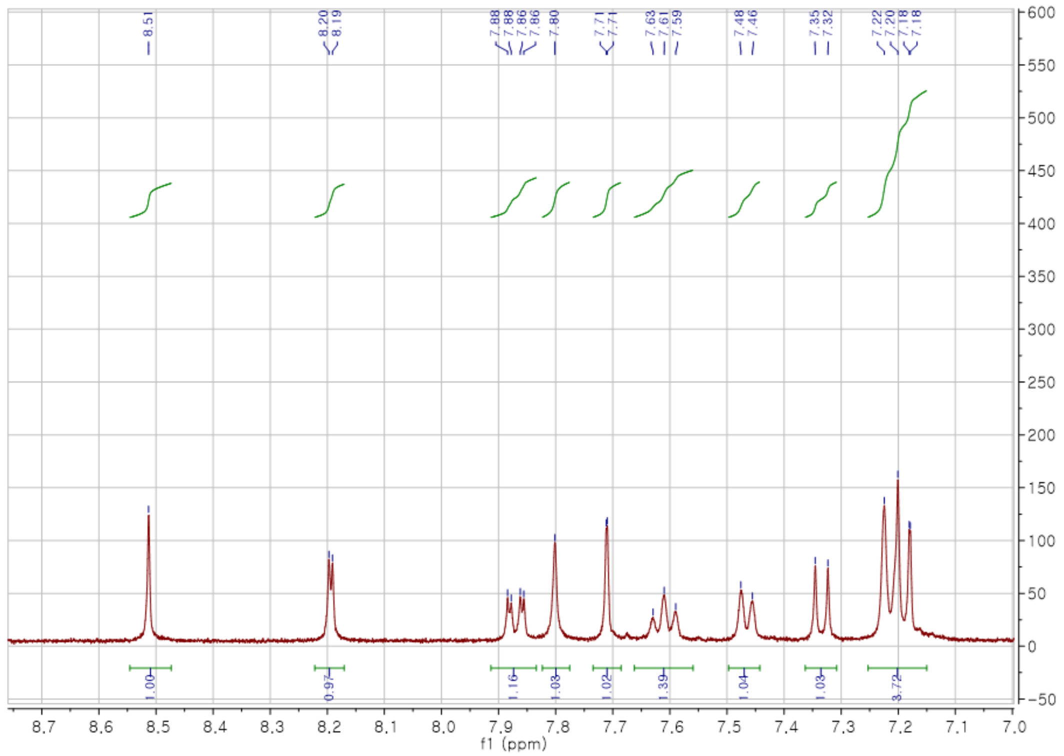

Figure SI 39. ^{13}C NMR spectrum of compound **9g**

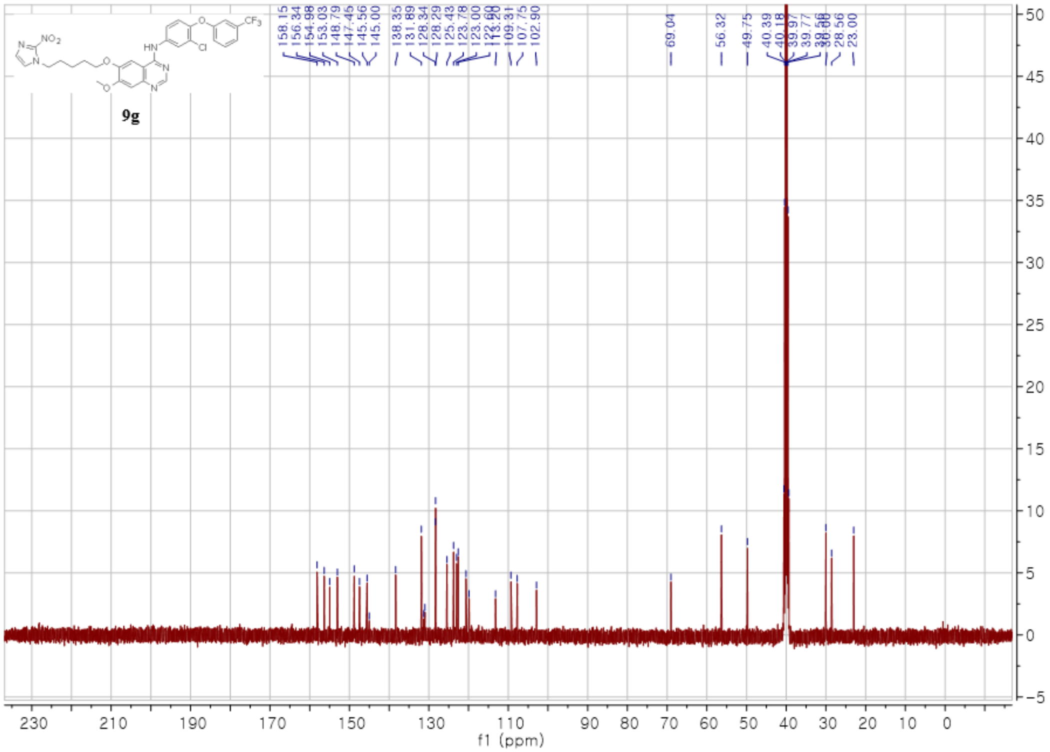

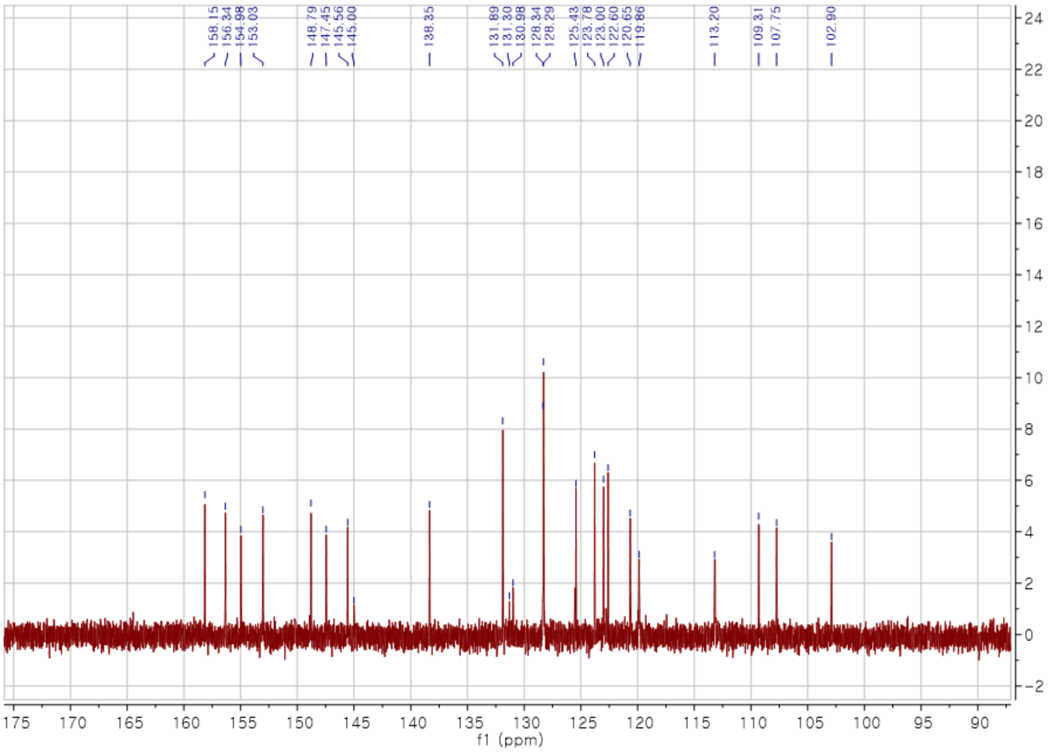

Figure SI 40. HRMS chart of compound **9g**

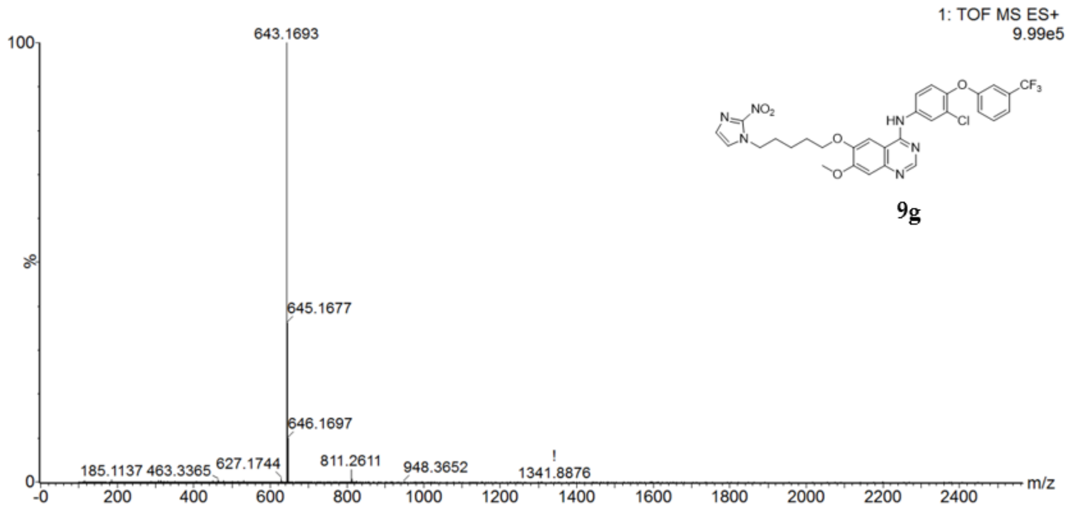

Figure SI 41. HPLC purity chart of compound **9g**

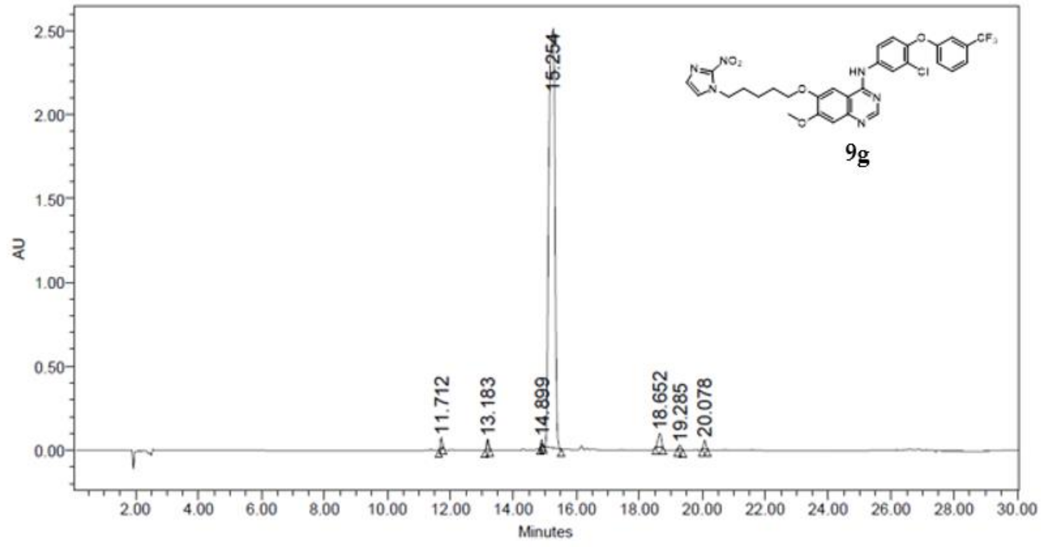

	RT	Area	% Area	Height
1	11.712	232684	0.66	60211
2	13.183	259472	0.73	60117
3	14.899	91894	0.26	30285
4	15.254	33887004	95.59	2519624
5	18.652	588299	1.66	79954
6	19.285	122749	0.35	22778
7	20.078	269252	0.76	51617

Figure SI 42. ^{1}H NMR spectrum of compound **9h**

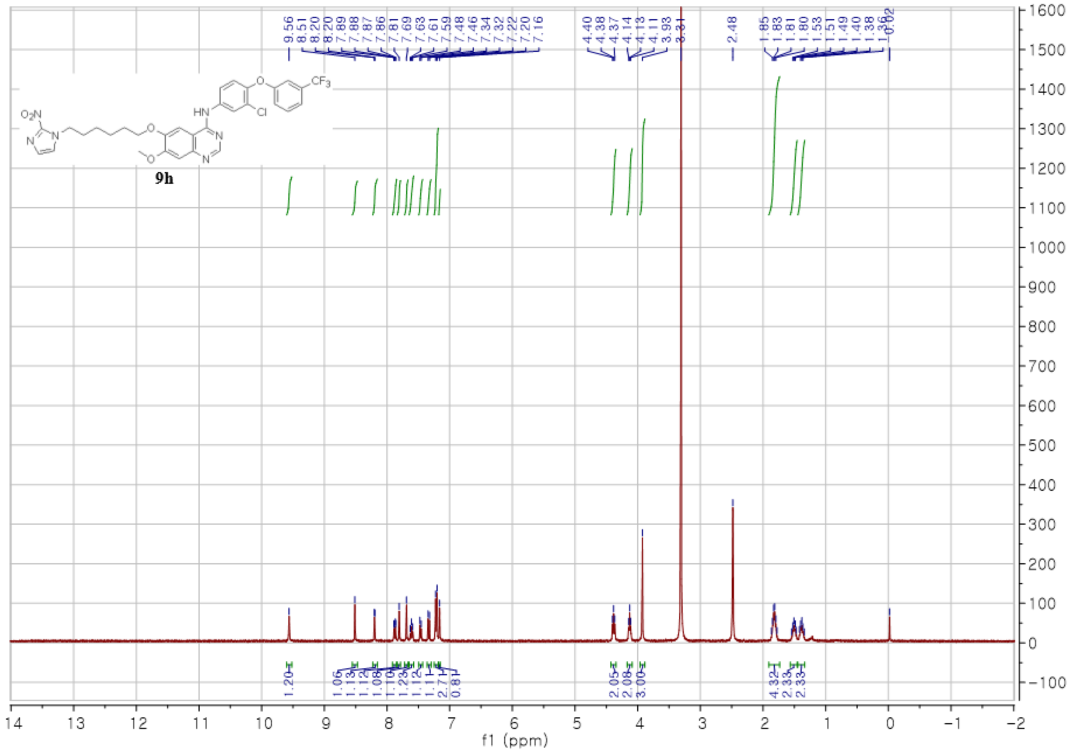

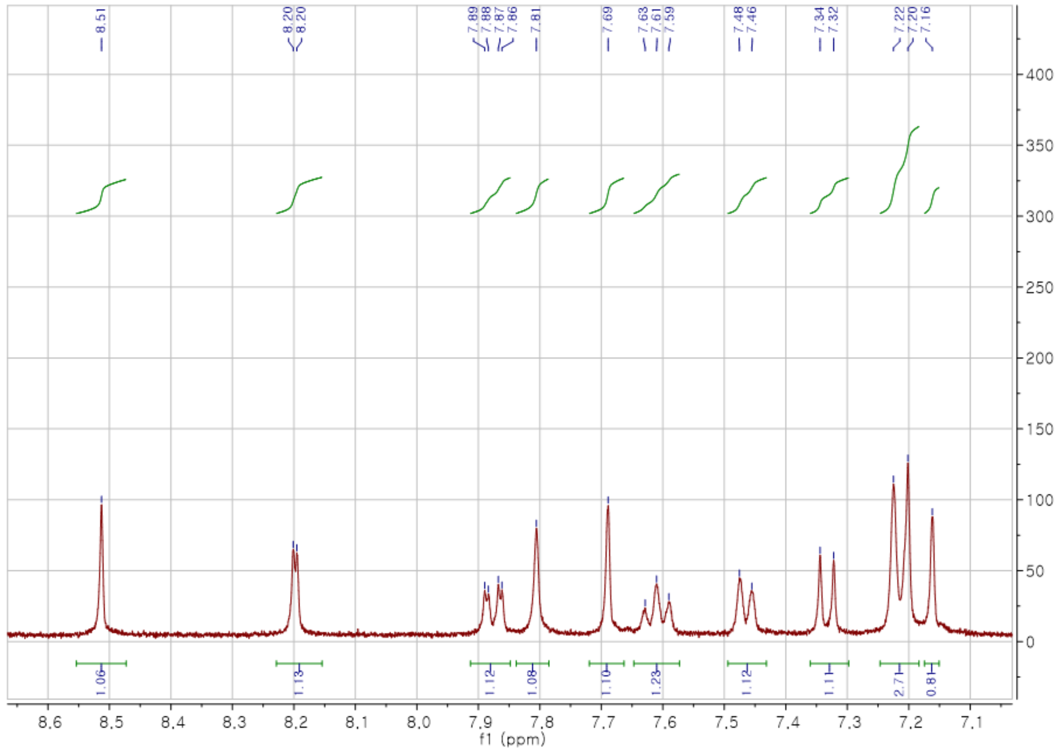

Figure SI 43. ^{13}C NMR spectrum of compound **9h**

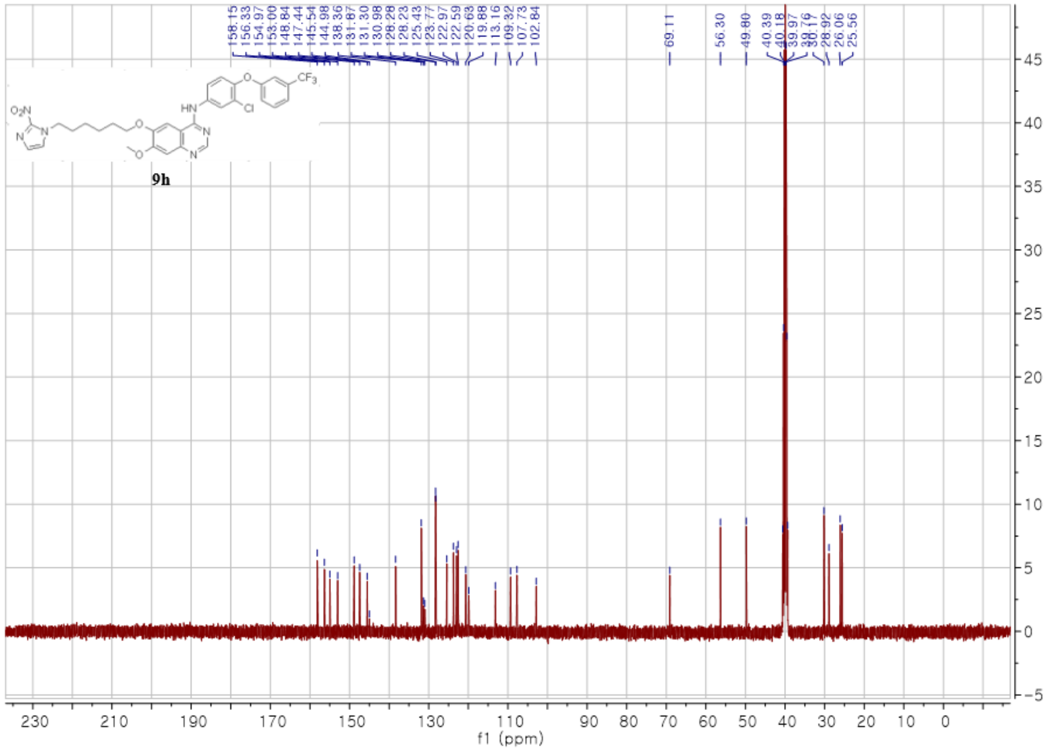

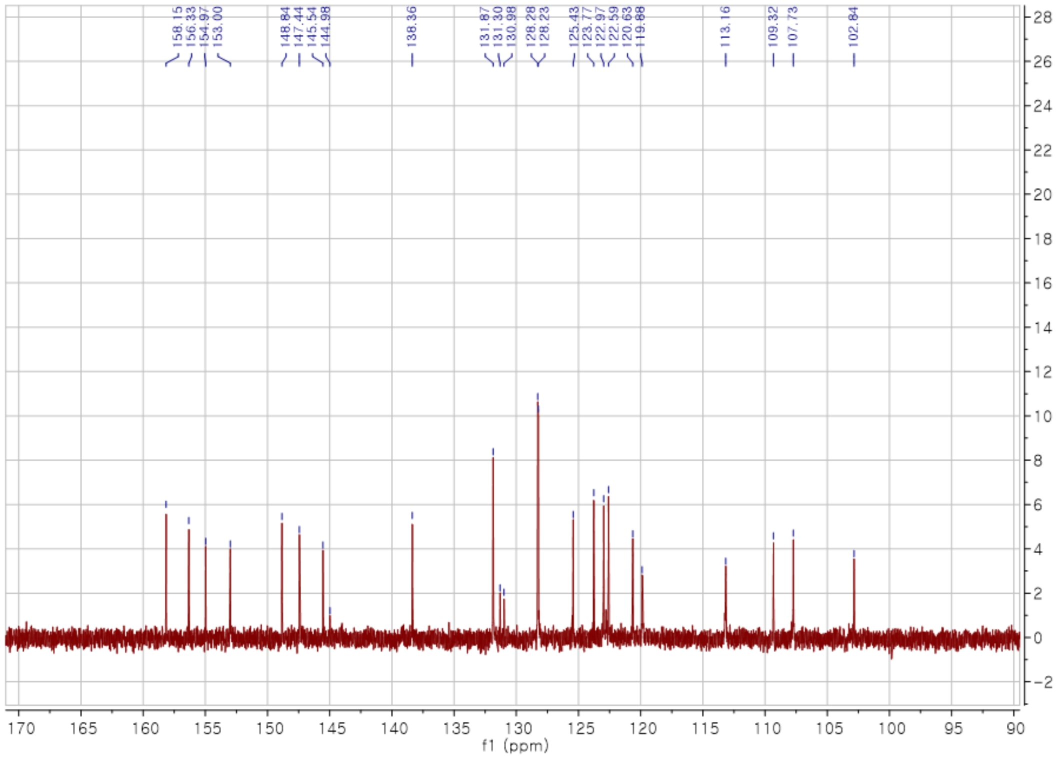

Figure SI 44. HRMS chart of compound **9h**

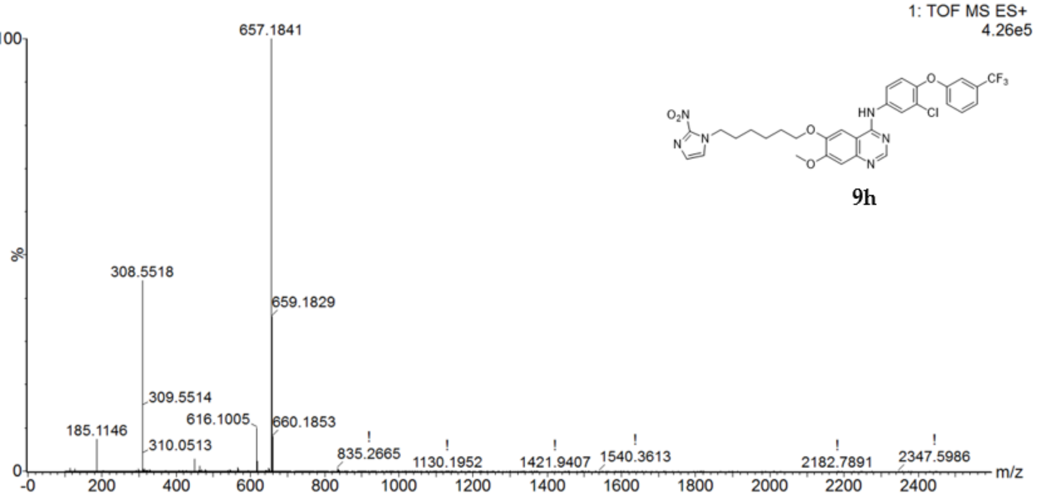

Figure SI 45. HPLC purity chart of compound **9h**

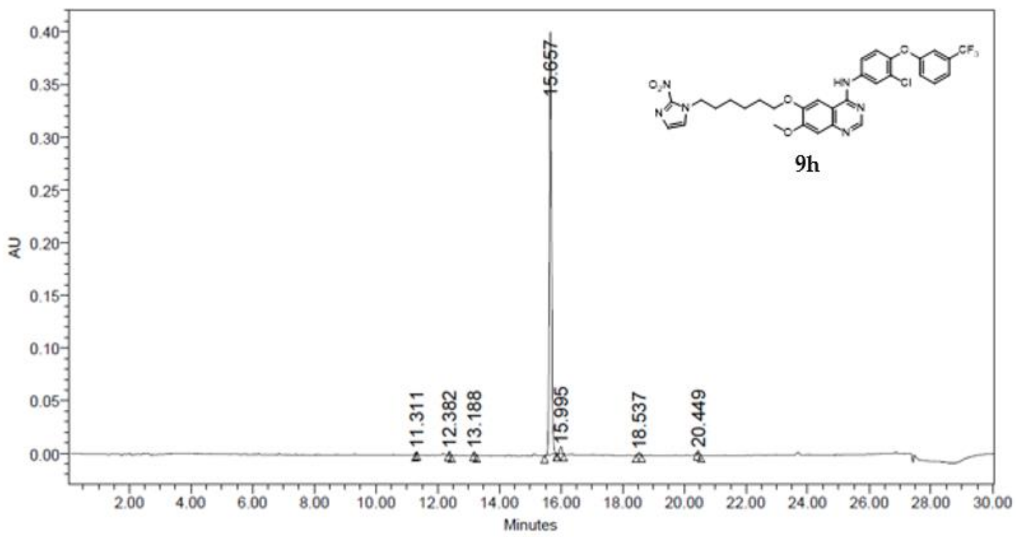

	RT	Area	% Area	Height
1	11.311	3658	0.18	1430
2	12.382	9869	0.49	2676
3	13.188	6103	0.31	1943
4	15.657	1919610	96.10	394784
5	15.995	34896	1.75	6388
6	18.537	13226	0.66	2416
7	20.449	10134	0.51	2386

References

1. Release, S., *3: Desmond molecular dynamics system, DE Shaw research, New York, NY, 2017*. Maestro-Desmond Interoperability Tools, Schrödinger, New York, NY, 2017.
2. Harder, E., et al., *OPLS3: a force field providing broad coverage of drug-like small molecules and proteins.* Journal of chemical theory and computation, 2016. **12**(1): p. 281-296.
3. Jorgensen, W.L., et al., *Comparison of simple potential functions for simulating liquid water.* The Journal of chemical physics, 1983. **79**(2): p. 926-935.
4. Neria, E., S. Fischer, and M. Karplus, *Simulation of activation free energies in molecular systems.* The Journal of chemical physics, 1996. **105**(5): p. 1902-1921.
5. Manual, D.U., *Desmond2. 2*. 2009.
6. Martyna, G.J., M.L. Klein, and M. Tuckerman, *Nosé–Hoover chains: The canonical ensemble via continuous dynamics.* The Journal of chemical physics, 1992. **97**(4): p. 2635-2643.
7. Martyna, G.J., D.J. Tobias, and M.L. Klein, *Constant pressure molecular dynamics algorithms.* The Journal of chemical physics, 1994. **101**(5): p. 4177-4189.
